# Internal Transcription Terminators Control Stoichiometry of ABC Transporters in Cellulolytic Clostridia

**DOI:** 10.1128/spectrum.01656-21

**Published:** 2022-03-14

**Authors:** Shasha Wu, Mengcheng You, Na Wang, Zhenxing Ren, Chenggang Xu

**Affiliations:** a Key Laboratory of Chemical Biology and Molecular Engineering of Ministry of Education, Institute of Biotechnology, Shanxi University, Taiyuan, Shanxi Province, China; b Institute of Applied Chemistry, Shanxi University, Taiyuan, Shanxi Province, China; College of New Jersey

**Keywords:** ABC importer, stoichiometry, stem-loop structure, internal transcription terminator, *Ruminiclostridium cellulolyticum*

## Abstract

The extracellular substrate-binding proteins (SBPs) of ATP-binding cassette (ABC) importers tend to be expressed in excess relative to their cognate translocators, but how the stoichiometry of ABC transporters is controlled remains unclear. Here, we elucidated a mechanism contributing to differential gene expression in operons encoding ABC importers by employing cellulolytic Clostridia species, specifically Ruminiclostridium cellulolyticum. We found that there were usually stem-loop structures downstream of SBP genes, which could prematurely terminate the transcription of ABC importers and were putative internal intrinsic terminators, resulting in high transcript levels of upstream SBP genes and low transcript levels of downstream cognate translocator genes. This was determined by their termination efficiencies. Internal terminators had a lower U content in their 3′ U-rich tracts and longer GC-rich stems, which distinguishes them from canonical terminators and potentially endows them with special termination efficiencies. The pairing of U-rich tracts and the formation of unpaired regions in these internal terminators contributed to their folding energies, affecting the stability of their upstream SBP transcripts. Our findings revealed a strategy of internal transcriptional terminators controlling *in vivo* stoichiometry of their flanking transcripts.

**IMPORTANCE** Operons encoding protein complexes or metabolic pathways usually require fine-tuned gene expression ratios to create and maintain the appropriate stoichiometry for biological functions. In this study, a strategy for controlling differential expression of genes in an operon was proposed by utilizing ABC importers from Ruminiclostridium cellulolyticum. We found that a stem-loop structure is introduced into the intergenic regions of operons encoding ABC importers as the putative internal terminator, which results in the premature termination of transcription. Consequently, the stoichiometric ratio of genes flanking terminators is precisely determined by their termination efficiencies and folding energies at the transcriptional level. Thus, it can be utilized as a promising synthetic biology tool to control the differential expression of genes in an operon.

## INTRODUCTION

ATP-binding cassette (ABC) transporters, found in both prokaryotes and eukaryotes, play an important role in various physiological processes, such as the uptake of nutrients, facilitating multidrug resistance, the secretion of signal molecules or toxins, cell volume regulation, and other processes (1–3). In general, it is assumed that the core of an ABC transporter consists of two transmembrane domains (TMDs) and two nucleotide-binding domains (NBDs) (4). These TMDs form a translocation pathway for transporting substrates across a membrane, while the NBDs are involved in ATP binding and hydrolysis (5).

While the substrate specificity of ABC exporters is guided by their TMDs, canonical prokaryotic ABC importers are dependent on an additional extracellular substrate-binding protein (SBP) that delivers a substrate to its translocator. These can be periplasmic in Gram-negative bacteria, membrane-tethered in Gram-positive bacteria, or fused to the permease (5). SBPs have a bilobed structure where substrate binding occurs between the two symmetrical lobes (6, 7). SBPs productively interact with a cognate importer, thereby triggering ATPase activity and initiating the transport cycle (8–11). In general, SBPs show increased abundance compared with other proteins in prokaryotic cells, and their amount can reach millimole concentrations in the Gram-negative periplasm (12). In fact, in some cases, SBPs can compose up to 40% of Gram-positive surface lipoproteins (13). Furthermore, the number of SBPs in cells greatly exceeds the number of its cognate membrane components. For instance, SBPs for maltose in Escherichia coli and histidine in Salmonella enterica were found to exist in a 30 to 50-fold excess relative to their cognate translocators (14). However, most prokaryotic ABC genes are contained in operons or clusters, but how the stoichiometric differences between SBPs and their cognate translocators are controlled is unclear.

In nature, cellulolytic microorganisms can degrade lignocellulose into various monosaccharides (including pentose and hexose), disaccharides, and oligosaccharides (15, 16), and they have evolved many transporter systems for the adsorption of degraded carbohydrates (17). Cellulolytic Clostridia species usually employ ABC transporters but not phosphoenolpyruvate-carbohydrate phosphotransferase (PTS) for the adsorption of sugars (18–20). For example, many ABC transporters have been found in the genome of Ruminiclostridium (Clostridium) cellulolyticum, but no genes encoding the PTS system have been predicted (21).

In this study, we first predicted and analyzed gene clusters encoding ABC importers in eight anaerobic cellulolytic Clostridia species. We found that 99.5% of SBP genes were located at both ends of gene clusters, especially at the 5′ end. Moreover, stem-loop structures were frequently found immediately downstream of 5′ SBP genes. We further determined the role of these stem-loops in controlling the stoichiometry of ABC importers by employing R. cellulolyticum H10 as a model system. All 16 stem-loop structures predicted in R. cellulolyticum could cause premature transcription termination of ABC importer gene clusters at a certain efficiency and function as putative internal intrinsic terminators. However, the stoichiometric ratio between upstream SBPs and the downstream cognate translocators of stem-loops was not only determined by the transcription efficiency of these stem-loops but was also affected by the folding energy of stem-loops. This was related to the stability of monocistronic transcripts of SBPs. The folding energy of stem-loops was decreased by the pairing of 3′ U-rich tracts, and it was increased by the formation of unpaired regions in stems. Our findings thus unveiled a mechanism linking stem-loop structures and the stoichiometry of transcripts in an operon and have general implications for the designing and engineering of metabolic pathways or protein complexes with specific stoichiometric ratios.

## RESULTS

### SBP genes are usually located at the 5′ end of gene clusters of ABC importers.

**FIG 1:**
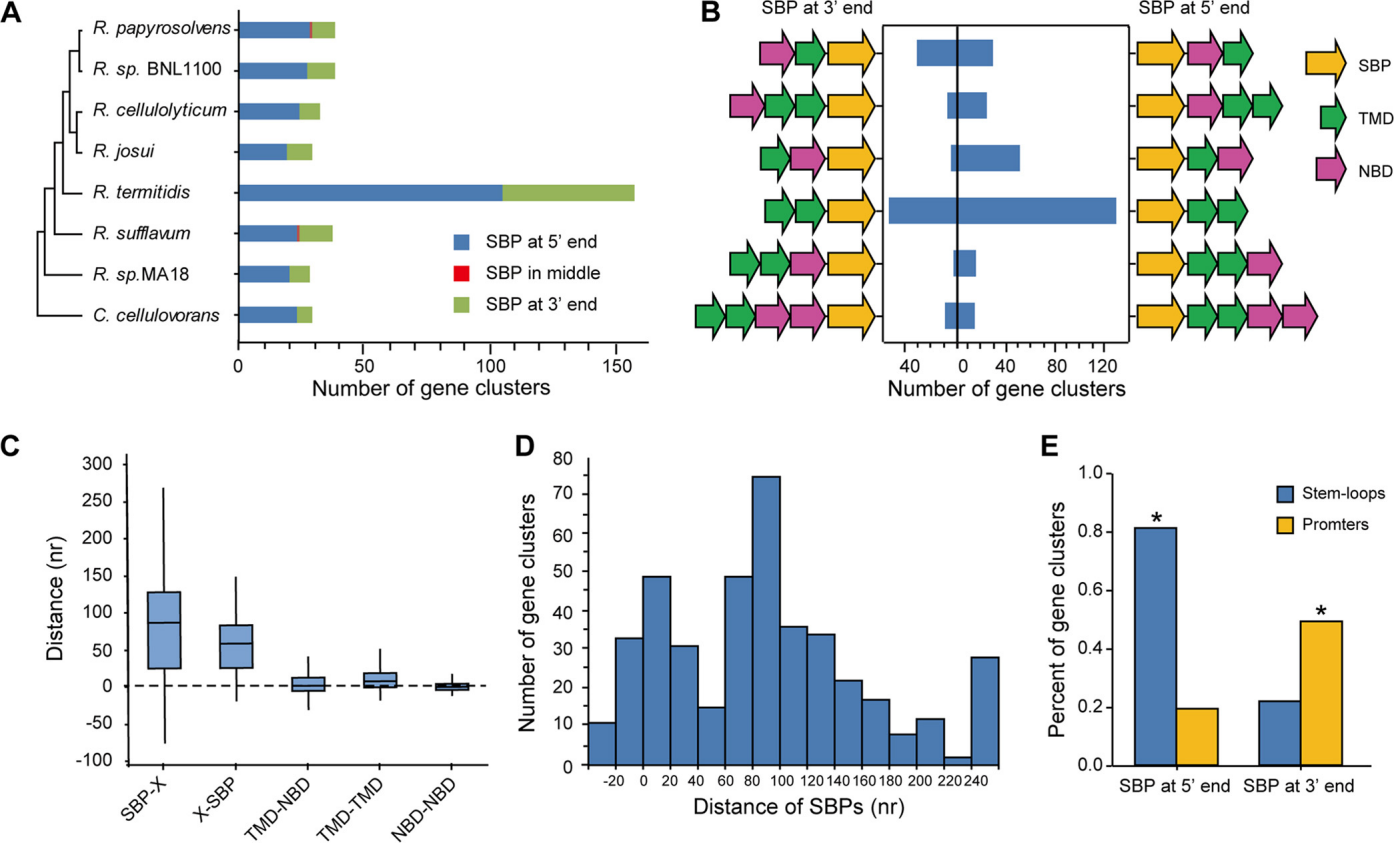
Characteristics of the organization of gene clusters encoding ABC importers from mesophilic cellulolytic Clostridia. (A) The numbers of gene clusters of ABC transporters harboring SBPs from R. papyrosolvens, *R.* sp. BNL1100, R. cellulolyticum, R. josui, R. termitidis, R. sufflavum, *R.* sp. MA18, and C. cellulovorans were compared, and clusters were classified into three types according to the position of SBPs in these clusters: 5′ end, middle, and 3′ end. (B) The distribution of gene clusters in various gene arrangements, where the clusters harboring two genes were not shown because their number was too small to generalize. (C) Comparison of the distances between two adjacent genes in gene clusters, including distances between SBPs and its downstream (SBP-X) or upstream gene (X-SBP). (D) The distribution of distances of SBPs to their adjacent gene TMDs or NBDs. (E) Prediction of the stem-loops and promoters in IRs between SBPs and their cognate translocator genes. The stem-loops and promoters were enriched in 5′ SBP clusters and 3′ SBP clusters, respectively (*, *P *< 0.0001, hypergeometric test).

A total of 387 ABC importer gene clusters harboring SBPs were predicted in eight cellulolytic Clostridia, including R. cellulolyticum H10, Ruminiclostridium papyrosolvens DSM2782, Ruminiclostridium sp. BNL1100, Ruminiclostridium josui JCM17888, Ruminiclostridium termitidis CT1112, Ruminiclostridium sp. MA18, Ruminiclostridium sufflavum DSM 19573, and Clostridium cellulovorans 743B (Table S1). We found that the number of gene clusters encoding ABC importers in cellulolytic Clostridia ranged from 29 to 38, except for R. termitidis CT1112, which harbored 156 clusters. According to the position of SBPs in these gene clusters, ABC importer gene clusters could be classified into three types of clusters, namely, those with 5’ end SBPs, 3′ end SBPs, or middle SBPs. However, the majority of SBP genes (99.5%) were located at both ends of clusters, and the number of SBPs located at the 5′ end was much higher than that of SBPs located at the 3′ end in all test Clostridia (Fig. 1A). These gene clusters harbored a different number of genes ranging from two to five, with various permutations and combinations of SBPs, TMDs, and NBDs. Among them, 186 clusters harboring one SBP gene and two TMD genes (130 clusters with 5’ SBPs and 56 clusters with 3’ SBPs) accounted for the biggest share of clusters with 5’ SBPs or 3’ SBPs (Fig. 1B).

Furthermore, the distances between the adjacent genes from clusters were analyzed. These indicated that compared with the seamless linkage between genes encoding TMDs and NBDs, there were wide variations in the distances of SBPs from others, with an average length of 50 to 80 bp (Fig. 1C). Intriguingly, the distribution of distances between SBPs and TMDs or NBDs showed two peaks that ranged from 0 to 20 bp and from 60 to 100 bp, respectively, suggesting that a few SBPs were closely linked with TMDs and NBDs. This was like the linkage between TMD and NBD genes, but others are separated from TMDs and NBDs (Fig. 1D). Altogether, the organization of gene clusters reveals that SBPs usually located at both ends of gene clusters encode ABC importers with relative independence.

Moreover, to find potential elements in the intergenic regions (IRs) between SBPs and their cognate translocator genes, putative stem-loop structures and promoters were predicted in 306 long intergenic regions (more than 50 bp). A total of 213 IRs were predicted to harbor stem-loops (folding energy less than −10 kcal/mol). It showed that 87.3% of IRs (193) from the 5′ SBP clusters harbored stem-loops, which is much more than 23.5% of IRs (20) from the 3′ SBP clusters. However, only 20.8% of IRs (46) from the 5′ SBP clusters were predicted to harbor promoters while 52.9% of IRs (45) from the 3′ SBP clusters harbored promoters (Fig. 1E). Thus, the stem-loops were specifically associated with 5′ SBP clusters, while the promoters were associated with 3′ SBP clusters (*P* < 0.0001; hypergeometric test).

### SBPs were transcribed at much higher levels than genes of cognate translocators.

**FIG 2:**
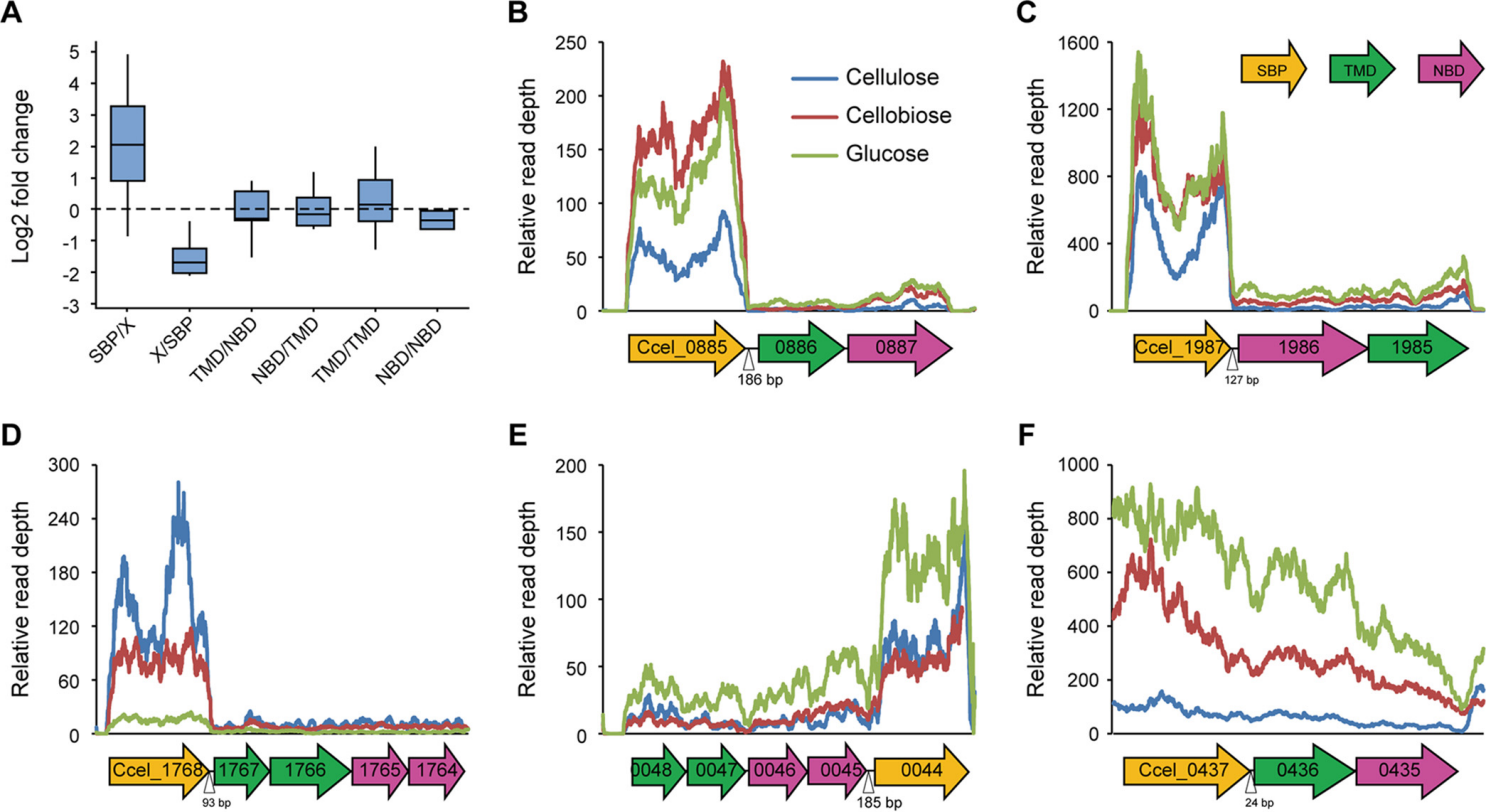
Transcription analysis of gene clusters encoding ABC importers from R. cellulolyticum. (A) Transcriptional differences between two adjacent genes in gene clusters were compared. The fold changes were calculated by the logarithm (base 2) of the ratio of transcription level of upstream genes to downstream genes. (B to F) Transcription profiles of ABC importers harboring 5′-end SBPs (B, C, and D) and 3′-end SBPs (E) with long intergenic regions or 5′-end SBPs with short intergenic regions (F). The length of intergenic regions between SBPs and their adjacent genes is shown.

To probe the effects of the organization of gene clusters on their stoichiometry, the transcription profiles of ABC importers from R. cellulolyticum grown on glucose, cellobiose, and cellulose were compared relative to our previous data (accession number GSE57652). The average fold change of SBPs compared to their downstream or upstream genes (SBP/X or X/SBP) was significantly higher than that between TMDs and NBDs (around zero) (Fig. 2A). This finding suggested that whether SBPs were located at the 5′ end or 3′ end of clusters, SBPs tended to be transcribed at much higher levels than other genes of clusters. For example, the gene clusters Ccel_0885-0887, Ccel_1987-1985, and Ccel_1768-1764, respectively, harbored SBPs (Ccel_0882, Ccel_1987, and Ccel_1768) in their 5′ ends with more than 90-bp intergenic regions compared to their downstream genes and were transcribed to fall off a cliff after SBPs (Fig. 2B to D). Additionally, in the cluster Ccel_0048-0044, its SBP (Ccel_0044) located at the 3′ end was also transcribed to much higher levels than its upstream genes (Fig. 2E). A putative promoter was predicted in the upstream of Ccel_0048 and Ccel_0044, suggesting that the higher transcription level of Ccel_0044 encoding 3′ SBP might be caused by itself promoter (Table S6). It was further confirmed by analysis of promoter activity, in which the promoter activity of Ccel_0044 was almost three times higher than that of Ccel_0048 (Fig. S1). However, when 5′ end SBPs were closely linked with their downstream genes, there were no significant differences in transcript abundances between SBPs and their cognate translocators. For example, Ccel_0437, encoding SBPs in cluster Ccel_0437-0435, had only 24 bp of distance to its downstream genes and was continuously transcribed with its downstream genes (Fig. 2F). Thus, this suggested that the relatively long intergenic regions between SBPs and their downstream genes potentially harbor an element to control the stoichiometric ratio between upstream and downstream genes.

**FIG 3:**
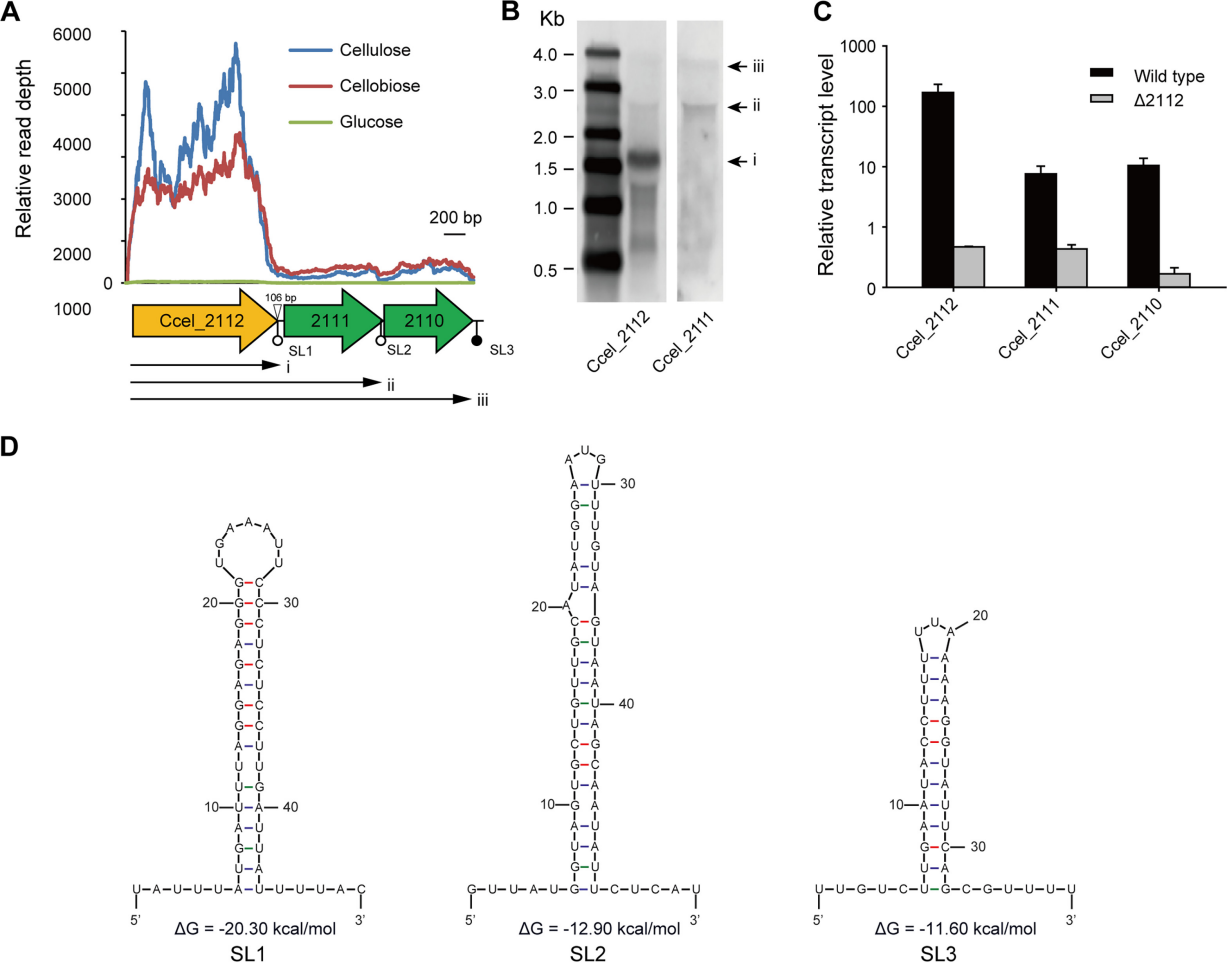
Transcription of gene cluster Ccel_2112-2110 is prematurely terminated in intergenic regions. (A) Transcription profiles of Ccel_2112-2110 under different carbon sources, glucose, cellobiose, and cellulose. (B) Northern blotting of transcripts of Ccel_2112-2110 using Ccel_2112 and Ccel_2111-targeting probes, respectively. For comparison, both results were put side by side based on the ladders. (C) Transcription abundance of gene cluster Ccel_2112-2110 in Ccel_2112-disrupted mutant measured via quantitative reverse transcription-PCR (qRT-PCR) with wild type as a control. Error bars indicate the standard deviation (s.d.) of the mean of experiments performed in triplicate. (D) Stem-loop structures were predicted at the downstream ends of Ccel_2112, Ccel_2111, and Ccel_2110, respectively.

Furthermore, the cluster Ccel_2112-2110 was employed to analyze the transcription pattern of the ABC importer harboring 5′-end SBPs. This cluster harbors an SBP gene (Ccel_2112) at the 5′ end, followed by two TMD genes (Ccel_2111 and Ccel_2110), and has a 106-bp long intergenic region between Ccel_2112 and Ccel_2111. Ccel_2112, encoding SBPs, was transcribed at significantly higher levels than its downstream genes (Fig. 3A). Northern blotting with probes targeting Ccel_2112 and Ccel_2111 revealed that besides the abundant monocistronic transcripts of Ccel_2112, polycistronic transcripts of approximately 2.2 and 3.0 kb was weakly detectable, agreeing with the expected sizes of Ccel_2112-2111 and Ccel_2112-2110, respectively (Fig. 3B). To rule out the presence of within-cluster promoters, Ccel_2112, the first gene of this cluster, was disrupted by Clostron (22). In the resulting mutant, the transcription of genes downstream of the insertion in the cluster was greatly reduced (Fig. 3C). Thus, these results demonstrated that the cluster Ccel_2112-2110 was an operon, and the transcripts of Ccel_2112-2110 were terminated at the 3′ ends of Ccel_2112, Ccel_2111, and Ccel_2110. Subsequently, three stem-loop structures were predicted at the 3′ ends of Ccel_2112, Ccel_2111, and Ccel_2110 (Fig. 3D), suggesting that they potentially terminate the transcription of the cluster of Ccel_2112-2110 as Rho-independent terminators, which could explain this cluster’s transcriptional patterns.

### Stem-loops downstream of SBPs cause premature termination of transcription.

**FIG 4:**
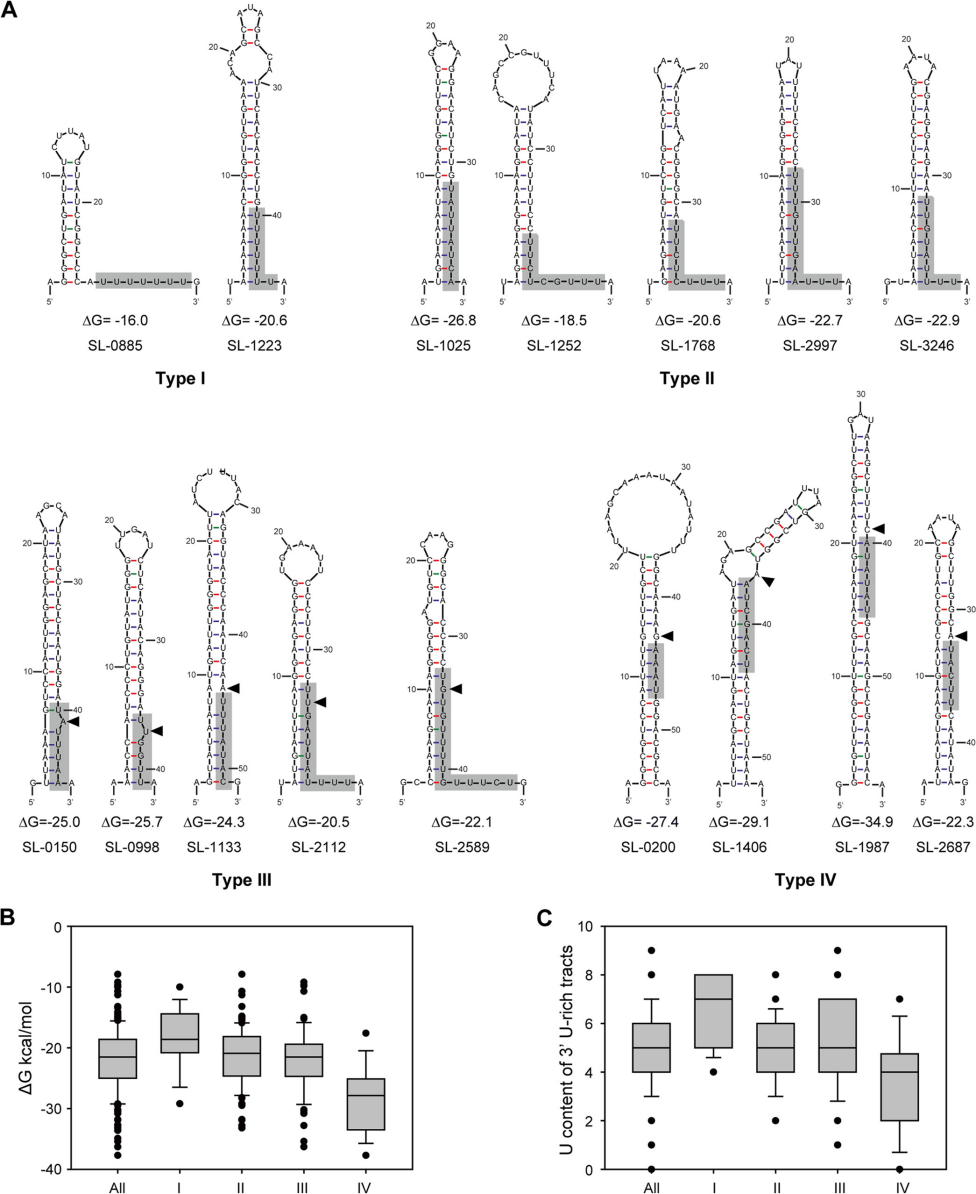
Stem-loop structures predicted downstream of SBPs. (A) Stem-loops predicted in gene clusters encoding ABC importers from R. cellulolyticum, which were classified into four types based on their structural features. Putative 3′ U-rich sequences are shown in gray boxes. (B and C) Comparison of the RNA folding free energy (ΔG) (B) and 3′ U content (C) of four types of stem-loop structures from all eight Clostridia.

To find potential elements controlling the stoichiometry of ABC transporters, C-rich regions (the potential binding sites of Rho factor), and the RNA secondary structures of intergenic regions between SBPs and their downstream genes were further analyzed. It was found that the long intergenic regions tended to harbor a stem-loop structure but not a C-rich region, suggesting Rho-independent termination. In total, 191 putative stem-loop structures were found, and their folding energy ΔG ranged from −38.3 to −10.1 kcal/mol in eight cellulolytic Clostridia (Table S2). Based on these structures, the stem-loops could be classified into four types: 24 Type I, which had the typical structure of Rho-independent terminators harboring a paired or unpaired 3′ poly(U) tails and the number of U in poly(U) tails of Type I stem-loops was more than 5; 88 Type II, which were similar to Type I, except that they replaced the poly(U) tail with a 3′ U-rich tract and the number of U in 3’tail of stem-loops was less than 5; 62 Type III, which harbored a pair of 3′ U-rich tracts following a 1 to 2 base unpaired region; and 17 Type IV, which were similar to Type III but had a longer paired region with a lower content of 3′ U following the unpaired region. Thus, sequences at the 3′ paired or unpaired U-rich tails for Type I and Type II stem-loops and downstream of unpaired regions in Types III and IV were considered to be putative 3′ U-rich tracts of Rho-independent terminators (Table S2). For instance, R. cellulolyticum had two Type I, five Type II, five Type III, and four Type IV stem-loops (Fig. 4A). The differences in the structure of stem-loops led to differences in their folding free energy. Type IV stem-loop structures had the lowest average folding free energy at −28.4 kcal/mol, while Type I had the highest, at −18.3 kcal/mol (Fig. 4B). In contrast, the results of comparisons of U content of 3′ U-rich tracts indicated that the U content of the 3′ U-rich tracts of Type I stem-loops ranged from 5 to 8 as the highest, while Type IV stem-loops tended to be the lowest, as most of these hardly had obvious U-rich regions (Fig. 4C). Given the structural features of these stem-loops and the transcriptional profiles of ABC importers, we speculated that these stem-loops could cause transcription to terminate as internal transcriptional terminators.

**FIG 5:**
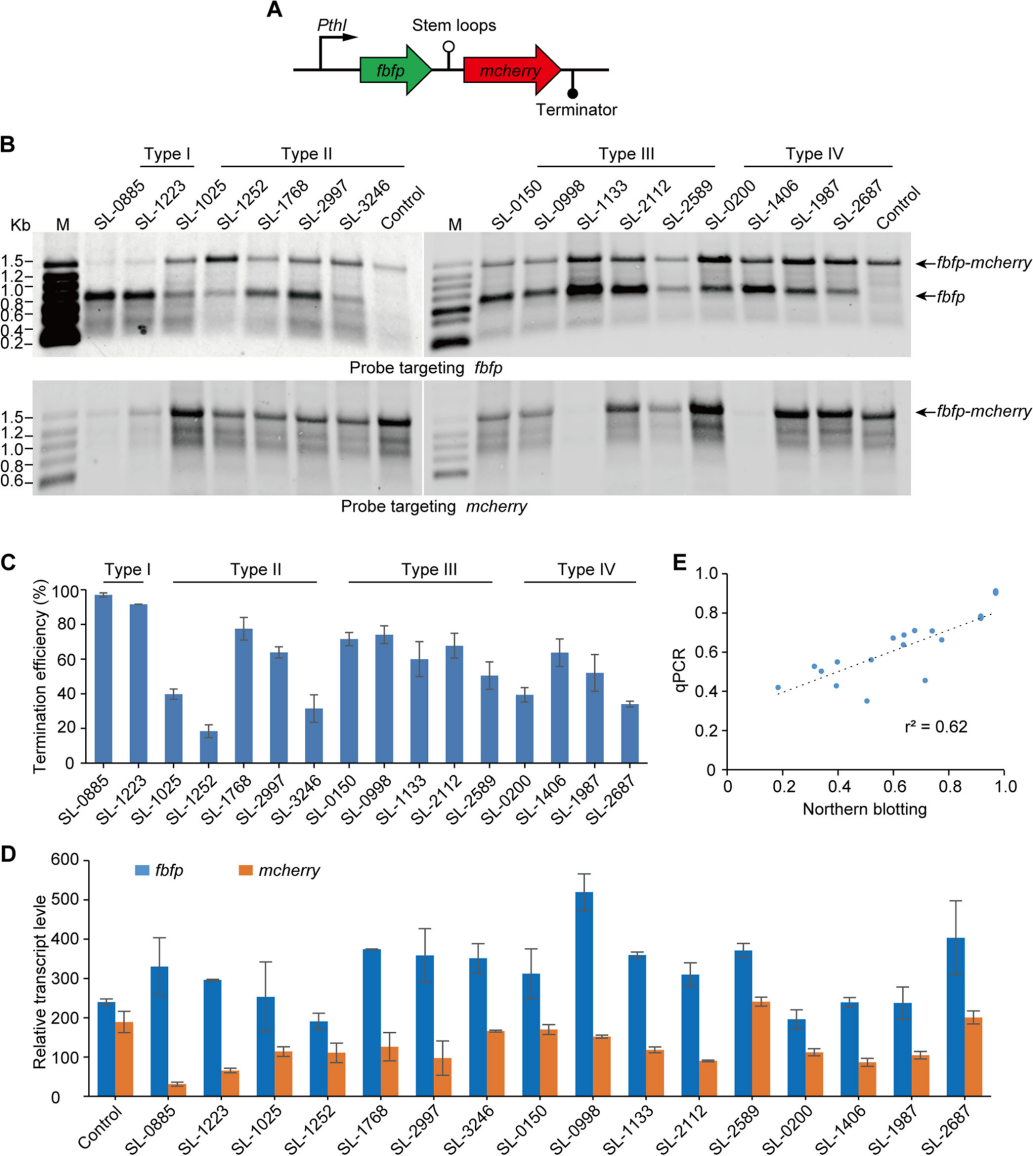
Functional analysis of stem-loops from R. cellulolyticum using an *fbfp-mcherry* artificial operon. (A) Schematic representation of this dual-fluorescence reporter system for functional analysis of stem-loop structures, where stem-loops were inserted between *fbfp* and *mcherry*, respectively. (B) Northern blotting of transcripts carrying *fbfp* and *mcherry* from artificial operons harboring various stem-loops using *fbfp*- and *mcherry*-targeting probes, respectively. Black arrows highlight the positions of bands that corresponded to transcripts, as indicated on the right side of the panel. (C) Transcription TE was calculated by comparison of the abundance between the monocistronic transcript *fbfp* and the bicistronic transcript *fbfp-mcherry*. Transcript abundance was determined by the gray value of bands in Northern blotting using ImageJ software (58). (D) The relative transcription levels of *fbfp* and *mcherry* in artificial operons harboring various stem-loops measured by qPCR. (E) Correlation of the transcription TE of stem-loops determined by Northern blotting with that from qPCR (r^2^ = 0.62). Error bars indicate the standard deviation of the mean from experiments done in triplicate.

To probe this hypothesis, an experiment testing the *in vivo* role of these stem-loops was designed, where these four types of stem-loops from R. cellulolyticum were inserted between the reporter genes *fbfp* (encoding a green fluorescence protein) and *mcherry* (encoding a red fluorescence protein; Fig. 5A). The resulting artificial operon (or the control, where no segments were inserted) was then separately introduced into R. cellulolyticum. Using Northern blotting, two probes were designed to detect transcripts that harbored *fbfp* or *mcherry*. Only a single 1.2-kb band from the control was detected by both probes, consistent with the expected size of a bicistronic transcript of *fbfp-mcherry*. However, in addition to the full-size band of the bicistronic transcript, a monocistronic transcript of *fbfp*, with its predicted size, was also detected in each artificial operon that carried stem-loops when the probe targeted *fbfp*, suggesting that the introduction of stem-loops resulted in premature termination of transcription (Fig. 5B). However, termination efficiency (TE) was calculated by comparing the abundances of monocistronic transcripts of *fbfp* as prematurely terminated transcripts and bicistronic transcripts of *fbfp-mcherry* as read-through transcripts. The results showed that these stem-loops had different TEs, which ranged from 20.7% to 96.1%. Among them, the stem-loops of Type I (SL-0885 and SL-1223) had the highest TEs, which were more than 90%, while the TEs of SL-1025, 1252, 3246, 2589, 0220, and 2687 were lower than 50% (Fig. 5C). Furthermore, the relative transcript levels of *fbfp* and *mcherry* from these artificial operons were measured by qPCR. This analysis revealed that the transcript level of *mcherry* downstream of stem-loops was significantly lower than that of the upstream *fbfp*, especially in the case of SL-0885 and 1223, where the ratios of the transcript levels of *mcherry* to *fbfp* only reached 9.30% and 20.73%, respectively. This finding was consistent with the observation above by Northern blotting (Fig. 5D). The TE of stem-loops was further examined using our qPCR results, and these significantly correlated with our data from Northern blotting (Fig. 5E, r^2^ = 0.62). Therefore, stem-loop structures could cause premature termination of transcription and function as transcriptional terminators, allowing a percentage of transcripts to read through and resulting in a specific transcriptional stoichiometry between flanking genes.

### Intrinsic structure of stem-loops determines the stoichiometric ratio of their flanking transcripts.

By comparing the stem-loop structures from clusters of these ABC importers, we found that these stem-loops had three features: 3′ U-rich tracts, long stems, and middle unpaired regions, all of which could affect the stoichiometric ratio between both flanking transcripts.

**FIG 6:**
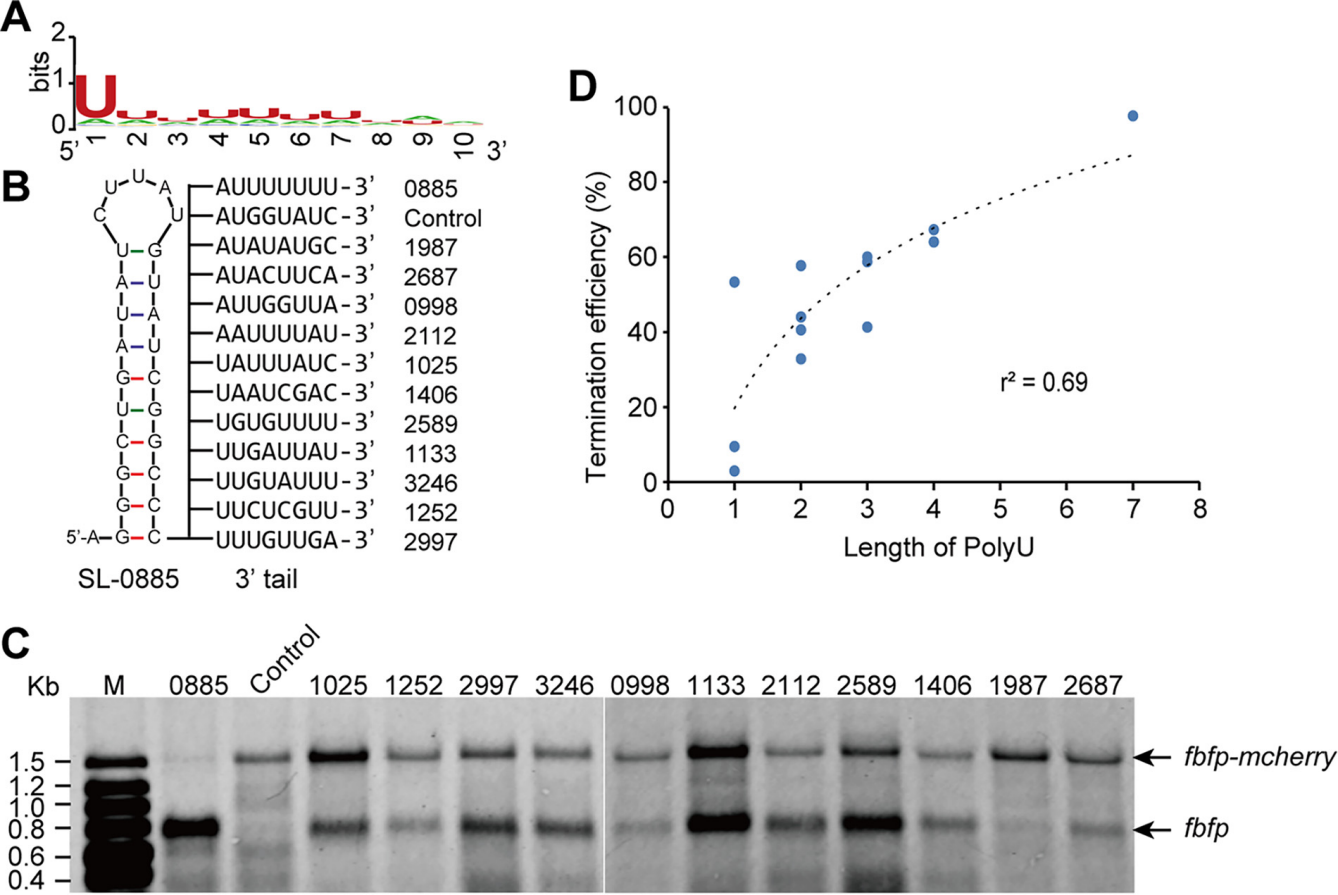
Effect of 3′ U-rich tails in stem-loops on transcription termination. (A) WebLogo analysis of the frequency of base distribution for 3′ U-rich sequences in stem-loops. The relative sizes of letters indicate their frequency in these sequences. The total height of the letters depicts the information content of each position in bits. (B) Schematic diagram of SL-0885 fused with different 3′ tails from other stem-loops. The SL-0885 with no tails was used as a control, and its downstream sequence from the plasmid vector was listed. (C) Northern blotting of transcripts carrying *fbfp* and *mcherry* from artificial operons harboring SL-0885 with different 3′ tails. SL-0885 with no 3′ tail was a control. (D) Correlation of the transcription TE of stem-loops determined by Northern blotting with the length of poly(U) in 3′ tails (r^2^ = 0.69).

First, WebLogo (23) was used to investigate the conservation of 3′ U-rich tracts of stem-loops. The result showed that there was a specific conserved U in the first seven bases of U-rich tracts from 5′ to 3′ (Fig. 6A). To gain more insights into the functional diversity of 3′ U-rich tracts, the 3′ poly(U) tail of SL-0885, was replaced with various U-rich tracts from other stem-loops and inserted into the *fbfp-mcherry* reporter system, using SL-0885 with no tails as a control (Fig. 6B). Transcription of these artificial operons in R. cellulolyticum was then analyzed using Northern blotting. The results indicated that only a single band of the bicistronic transcript of *fbfp-mcherry* from the control was detected, suggesting that SL-0885 without U-rich tracts was unable to stop transcription. However, when SL-0885 was fused with various 3′ U-rich tracts, monocistronic transcripts of *fbfp* of different intensities were also detected in addition to the full-size bands of bicistronic transcripts. This suggested that the introduction of 3′ U-rich tracts in SL-0885 resulted in premature termination of transcription (Fig. 6C). Furthermore, the transcription TE of SL-0885 with different 3′ tails was determined based on the results of Northern blotting. Intriguingly, the TE correlated significantly with the length of poly(U) in these 3′ U-rich tracts (r^2^ = 0.69) (Fig. 6D). For example, although both 3′ U-rich tracts from SL-1987 and SL-2687 had three U residues, the TE of 3′ U-rich tracts from SL-2687 with its two poly(U) was 32.8 ± 0.4% significantly higher than the 9.4 ± 0.9% of SL-1987 that had no poly(U). This may have been due to the difference in the polymerization of U residues between the 3′ tails of SL-1987 and SL-2687. This suggested that the 3′ tail could control the TE of stem-loops, which was consistent with a recent study on the importance of poly(U) in the 3′ tail for the TE of intrinsic terminators found in Bacillus subtilis (24).

**FIG 7:**
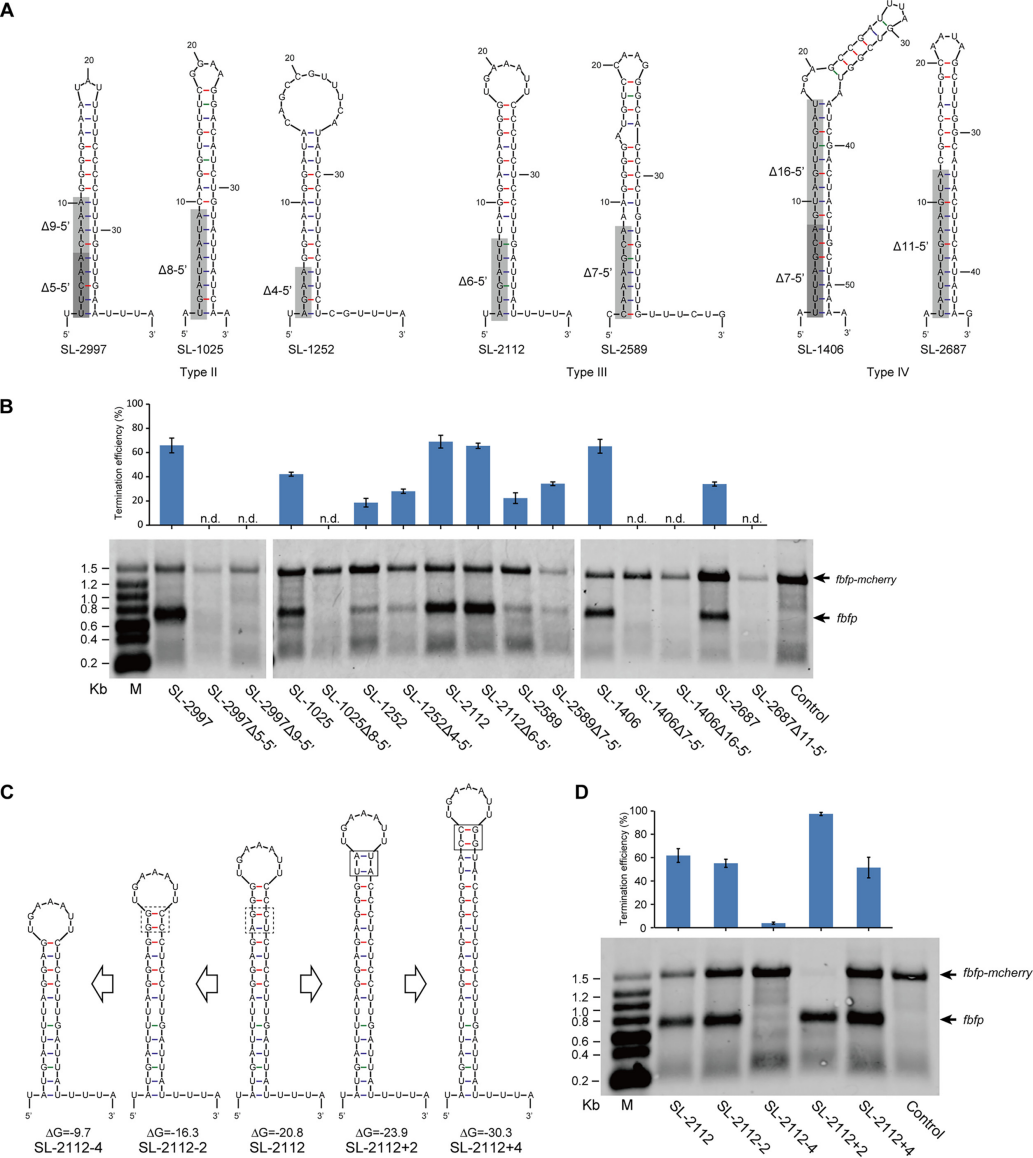
Effects of the stem length on transcription termination. (A) Schematic diagram of deletions of 5′ sequences of stem-loops (SL-2997, 1025, 1252, 2112, 2589, 1406, and 2687) paired with 3′ U-rich regions. The deleted sequences are shown in gray boxes. (B) Northern blotting of transcripts carrying *fbfp* from artificial operons harboring the above stem-loop mutants. Wild-type stem-loops were used as controls. Transcription TE was calculated by comparison of the gray value of the bands of *fbfp* and *fbfp-mcherry*. (C) Schematic diagram of shortening or prolonging the stem of SL-2112. The changed sequences are shown in the box. (D) Northern blotting of transcripts carrying *fbfp* from artificial operons harboring mutants of SL-2112. Wild-type SL-2112 was used as a control. Transcription TE of stem-loops and their derivatives were calculated by comparison of the gray value of the bands of *fbfp* and *fbfp-mcherry*. Error bars indicate the standard deviation of the mean from experiments done in triplicate.

Second, the 3′ U-rich tracts of stem-loops tended to pair with 5′ sequences, resulting in longer stems that could promote the stability of transcripts to RNase digestion but not impact on the TE of stem-loops because it has been reported that pairing between poly(A) and the U-tract is not involved in termination function (25, 26). To test this hypothesis, several stem-loops of different types were selected to delete their 5′ sequences paired with 3′ U-rich regions (Fig. 7A). Surprisingly, the results of Northern blotting indicated that some paired regions with 3′ U-rich sequences were crucial for the transcription termination of stem-loops. For example, when SL-2997 (Type II) was truncated by 5 or 9 bases from its 5′ end (termed SL-2997Δ5-5′ and SL-2997Δ9-5′), transcription was no longer terminated. Similar results were also observed for SL-1025 (Type II) and SL-1406 and SL-2687 (Type IV). However, for SL-2112 and SL-2589 (Type III) and SL-1252 (Type II), the deletion of 5′ sequences did not affect their function in terms of transcription termination (Fig. 7B). We thus speculated that stem-loops required stems with a considerable length for transcription termination to occur. This, however, could not explain the loss of function of SL-2997Δ5-5′, which has a longer stem than the mutants of stem-loops of Type III, suggesting that pairing of the 3′ U-tract with 5′ sequences maybe play an important role in determining the terminator functionality.

In contrast, to further clarify the effects of the stem length on transcription termination, a series of SL-2112 derivatives was designed by shortening or lengthening its stem in the folding energy range from −9.7 to −30.3 kcal/mol (Fig. 7C). The results of Northern blotting indicated that when the stem of SL-2112 was deleted by two and four base pairs on the top (named SL-2112-2 and SL-2112-4), its TE decreased from 61.8% to 55.1% and 3.8%, respectively. On the contrary, when the stem of SL-2112 was increased by two base pairs on its top, its TE significantly increased to 97.4%, which was comparable to that of Type I stem-loops with their long poly(U) tracts. However, the TE decreased to 51.5%, even though the stem was four base pairs longer on top (Fig. 7D). Thus, for the transcription termination of stem-loops, it appeared to be essential to maintain a certain stem length, which has a notable influence on the TE of stem-loops.

**FIG 8:**
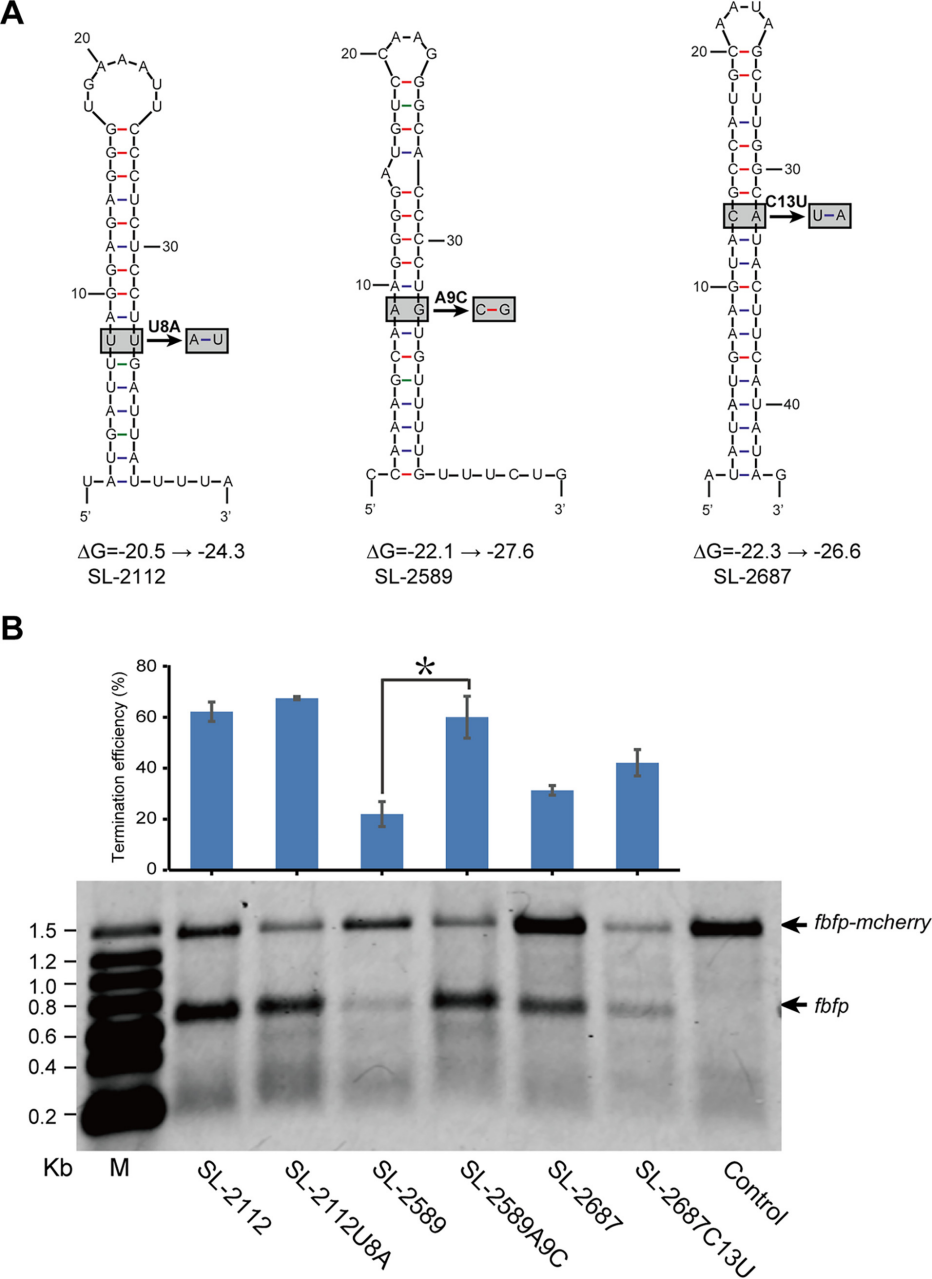
Effects of unpaired regions in stem-loops on transcription termination. (A) Schematic diagram of the replacement of unpaired regions in SL-2112, 2589, and 2687, where unpaired bases were replaced with paired bases. (B) Northern blotting of transcripts carrying *fbfp* from artificial operons harboring the above mutants for replacement of unpaired regions. Wild-type stem-loops were used as a control. Transcription TE was calculated by comparison of the gray value of the bands of *fbfp* and *fbfp-mcherry* (*, *P *< 0.05, Student's *t* test). Error bars indicate the standard deviation of the mean from experiments done in triplicate.

Moreover, we found that each Type III and IV stem-loop had an unpaired region in the middle of the stem. To determine the role of these unpaired regions, SL-2112, SL-2589, and SL-2687 were mutated into paired forms. Mutation of SL-2112 and SL-2687 produced an A-T base pair, while mutation of SL-2589 produced a G-C base pair, resulting in a reduced ΔG ranging from 3.9 to 5.5 kcal/mol (Fig. 8A). It indicated that the introduction of unpaired regions in Type III and IV stem-loops could increase their folding energies, potentially contributing to the stability of their upstream transcripts. However, the results of Northern blotting indicated that the TE of the SL-2589 mutant was significantly increased from 22.00 ± 4.91% to 60.06 ± 8.20%, but not a mutation of SL-2112 and SL-2687 (Fig. 8B). It was suggested that the G-C base pair of stem-loops played an important role in transcription termination.

## DISCUSSION

Many functional protein complexes or metabolic pathways require an appropriate stoichiometry of subunits or enzymes. For example, bacterial membrane-bound ATP synthase (F_1_F_0_) is a protein complex containing eight subunits (namely, α, β, δ, γ, ε, a, b, and c) in a specific stoichiometric ratio of 3:3:1:1:1:1:2:10 (27, 28). However, the genes that share the same function class are generally organized as an operon under the control of a single promoter in bacterial genomes, which is an efficient means to regulate the transcription of multiple genes simultaneously. Thus, to create and maintain the appropriate stoichiometry for biological functions, these operons usually require fine-tuned gene expression ratios.

One strategy employed by bacteria is selective RNA processing and stabilization at the posttranscriptional level, where the primary mRNA transcribed as an operon is processed by nucleases into segments first and then variations in the stability between these segments contribute to differential gene expression (29). A good example of this strategy was demonstrated by the *cip-cel* operon encoding cellulosomal enzymes in R. cellulolyticum found in our previous study (30). In the present study, another strategy for controlling differential gene expression of genes in an operon was proposed by utilizing ABC importers from R. cellulolyticum. SBP is a key determinant of the substrate specificity and high-affinity of translocators, which tend to be in stoichiometric excess compared to the ABC transporter (31). It is achieved by the introduction of an internal intrinsic (Rho-independent) terminator into the downstream of SBP genes of operons encoding ABC importers, which results in the premature termination of transcription, and the stoichiometric ratio of genes flanking terminators is precisely determined by their TE at the transcriptional level. In addition to Gram-positive cellulolytic Clostridia, the strategy was also found in ABC importers from E. coli. Fifty ABC importers have been identified or predicted in E. coli K-12 serotype (32), including the well-studied MalFGK2-E (maltose) (33), BtuCD-F (vitamin B12) (34), and ModBC-A (Molybdate) (35). We found that at least 15 stem-loops were located downstream of the 5′-end of SBP genes in E. coli (Table S3) by predicting RNA secondary structure, suggesting that this same mechanism works in E. coli. This strategy was also mentioned in the recent report (36). It found that many bacterial gene clusters encoding conserved pathways have undergone massive divergence in transcript abundance and architectures via remodeling of internal terminators. Thus, it is believed that the mechanism found in the operons encoding ABC importers is widespread in prokaryotic cells.

Internal intrinsic terminators are mostly found in attenuators, such as the *trp* attenuator (37) and various riboswitches (38, 39), where the efficiency of these terminators is controlled by a regulatory effector that can be a metabolite, protein, or RNA (40). Compared with complex secondary structures of conditional transcription termination in attenuation, only single stem-loop structures without any additional structures, such as anti-terminator, were found in the gene clusters encoding ABC importers from cellulolytic Clostridia. Furthermore, our previous study revealed that transcription of gene clusters encoding ABC transporters is controlled by two-component systems (TCS) in R. cellulolyticum (41), which respond to the availability of extracellular soluble substrates, such as various sugars, resulting in that there was a significant difference in the transcription level of ABC transporters under various carbon sources (glucose, cellobiose, and cellulose), but the transcriptional ratio of SBP genes to their cognate translocator genes almost remained constant (Fig. 2). Thus, it is suggested that the internal termination of ABC importers occurs throughout transcription and cannot be affected by the environment. In total, internal intrinsic terminators, employed to control the differential expression between SBP and its cognate translocator by Clostridia, are more convenient than the introduction of multiple promoters because they are single elements and have a small number of bases. On the other hand, stoichiometry controlled by internal intrinsic terminators only depends on their sequences and structure and is evitably coupled with cellular environments in the hosts.

Essential elements of an intrinsic terminator consist of a GC-rich dyad repeat that forms a stem-loop structure, followed by a T-rich stretch, which generates a U-rich tail in the RNA after termination (42). The T-rich stretch is highly conserved between terminators in bacteria, while the sequences of the stem-loop seem to not be conserved, except for their GC-rich characteristic (43). Here, we further determined the elements impacting the TE of intrinsic terminators by comparing the internal terminators from operons encoding ABC importers. First, all stem-loop types, except Type I, harbor a series of 3′ nonconserved sequences with discrete U residues, and of these, the U number was lower than that of canonical terminators (44), resulting in transcripts with various TEs correlated with the length of their poly(U) tracts which is consistent with findings in B. subtilis (24). It has been confirmed that the role of a T stretch could be in slowing the elongation complex (EC) down at the termination point, thus giving the stem-loop extra time to be formed (45, 46). Second, canonical terminators prefer to have a ≥7-bp GC-rich stem that causes the RNA-DNA hybrid to melt, contributing to the destabilization of the EC (26, 46, 47). Thus, lengthening or shortening the stem beyond these limits decreased (or abolished) termination activity (48). However, the length of stems in this study was longer than that of canonical terminators from E. coli. Among the various lengths (5, 7, 9, 11, and 13 bp) of GC-rich stems from SL-2112 and its derivatives, an 11 bp lengthened stem had the maximum TE of 98.1 ± 0.3%, while a 5 bp shortened stem hardly had any termination activity. Third, although the extended A-U pairing between poly(A) and the U-tract flanking the stem-loop is generally considered to be associated with bidirectional termination (48) and not involved in termination function (24–26), the pairing of the 3′ U-tract with 5′ sequences in many stem-loops of Type II and Type IV is crucial to the termination function because their termination activity was abolished when the 5′ sequences paired with 3′ U-tract were deleted. Finally, Type IV stem-loops harbor an unpaired region in the middle of their stems but not distinct U-rich tracts; so, the 3′ sequences following unpaired regions were considered to be their putative U-rich tracts like Type III stem-loops. Although these sequences as U-rich tracts had termination activity, the length of the stems upstream of these putative U-rich tracts was 6 to 7 bp shorter than that of Type III stem-loops. These showed termination activity and insufficient termination of transcription, suggesting that the real U-rich tracts for Type IV stem-loops may have been located at their bottoms. Therefore, the lower U content of U-rich tracts, longer GC-rich stems, and pairing of 3′ U-tract was found in the internal terminators from operons encoding ABC importers, demonstrating their roles in controlling the stoichiometric ratio of their flanking transcripts and differences in RNA polymerases in structure between E. coli and R. cellulolyticum.

It is well-known that the terminal stem-loops of transcripts can confer resistance to exonuclease degradation, and the degree of protection is related to the folding energy ΔG of the stem-loop (49, 50). Given the strong 3′ to 5′ exonuclease activity, the ΔG of the RNA secondary structure at the 3′ end of a transcript is much lower than that of an RNA secondary structure at the 5′ end. In this study, all stem-loops, except SL-0885, also harbored 3′ U-rich tracts paired with 5′ sequences to form longer stems with lower ΔG. We hypothesized that they would result in the higher stability of their upstream transcripts. On the contrary, we found that unpaired regions introduced into Type III and IV stem-loops caused the ΔG to increase, resulting in the lower transcript abundance of their upstream genes. Thus, increasing or decreasing the ΔG of stem-loops could change the stability of these transcripts, consequentially controlling the stoichiometric ratio of their flanking transcripts.

In this study, we found that operons encoding ABC importers tended to harbor internal terminators downstream of SBP genes, which allowed the premature termination of transcription, resulting in an increased abundance of SBPs relative to their cognate translocators. However, internal terminators had a lower U content in their U-rich tracts and longer GC-rich stems, which distinguish them from that of canonical terminators and potentially endow them with special TEs. In addition to U-rich tracts and GC-rich stems, the paired regions of U-rich tracts and the unpaired regions of internal terminators contributed to the stability of their upstream transcripts. Altogether, internal terminators could effectively control the stoichiometric ratio of flanking transcripts based on both the TE and stability of transcripts determined by their sequences. Thus, the internal terminator shows promise for development as a synthetic biology tool to control the differential expression of genes in an operon.

## MATERIALS AND METHODS

### Strains and culture conditions.

The bacterial strains used in this study are listed in Table S4. Escherichia coli DH5α was used as the host strain for routine cloning and was incubated at 37°C in Luria-Bertani (LB) medium. R. cellulolyticum H10 (ATCC 35319) was cultured anaerobically in Hungate tubes at 35°C in GS-2 medium (K_2_HPO_4_ 2.9 g/liter, KH_2_PO_4_ 1.5 g/liter, urea 2.1 g/liter, resazurin 1.0 mg/liter, yeast extract 6.0 g/liter, cysteine-HCl 0.5 g/liter, MOPS 10.0 g/liter, and Trisodium citrate 3.0 g/liter, pH 7.4) supplemented with 3.0 g/liter cellobiose as the sole carbon source (default carbon source unless otherwise stated). When required, the media for E. coli and R. cellulolyticum were supplemented with 100 μg/mL ampicillin or 20 μg/mL erythromycin, respectively.

### Plasmid construction.

All plasmids constructed in this study are listed in Table S4. To analyze the function of various stem-loop structures, a dual fluorescence reporter system was first constructed by inserting the *mcherry* gene (encoding a red fluorescence protein) after *the fbfp* gene (encoding an anaerobic green fluorescence protein) into pMTC6, which is an R. cellulolyticum–E. coli shuttle vector (30). In the resulting plasmid pMTC9, *fbfp* and *mcherry* were expressed in a single operon promoted by the promoter *Pthl* (the thiolase gene promoter from Clostridium acetobutylicum) (51), with a *Bgl* II restriction site between the two genes for the introduction of various stem-loop structures. The stem-loops were fused with mcherry by single overlap extension PCR (SOE-PCR) using synthetic oligonucleotide primers (Table S5). The fused fragments were subsequently digested with the restriction enzymes *Bgl* II and EcoR I and then ligated into the shuttle vector pMTC9, which had been previously digested with the same enzymes.

pSY6-2112 for the targeted disruption of Ccel_2112 was constructed based on the targetron plasmid pSY6 (52) (Table S4). The targeting site for disruption and the intron retargeting primers were designed with an online tool based on the Perutka algorithm (http://clostron.com/) (22). The targeting region was obtained by SOE-PCR with the primer set Ccel_2112-IBS/EBS universal primer and Ccel_2112-EBS2/Ccel_2112-EBS1d successively, based on previous reports (52) (Table S5). The targeting region was subsequently digested with *Xho* I and *Bsr*G I and cloned between the corresponding sites of pSY6 to generate pSY6-2112. These plasmids were transformed into R. cellulolytiucm H10Δ*mspI* based on our previous electroporation method (53). All the transformants were then isolated on GS-2 solid medium containing 20 μg/mL erythromycin.

### Prediction of gene clusters encoding ABC importers.

To predict genes encoding ABC importers in cellulolytic Clostridia, we subjected all types of ABC importers annotated in the Kyoto Encyclopedia of Genes and Genomes (KEGG) database (http://www.genome.ad.jp/kegg/) to a blast search with eight sequenced genomes of cellulolytic Clostridia, R. cellulolyticum H10, R. papyrosolvens DSM2782, *R.* sp. BNL1100, R. josui JCM17888, R. termitidis CT1112, *R.* sp. MA18, R. sufflavum DSM 19573, and C. cellulovorans 743B, from the NCBI database (https://www.ncbi.nlm.nih.gov/). The BLAST threshold of the E value for statistical significance was set to 1×10^−5^.

### RNA secondary structure prediction.

On-line Mfold (http://unafold.rna.albany.edu/) was used to predict the RNA secondary structures of the intergenic regions between SBPs and their downstream genes using suggested default parameters (54). The output secondary structures were collected and analyzed for the presence of stems and loops. Stem-loop structures with a minimum of folding free energy (ΔG) were used to compare and classify these stem-loops.

### Promoter prediction.

The putative promoters in the intergenic regions (IRs, more than 50 bp) between SBP genes and their cognate translocator genes were predicted by using BDGP prokaryotic promoter prediction program (https://www.fruitfly.org) (55). The probability of occurrence of promoter sequences was determined using Promoter prediction (P) score, which can have values from 0 to 1 (a value close to 1 means high probability) (56). It is considered a putative promoter when its score was more than 0.9 in this study.

### Analysis of promoter activity.

The predicted promoters of P0044 and P0048 were amplified by PCR using synthetic primers (Table S4). The PCR products of P0044 and P0048 were purified and digested with restriction enzymes *Pst* I and *Mlu* I. The digested fragments were then ligated to pMTC6 which was digested with the same enzymes, resulting in the replacement of the original *Pthl* promoter (promoter for *fbfp* gene in pMTC6) by P0044 and P0048. The plasmids were transformed into R. cellulolyticum H10Δ*mspI*. The transformants of R. cellulolyticum were used to analyze the activity of promoters by measuring the fluorescence intensity of FbFP based on our previous method (57).

### Northern blotting.

Total RNA was isolated from R. cellulolyticum cultures on cellobiose in mid-log-phase, and genomic DNA was removed using RNase-Free DNase Set (Sangon, China). The RNA quality was determined using a NanoVue Plus spectrophotometer (Biochrom, UK), and electrophoresis on 1% agarose-formaldehyde gels was performed.

Two micrograms of total RNA were electrophoresed on 1% agarose-formaldehyde gels and blotted onto a positively charged nylon membrane (GE HealthCare, USA) using the NorthernMax kit (Life Technologies, USA). DIG-labeled DNA probes for the detection of specific transcripts were generated with a DIG labeling and detection kit (Roche, Switzerland), as per the manufacturer’s instructions, using the oligonucleotides listed in Table S5.

### Quantitative reverse transcription-PCR (qRT-PCR).

RNA was reverse transcribed using the HiScript III RT SuperMix kit (Vazyme, China). qRT-PCR was performed on a CFX96 real-time PCR detection system (Bio-Rad, USA) using ChamQ Univeral SYBR qPCR Master Mix (Vazyme, China). Data were normalized against the abundance of Ccel_0312 encoding the β subunit of DNA-directed RNA polymerase. The primer sets for qPCR are listed in Table S5.
