# Supplementary material for: Internal Transcription Terminators Control Stoichiometry of ABC Transporters in Cellulolytic Clostridia
Source: Microbiol Spectr. 2022 Mar 14;10(2):e01656-21. doi: 10.1128/spectrum.01656-21 (PMC9045158; doi:10.1128/spectrum.01656-21)
Supplement: SUPPLEMENTAL FILE 1 — Supplemental material. Download SPECTRUM01656-21_Supp_1_seq12.pdf, PDF file, 0.3 MB [file spectrum01656-21_supp_1_seq12.pdf]

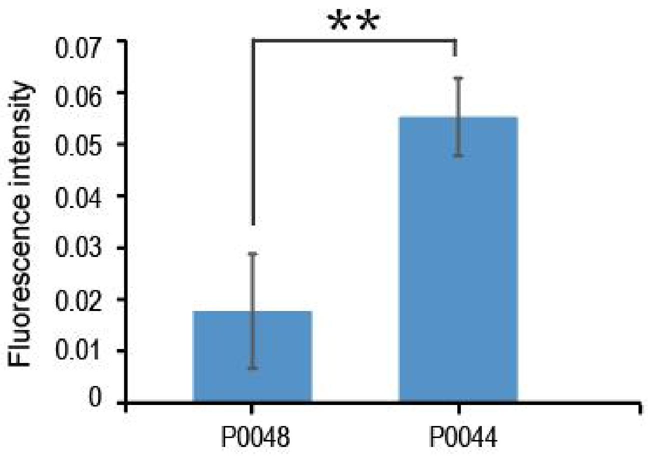

Figure S1. Promoter activity analysis of the gene cluster of Ccel_0044-0048. The promoter activity of Ccel_0044 and Ccel_0048 promoting expression of FbFP was respectively measured fluorometrically in *R. cellulolyticum* grown on cellobiose. Error bars indicate the standard deviation of the mean from experiments done in triplicate (***P value* < 0.01, Student's t-test).

Table S1 The ABC importer gene clusters from eight cellulolytic clostridia

Number	Replicon Accession	Start	End	Gene clusters encoding ABC importer	Gene number	Strand	SBP gene	Arrangement*	IRs between SBP and its cognate translocator gene				
									Length	Promoter prediction**		Stem-loop prediction***	
										Location	Score	Location	ΔG
Ruminiclostridium cellulolyticum H10													
1	NC_011898.1	46249	52121	Ccel_0044-Ccel_0048	5	-	Ccel_0044	TTNNS	185	136-181	0.99		
2	NC_011898.1	162607	166110	Ccel_0143-Ccel_0145	3	+	Ccel_0145	TTS	62			0-34	-11.50
3	NC_011898.1	172536	176220	Ccel_0150-Ccel_0152	3	+	Ccel_0150	STT	91			13-51	-25.50
4	NC_011898.1	224941	227254	Ccel_0200-Ccel_0202	3	+	Ccel_0200	STT	106			15-68	-25.50
5	NC_011898.1	498993	501503	Ccel_0435-Ccel_0437	3	-	Ccel_0437	STN	23				
6	NC_011898.1	1055703	1058312	Ccel_0878-Ccel_0880	3	-	Ccel_0878	NTS	38				
7	NC_011898.1	1062637	1065100	Ccel_0885-Ccel_0887	3	+	Ccel_0885	STN	186	73-118	0.99	43-69	-15.80
8	NC_011898.1	1065889	1069634	Ccel_0889-Ccel_0892	4	+	Ccel_0892	NTTS	1				
9	NC_011898.1	1126262	1129810	Ccel_0946-Ccel_0948	3	+	Ccel_0948	TTS	70			29-46	-10.80
10	NC_011898.1	1239924	1243099	Ccel_0998-Ccel_1000	3	+	Ccel_0998	STT	86			32-72	-25.00
11	NC_011898.1	1262279	1266034	Ccel_1014-Ccel_1016	3	+	Ccel_1016	TTS	80			7-54	-13.90
12	NC_011898.1	1277219	1280390	Ccel_1025-Ccel_1027	3	+	Ccel_1025	STT	92			26-62	-24.1
13	NC_011898.1	1329221	1334040	Ccel_1075-Ccel_1078	4	+	Ccel_1075	SNTT	33				
14	NC_011898.1	1393138	1396397	Ccel_1133-Ccel_1135	3	+	Ccel_1133	STT	165	99-144	0.92	32-80	-22.4
15	NC_011898.1	1497206	1501947	Ccel_1223-Ccel_1226	4	+	Ccel_1223	SNTT	143			27-70	-20.3
16	NC_011898.1	1552551	1556173	Ccel_1252-Ccel_1254	3	+	Ccel_1252	STT	105			18-56	-18.2
17	NC_011898.1	1708909	1713745	Ccel_1406-Ccel_1409	4	+	Ccel_1406	SNTT	124			36-86	-28.1
18	NC_011898.1	1938227	1939890	Ccel_1599-Ccel_1600	2	-	Ccel_1600	ST	131	59-104	0.93		
19	NC_011898.1	1966688	1969612	Ccel_1631-Ccel_1633	3	-	Ccel_1633	STT	-11				
20	NC_011898.1	1978780	1982300	Ccel_1642-Ccel_1644	3	-	Ccel_1642	NTS	55				
21	NC_011898.1	2111369	2117542	Ccel_1764-Ccel_1768	5	-	Ccel_1768	STTNN	93			22-56	-19.4
22	NC_011898.1	2350118	2354089	Ccel_1985-Ccel_1987	3	-	Ccel_1987	SNT	127			50-106	-33.2
23	NC_011898.1	2458887	2461785	Ccel_2101-Ccel_2103	3	-	Ccel_2101	TTS	144	97-142	0.97		
24	NC_011898.1	2470139	2473329	Ccel_2110-Ccel_2112	3	-	Ccel_2112	STT	106			26-63	-19.4
25	NC_011898.1	2764149	2768151	Ccel_2348-Ccel_2351	4	-	Ccel_2351	SNTT	108			55-83	-10.8
26	NC_011898.1	2969593	2973356	Ccel_2456-Ccel_2458	3	+	Ccel_2458	STT	97			18-48	-11.2
27	NC_011898.1	3121079	3123364	Ccel_2587-Ccel_2589	3	-	Ccel_2589	STN	98			25-63	-21.2
28	NC_011898.1	3207511	3211369	Ccel_2660-Ccel_2663	4	-	Ccel_2663	STTN	42				
29	NC_011898.1	3233890	3236594	Ccel_2686-Ccel_2687	2	-	Ccel_2687	SN	110			21-62	-21.7
30	NC_011898.1	3546061	3549310	Ccel_2995-Ccel_2997	3	-	Ccel_2997	STT	137			31-65	-22.00
31	NC_011898.1	3736498	3739783	Ccel_3202-Ccel_3204	3	-	Ccel_3204	STN	12				
32	NC_011898.1	3789864	3793085	Ccel_3244-Ccel_3246	3	-	Ccel_3246	STT	172	94-139	0.9	35-70	-20.00
Ruminiclostridium sp. BNL1100													
1	NC_016791.1	51999	57859	CLO1100_RS00250-CLO1100_RS00270	5	-	CLO1100_RS00250	TTNNS	194	140-185	1	136-166	
2	NC_016791.1	149315	152818	CLO1100_RS00690-CLO1100_RS00700	3	+	CLO1100_RS00700	TTS	62			0-34	-10.80
3	NC_016791.1	156458	159624	CLO1100_RS00715-CLO1100_RS00725	3	+	CLO1100_RS00715	STT	90			23-71	-23.40

4	NC_016791.1	165730	169418	CLO1100_RS00750-CLO1100_RS00760	3	+	CLO1100_RS00750	STT	86			8-53	-24.70
5	NC_016791.1	224402	227579	CLO1100_RS01030-CLO1100_RS01040	3	+	CLO1100_RS01030	STT	122			26-77	-25.00
6	NC_016791.1	391157	394299	CLO1100_RS01700-CLO1100_RS01710	3	+	CLO1100_RS01700	STT	67			13-44	-20.80
7	NC_016791.1	683048	685333	CLO1100_RS02995-CLO1100_RS03005	3	+	CLO1100_RS02995	STN	98			29-59	-16.70
8	NC_016791.1	848017	851786	CLO1100_RS03690-CLO1100_RS03700	3	-	CLO1100_RS03690	TTS	96			33-62	
9	NC_016791.1	1063482	1069795	CLO1100_RS04395-CLO1100_RS04410	4	+	CLO1100_RS04395	SNTT	118			26-73	-29.20
10	NC_016791.1	1120324	1123971	CLO1100_RS04555-CLO1100_RS04565	3	+	CLO1100_RS04555	STT	130	69-114	1	17-57	-21.10
11	NC_016791.1	1264817	1268121	CLO1100_RS05245-CLO1100_RS05255	3	+	CLO1100_RS05245	STT	255			202-240	-18.6
12	NC_016791.1	1311472	1316309	CLO1100_RS05425-CLO1100_RS05440	4	+	CLO1100_RS05425	SNTT	123			34-84	-27.30
13	NC_016791.1	1666449	1668954	CLO1100_RS06890-CLO1100_RS06900	3	-	CLO1100_RS06900	STN	15				
14	NC_016791.1	2130299	2135557	CLO1100_RS08865-CLO1100_RS08885	5	+	CLO1100_RS08865	STTNN	-2				
15	NC_016791.1	2251690	2255210	CLO1100_RS09340-CLO1100_RS09350	3	-	CLO1100_RS09340	NTS	58	7-52	1		
16	NC_016791.1	2266525	2270440	CLO1100_RS09400-CLO1100_RS09415	4	-	CLO1100_RS09415	STTN	21				
17	NC_016791.1	2411018	2417203	CLO1100_RS10060-CLO1100_RS10080	5	-	CLO1100_RS10080	STTNN	93			22-56	-20.00
18	NC_016791.1	2673784	2677768	CLO1100_RS11300-CLO1100_RS11310	3	-	CLO1100_RS11310	SNT	146			67-126	-34.90
19	NC_016791.1	2846192	2849382	CLO1100_RS12140-CLO1100_RS12150	3	-	CLO1100_RS12150	STT	106			25-65	-22.00
20	NC_016791.1	2944341	2946947	CLO1100_RS12515-CLO1100_RS12525	3	-	CLO1100_RS12525	STN	55				
21	NC_016791.1	2971804	2975413	CLO1100_RS12640-CLO1100_RS12650	3	-	CLO1100_RS12640	NTS	84	24-69	0.99		
22	NC_016791.1	3233681	3236853	CLO1100_RS13605-CLO1100_RS13615	3	-	CLO1100_RS13615	STT	101			17-49	-23.80
23	NC_016791.1	3370505	3373762	CLO1100_RS14205-CLO1100_RS14215	3	-	CLO1100_RS14215	STT	159			28-78	-23.80
24	NC_016791.1	3437212	3440127	CLO1100_RS14505-CLO1100_RS14515	3	+	CLO1100_RS14515	TNS	11				
25	NC_016791.1	3447154	3451986	CLO1100_RS14560-CLO1100_RS14575	4	-	CLO1100_RS14575	SNTT	32				
26	NC_016791.1	3454562	3459294	CLO1100_RS14590-CLO1100_RS14605	4	-	CLO1100_RS14605	SNTT	72			16-34	-11.30
27	NC_016791.1	3466581	3469855	CLO1100_RS14645-CLO1100_RS14655	3	-	CLO1100_RS14645	TTS	69				
28	NC_016791.1	3502746	3505965	CLO1100_RS14830-CLO1100_RS14840	3	-	CLO1100_RS14840	STT	97			10-43	-22.70
29	NC_016791.1	3514211	3517458	CLO1100_RS14890-CLO1100_RS14900	3	-	CLO1100_RS14900	STT	86			30-58	-19.90
30	NC_016791.1	3568932	3574590	CLO1100_RS15125-CLO1100_RS15140	4	-	CLO1100_RS15140	STTN	148	69-114	0.99	32-63	-19.40
31	NC_016791.1	3639012	3642754	CLO1100_RS15405-CLO1100_RS15420	4	-	CLO1100_RS15405	NTTS	1				
32	NC_016791.1	3643542	3646050	CLO1100_RS15430-CLO1100_RS15440	3	-	CLO1100_RS15440	STN	160	78-123	0.94	51-77	-16.60
33	NC_016791.1	3649075	3651684	CLO1100_RS15460-CLO1100_RS15470	3	+	CLO1100_RS15470	NTS	38				
34	NC_016791.1	3651867	3654378	CLO1100_RS15475-CLO1100_RS15485	3	-	CLO1100_RS15475	NTS	29				
35	NC_016791.1	3714583	3718131	CLO1100_RS15745-CLO1100_RS15755	3	-	CLO1100_RS15745	TTS	71			29-48	-11.70
36	NC_016791.1	4223597	4226840	CLO1100_RS18125-CLO1100_RS18135	3	-	CLO1100_RS18135	STT	131			38-80	-23.50
37	NC_016791.1	4322683	4325971	CLO1100_RS18630-CLO1100_RS18640	3	-	CLO1100_RS18640	STN	12				
38	NC_016791.1	4516260	4518851	CLO1100_RS19485-CLO1100_RS19495	3	-	CLO1100_RS19495	SNT	28				
Ruminiclostridium papyrosolvens DSM2782													
1	NZ_ACXX02000001.1	144170	147447	CPAP_RS00420-CPAP_RS00430	3	+	CPAP_RS00430	TTS	72	20-65	0.96		
2	NZ_ACXX02000001.1	156821	161647	CPAP_RS00480-CPAP_RS00495	4	+	CPAP_RS00480	SNTT	32				
3	NZ_ACXX02000001.1	195733	198587	CPAP_RS00685-CPAP_RS00695	3	-	CPAP_RS00685	TNS	10				
4	NZ_ACXX02000001.1	281135	285787	CPAP_RS01065-CPAP_RS01075	3	+	CPAP_RS01065	STT	158			28-78	-24.30
5	NZ_ACXX02000001.1	476329	479515	CPAP_RS01905-CPAP_RS01915	3	+	CPAP_RS01905	STT	115			32-63	-23.70
6	NZ_ACXX02000002.1	27493	32234	CPAP_RS02915-CPAP_RS02930	4	+	CPAP_RS02915	SNTT	146			27-69	-20.60

7	NZ_ACXX02000002.1	79786	83433	CPAP_RS03065-CPAP_RS03075	3	+	CPAP_RS03065	STT	131	77-122	0.95	16-63	-21.00
8	NZ_ACXX02000002.1	221118	226150	CPAP_RS03775-CPAP_RS03795	5	+	CPAP_RS03775	STTNN	134				
9	NZ_ACXX02000002.1	243308	246290	CPAP_RS03875-CPAP_RS03885	3	+	CPAP_RS03875	STN	3				
10	NZ_ACXX02000002.1	261288	264452	CPAP_RS03965-CPAP_RS03975	3	+	CPAP_RS03965	STT	108			29-70	-11.50
11	NZ_ACXX02000002.1	316272	319440	CPAP_RS04210-CPAP_RS04220	3	+	CPAP_RS04210	STT	86			26-71	-19.80
12	NZ_ACXX02000002.1	364552	369379	CPAP_RS04410-CPAP_RS04425	4	+	CPAP_RS04410	SNTT	124			34-84	-27.60
13	NZ_ACXX02000003.1	20398	24390	CPAP_RS04990-CPAP_RS05000	3	+	CPAP_RS04990	SNT	145			68-126	-35.10
14	NZ_ACXX02000003.1	285112	291298	CPAP_RS06205-CPAP_RS06225	5	+	CPAP_RS06205	STTNN	94			23-57	-19.40
15	NZ_ACXX02000004.1	203347	206635	CPAP_RS07815-CPAP_RS07825	3	+	CPAP_RS07815	STN	12				
16	NZ_ACXX02000005.1	148167	150455	CPAP_RS08885-CPAP_RS08895	3	-	CPAP_RS08895	STN	93			26-48	-13.80
17	NZ_ACXX02000005.1	167447	171496	CPAP_RS08975-CPAP_RS08990	4	-	CPAP_RS08985	NSTN	12				
18	NZ_ACXX02000006.1	62149	67986	CPAP_RS09890-CPAP_RS09910	5	-	CPAP_RS09890	TTNNS	182	123-168	0.99		
19	NZ_ACXX02000006.1	167445	170947	CPAP_RS10360-CPAP_RS10370	3	+	CPAP_RS10370	TTS	61			0-34	-10.70
20	NZ_ACXX02000006.1	177177	180861	CPAP_RS10395-CPAP_RS10405	3	+	CPAP_RS10395	STT	85			12-46	-23.90
21	NZ_ACXX02000006.1	246863	250030	CPAP_RS10725-CPAP_RS10735	3	+	CPAP_RS10725	STT	112			16-67	-26.40
22	NZ_ACXX02000007.1	31377	35297	CPAP_RS10955-CPAP_RS10970	4	+	CPAP_RS10955	STTN	21				
23	NZ_ACXX02000007.1	47710	51225	CPAP_RS11025-CPAP_RS11035	3	+	CPAP_RS11035	NTS	56				
24	NZ_ACXX02000007.1	78055	80294	CPAP_RS21315-CPAP_RS11150	3	+	CPAP_RS21315	STT	78			16-52	-16.50
25	NZ_ACXX02000007.1	85773	88537	CPAP_RS11190-pstA	3	+	CPAP_RS11190	STN	16				
26	NZ_ACXX02000010.1	127400	130580	CPAP_RS14495-CPAP_RS14505	3	+	CPAP_RS14495	STT	122			11-56	-24.70
27	NZ_ACXX02000010.1	178649	181160	CPAP_RS14680-CPAP_RS14690	3	+	CPAP_RS14690	NTS	30				
28	NZ_ACXX02000011.1	100208	102800	CPAP_RS15315-CPAP_RS15325	3	-	CPAP_RS15325	SNT	29				
29	NZ_ACXX02000012.1	74795	77280	CPAP_RS16020-CPAP_RS16030	3	-	CPAP_RS16030	STN					
30	NZ_ACXX02000013.1	41270	45176	CPAP_RS16530-CPAP_RS21395	3	+	CPAP_RS16530	STT	144			90-123	-16.70
31	NZ_ACXX02000013.1	63972	67741	CPAP_RS16615-CPAP_RS16625	3	-	CPAP_RS16615	TTS	96			21-47	-11.90
32	NZ_ACXX02000015.1	73714	77321	CPAP_RS17945-CPAP_RS17955	3	+	CPAP_RS17955	NTS	82	25-70	0.97		
33	NZ_ACXX02000015.1	93643	96251	modA-PAP_RS18040	3	+	modA	STTN	57			7-39	-15.30
34	NZ_ACXX02000016.1	90955	94123	CPAP_RS18615-CPAP_RS18625	3	+	CPAP_RS18615	STT	103			21-53	-13.20
35	NZ_ACXX02000017.1	100974	104164	CPAP_RS19285-CPAP_RS19295	3	-	CPAP_RS19295	STT	106			25-65	-22.00
36	NZ_ACXX02000018.1	6984	9499	CPAP_RS19385-CPAP_RS19395	3	+	CPAP_RS19385	STN	207	118-163	0.99	37-83	-20.80
37	NZ_ACXX02000018.1	10287	14029	CPAP_RS19405-CPAP_RS19420	4	+	CPAP_RS19420	NTTS	1				
38	NZ_ACXX02000018.1	55629	59986	CPAP_RS19580-CPAP_RS19595	4	+	CPAP_RS19580	STTN	119			37-93	-17.60
Ruminiclostridium josui JCM17888													
1	NZ_JAGE01000001.1	127767	131756	K412_RS0100595-K412_RS0100605	3	-	K412_RS0100605	SNT	145			68-126	-35.40
2	NZ_JAGE01000001.1	277826	281016	K412_RS0101360-K412_RS0101370	3	-	K412_RS0101370	STT	106			25-65	-22.00
3	NZ_JAGE01000001.1	373611	376506	K412_RS0101760-K412_RS0101775	3	-	K412_RS0101775	STN	338			174-196	-10.00
4	NZ_JAGE01000001.1	403816	407419	K412_RS0101900-K412_RS0101910	3	-	K412_RS0101900	NTS	78	25-70	0.99		
5	NZ_JAGE01000001.1	571136	574783	K412_RS0102900-K412_RS0102910	3	-	K412_RS0102910	STT	134	72-117	0.9	20-68	-16.30
6	NZ_JAGE01000001.1	623188	627925	K412_RS0103050-K412_RS0103065	4	-	K412_RS0103065	SNTT	142			25-67	-20.60
7	NZ_JAGE01000001.1	752231	755996	K412_RS0103585-K412_RS0103595	3	+	K412_RS0103595	TTS	92				
8	NZ_JAGE01000001.1	918605	920889	K412_RS0104305-K412_RS0104315	3	-	K412_RS0104315	STN	97			21-56	-20.10
9	NZ_JAGE01000001.1	951171	953917	K412_RS0104425-K412_RS0104435	3	-	K412_RS0104435	SNT	10				

10	NZ_JAGE01000001.1	1332018	1334435	K412_RS0106255-K412_RS0106265	3	-	K412_RS0106265	STN	101			7-44	-20.40
11	NZ_JAGE01000001.1	1473306	1476585	K412_RS0107005-K412_RS0107015	3	-	K412_RS0107015	STN	12				
12	NZ_JAGE01000001.1	1851584	1857449	K412_RS0108675-K412_RS0108695	5	-	K412_RS0108675	TTNNS	177	124-169	0.96		
13	NZ_JAGE01000001.1	1951590	1955096	K412_RS0109135-K412_RS0109145	3	+	K412_RS0109145	TTS	62			0-34	-11.20
14	NZ_JAGE01000001.1	1961356	1965041	K412_RS0109175-K412_RS0109185	3	+	K412_RS0109175	STT	86			13-58	-24.70
15	NZ_JAGE01000001.1	2017619	2020776	K412_RS0109450-K412_RS0109460	3	+	K412_RS0109450	STT	96			16-66	-23.40
16	NZ_JAGE01000001.1	2439455	2441973	K412_RS0111205-K412_RS0111215	3	+	K412_RS0111215	NTS	37				
17	NZ_JAGE01000001.1	2442313	2444922	K412_RS0111225-K412_RS0111235	3	-	K412_RS0111225	NTS	38				
18	NZ_JAGE01000001.1	2447341	2449844	K412_RS0111250-K412_RS0111260	3	+	K412_RS0111250	SNT	208	115-160	0.99	43-71	-15.50
19	NZ_JAGE01000001.1	2450611	2454353	K412_RS0111270-K412_RS0111285	4	+	K412_RS0111285	NTTS	1				
20	NZ_JAGE01000001.1	2601653	2606377	K412_RS0111945-K412_RS0111960	4	+	K412_RS0111945	SNTT	64			10-27	-13.20
21	NZ_JAGE01000001.1	2607597	2612429	K412_RS0111970-K412_RS0111985	4	+	K412_RS0111970	SNTT	33				
22	NZ_JAGE01000001.1	2672583	2675837	K412_RS0112290-K412_RS0112300	3	+	K412_RS0112290	STT	160	81-126	0.96	30-79	-24.10
23	NZ_JAGE01000001.1	3032707	3037529	K412_RS0113650-K412_RS0113665	4	+	K412_RS0113650	SNTT	123			34-84	-27.30
24	NZ_JAGE01000001.1	3348795	3351276	K412_RS0115190-K412_RS0115200	3	-	K412_RS0115200	STN	15				
25	NZ_JAGE01000002.1	227064	233256	K412_RS0117615-K412_RS0117635	5	+	K412_RS0117615	STTNN	97			23-60	-25.50
26	NZ_JAGE01000002.1	433447	437392	K412_RS0118375-K412_RS0118390	4	+	K412_RS0118375	STTN	21				
27	NZ_JAGE01000002.1	497562	501025	K412_RS0118655-K412_RS0118670	4	+	K412_RS0118670	NTTS	58				
28	NZ_JAGE01000002.1	591955	594907	K412_RS0118900-K412_RS0118915	4	-	K412_RS0118900	TTNS	20				
29	NZ_JAGE01000002.1	697007	699416	K412_RS0119340-K412_RS0119350	3	-	K412_RS0119340	NTS	68	2-47	0.98		
Ruminiclostridium cellobioparum subsp. termitidis CT1112													
1	NZ_AORV01000002.1	7275	10429	CTER_RS00125-CTER_RS00135	3	-	CTER_RS00135	STT	93			27-66	-17.60
2	NZ_AORV01000002.1	34652	36983	CTER_RS00215-CTER_RS00225	3	-	CTER_RS00225	STN	141	74-119	1	21-72	-22.00
3	NZ_AORV01000004.1	14510	17603	CTER_RS00635-pstC	3	-	CTER_RS00635	TTS	287	198-243	0.98		
4	NZ_AORV01000007.1	3671	7328	CTER_RS00840-CTER_RS00850	3	-	CTER_RS00850	SNT	69			13-49	-21.40
5	NZ_AORV01000007.1	33318	38726	CTER_RS00930-CTER_RS00940	3	-	CTER_RS00930	TTS	72	19-64	0.96		
6	NZ_AORV01000008.1	40048	44963	CTER_RS01275-CTER_RS01290	4	-	CTER_RS01290	SNTT	258			30-91	-23.80
7	NZ_AORV01000009.1	2823	6728	CTER_RS01315-CTER_RS01325	3	-	CTER_RS01325	STT	446			344-373	-20.70
8	NZ_AORV01000009.1	18417	20851	CTER_RS01375-CTER_RS01385	3	-	CTER_RS01375	NTS	70	9-54	0.99		
9	NZ_AORV01000009.1	24480	27757	CTER_RS01405-CTER_RS01415	3	-	CTER_RS01415	STT	136	86-131	1	17-57	-18.10
10	NZ_AORV01000014.1	28547	31732	CTER_RS02285-CTER_RS02295	3	+	CTER_RS02285	STT	130			55-106	-18.20
11	NZ_AORV01000015.0	21179	25053	CTER_RS02395-CTER_RS02405	3	-	CTER_RS02405	SNT	74			7-32	-12.80
12	NZ_AORV01000015.0	73170	76661	CTER_RS02615-CTER-RS02625	3	-	CTER_RS02615	TTS	49				
13	NZ_AORV01000015.1	97335	100866	CTER_RS02685-CTER_RS02695	3	-	CTER_RS02685	TTS	200	39-84	0.92		
14	NZ_AORV01000015.1	108797	112532	CTER_RS02730-CTER_RS02740	3	-	CTER_RS02740	SNT	129			31-106	-32.80
15	NZ_AORV01000016.1	60834	64014	CTER_RS03015-CTER_RS03025	3	-	CTER_RS03015	TTS	104	22-67	0.91	60-94	-10.00
16	NZ_AORV01000016.1	83849	86709	pstA-CTER_RS03150	3	-	CTER_RS03150	STT	102			24-68	-20.50
17	NZ_AORV01000017.1	476	3870	CTER_RS03180-CTER_RS03190	3	+	CTER_RS03190	TTS	64	11-56	0.96		
18	NZ_AORV01000017.1	22875	26579	CTER_RS03290-CTER_RS03300	3	-	CTER_RS03290	NTS	67	15-60	1		
19	NZ_AORV01000017.1	45775	49230	CTER_RS03360-CTER_RS03370	3	-	CTER_RS03360	TTS	53				
20	NZ_AORV01000017.1	58308	61541	CTER_RS03405-CTER_RS03415	3	-	CTER_RS03405	TTS	146	82-127	0.98	19-66	-14.50
21	NZ_AORV01000017.1	66637	69911	CTER_RS03440-CTER-RS03450	3	-	CTER_RS03450	STT	69			17-46	-21.80

22	NZ_AORV01000018.1	27482	30679	CTER_RS03745-CTER_RS03755	3	-	CTER_RS03755	STT	90	17-62	0.94	37-69	-15.80
23	NZ_AORV01000020.1	28307	31229	CTER_RS04215-CTER_RS04225	3	-	CTER_RS04225	SNT	59	10-55	0.91		
24	NZ_AORV01000020.1	37646	40818	CTER_RS04260-CTER_RS04270	3	-	CTER_RS04270	STT	76			15-56	-32.40
25	NZ_AORV01000020.1	48144	51700	CTER_RS04305-CTER_RS04315	3	-	CTER_RS04315	SNT	45				
26	NZ_AORV01000020.1	73157	76805	CTER_RS04425-CTER_RS04435	3	-	CTER_RS04425	TTS	118	72-117	0.97		
27	NZ_AORV01000021.1	112118	117898	CTER_RS05005-CTER_RS05025	5	-	CTER_RS05005	TTNNS	182	126-171	0.98		
28	NZ_AORV01000021.1	118665	124903	CTER_RS05035-CTER_RS05055	5	-	CTER_RS05055	STTNN	110			42-81	-20.90
29	NZ_AORV01000021.1	125401	131062	CTER_RS05060-CTER_RS05080	5	-	CTER_RS05060	NNTTS	28				
30	NZ_AORV01000021.1	212544	217549	CTER_RS05440-CTER_RS05460	5	-	CTER_RS05440	TTNNS	40				
31	NZ_AORV01000022.1	8441	12357	CTER_RS05770-CTER_RS05780	3	+	CTER_RS05770	SNT	97			16-57	-25.40
32	NZ_AORV01000022.1	53013	58395	CTER_RS05940-CTER_RS05955	3	-	CTER_RS05940	STT	56				
33	NZ_AORV01000022.1	86328	89823	CTER_RS06065-CTER_RS06075	3	-	CTER_RS06065	TTS	139			75-107	-13.00
34	NZ_AORV01000022.1	90509	96363	CTER_RS06080-CTER_RS06100	5	+	CTER_RS06100	NNTTS	192	91-136	0.95		
35	NZ_AORV01000023.1	13482	16850	CTER_RS06430-CTER_RS06440	3	-	CTER_RS06440	STT	151	71-116	0.99	15-40	-21.30
36	NZ_AORV01000025.1	12046	15222	CTER_RS06625-CTER_RS06635	3	-	CTER_RS06635	STT	84			33-61	-17.40
37	NZ_AORV01000025.1	40473	43796	CTER_RS06730-CTER_RS06740	3	-	CTER_RS06740	STT	172	111-156	0.93	17-65	-24.00
38	NZ_AORV01000025.1	53443	56983	CTER_RS06785-CTER_RS06795	3	-	CTER_RS06785	TTS	49				
39	NZ_AORV01000025.1	95289	98452	CTER_RS06975-CTER_RS06985	3	-	CTER_RS06985	STT	70			11-45	-24.70
40	NZ_AORV01000025.1	129466	132833	CTER_RS07105-CTER_RS07115	3	-	CTER_RS07105	TTS	56				
41	NZ_AORV01000025.1	147828	151067	CTER_RS07165-CTER_RS07175	3	-	CTER_RS07175	STT	143			25-66	-24.40
42	NZ_AORV01000025.1	156294	159521	CTER_RS07200-CTER_RS07210	3	-	CTER_RS07210	STT	70			11-50	-24.80
43	NZ_AORV01000025.1	164713	170115	CTER_RS07235-CTER_RS07245	3	-	CTER_RS07235	TTS	52				
44	NZ_AORV01000025.1	185699	189355	CTER_RS07285-CTER_RS07295	3	-	CTER_RS07285	TTS	95			0-36	-15.50
45	NZ_AORV01000026.1	13700	16906	CTER_RS07390-CTER_RS07405	4	+	CTER_RS07405	TTNS	18				
46	NZ_AORV01000026.1	32844	36023	CTER_RS07480-CTER_RS07490	3	-	CTER_RS07490	STT	73			12-43	-21.70
47	NZ_AORV01000026.1	41575	44122	CTER_RS07515-CTER_RS07525	3	"-++"	CTER_RS07515	SNT	147	57-102	0.97	26-58	-4.80
48	NZ_AORV01000026.1	53712	56874	CTER_RS07550-CTER_RS07560	3	-	CTER_RS07560	STT	72			9-46	-17.20
49	NZ_AORV01000026.1	62333	65261	CTER_RS07595-CTER_RS07605	3	-	CTER_RS07605	STN	12				
50	NZ_AORV01000026.1	66240	70133	CTER_RS07615-CTER_RS07630	4	+	CTER_RS07615	STTN	-2				
51	NZ_AORV01000026.1	112802	116407	CTER_RS07775-CTER_RS07785	3	-	CTER_RS07775	NTS	41				
52	NZ_AORV01000026.1	139575	143137	CTER_RS07900-CTER_RS07910	3	-	CTER_RS07910	SNT	97			23-67	-28.30
53	NZ_AORV01000026.1	261795	264909	CTER_RS08390-CTER_RS08400	3	-	CTER_RS08400	STT	72			16-45	-15.10
54	NZ_AORV01000026.1	346244	349471	CTER_RS08655-CTER_RS08665	3	-	CTER_RS08665	STT	126			21-48	-20.10
55	NZ_AORV01000026.1	404154	409749	CTER_RS08870-CTER_RS08890	5	-	CTER_RS08890	STTNN	112			42-73	-22.60
56	NZ_AORV01000027.1	47419	51088	CTER_RS09110-CTER_RS09120	3	-	CTER_RS09110	TTS	100			35-71	-13.70
57	NZ_AORV01000027.1	65112	68443	CTER_RS09185-CTER_RS09195	3	-	CTER_RS09195	STT	453	359-404	0.98		
58	NZ_AORV01000027.1	82771	85857	CTER_RS09270-CTER_RS09280	3	-	CTER_RS09280	STT	59			10-47	-24.50
59	NZ_AORV01000027.1	104718	107993	CTER_RS09365-CTER_RS09375	3	-	CTER_RS09375	STT	199			86-113	-17.70
60	NZ_AORV01000027.1	114428	119256	CTER_RS09415-CTER_RS09430	4	-	CTER_RS09430	SNTT	34				
61	NZ_AORV01000028.1	330	3605	CTER_RS09475-CTER_RS09485	3	-	CTER_RS09485	STT	80			10-52	-24.20
62	NZ_AORV01000028.1	15985	21624	CTER_RS09535-CTER_RS09555	5	-	CTER_RS09555	STTNN	79			19-57	-26.80
63	NZ_AORV01000028.1	33152	35791	CTER_RS09610-CTER_RS09620	3	+	CTER_RS09610	STN	70			25-57	-17.60

64	NZ_AORV01000028.1	121203	126815	CTER_RS10035-CTER_RS10045	3	-	CTER_RS10045	STT	123			17-61	-17.30
65	NZ_AORV01000030.1	44861	47338	CTER_RS10535-CTER_RS10545	3	-	CTER_RS10535	TNS	-2				
66	NZ_AORV01000031.1	7791	11038	CTER_RS10660-CTER_RS10670	3	-	CTER_RS10670	STT	122			43-94	-28.90
67	NZ_AORV01000031.1	65439	68680	CTER_RS10895-CTER_RS10905	3	-	CTER_RS10905	STT	66			7-47	-25.70
68	NZ_AORV01000031.1	158439	161698	CTER_RS11240-CTER_RS11250	3	-	CTER_RS11250	STT	85			36-57	-15.90
69	NZ_AORV01000031.1	169454	172675	CTER_RS11275-CTER_RS11285	3	-	CTER_RS11285	STT	160	78-123	0.94	22-73	-26.00
70	NZ_AORV01000031.1	215622	218710	CTER_RS11515-CTER_RS11525	3	-	CTER_RS11515	TTS	48				
71	NZ_AORV01000033.1	53898	57085	CTER_RS12030-CTER_RS12040	3	-	CTER_RS12040	STT	20				
72	NZ_AORV01000033.1	119579	122313	CTER_RS12365-CTER_RS12375	3	-	CTER_RS12365	TNS	45				
73	NZ_AORV01000033.1	126310	129173	CTER_RS12400-CTER_RS12410	3	-	CTER_RS12410	SNT	59				
74	NZ_AORV01000033.1	131910	134797	CTER_RS12425-CTER_RS12435	3	-	CTER_RS12435	STN	120			74-99	-14.90
75	NZ_AORV01000033.1	175589	178823	CTER_RS12610-CTER_RS12620	3	-	CTER_RS12620	STT	132			22-92	-25.10
76	NZ_AORV01000035.1	37699	41322	CTER_RS12920-CTER_RS12930	3	-	CTER_RS12920	NTS	52				
77	NZ_AORV01000035.1	74985	78701	CTER_RS13035-CTER_RS13045	3	-	CTER_RS13045	STT	95			40-77	-23.00
78	NZ_AORV01000035.1	142164	145376	CTER_RS13300-CTER_RS13310	3	-	CTER_RS13310	STT	82			22-66	-30.50
79	NZ_AORV01000036.1	27951	31641	CTER_RS13665-CTER_RS13675	3	-	CTER_RS13675	STT	94			10-56	-23.30
80	NZ_AORV01000038.1	38783	41339	modA-CTER_RS14395	3	+	modA	STT	132	72-117	0.92	16-63	-23.40
81	NZ_AORV01000038.1	65727	68872	CTER_RS14530-CTER_RS14540	3	-	CTER_RS14540	STN	64				
82	NZ_AORV01000038.1	74903	77671	CTER_RS14560-CTER_RS14570	3	-	CTER_RS14570	STN	24				
83	NZ_AORV01000039.1	1495	4601	CTER_RS14610-CTER_RS14620	3	-	CTER_RS14620	STT	85			30-66	-19.50
84	NZ_AORV01000039.1	76100	80804	yjfF-CTER_RS14950	4	-	CTER_RS14950	SNTT	124			24-64	-22.40
85	NZ_AORV01000040.1	30972	34144	CTER_RS15085-CTER_RS15095	3	+	CTER_RS15095	TTS	78				
86	NZ_AORV01000040.1	72095	77831	CTER_RS15260-CTER_RS15280	5	-	CTER_RS15280	STTNN	155			99-135	-21.30
87	NZ_AORV01000042.1	17944	21632	CTER_RS15370-CTER_RS15380	3	-	CTER_RS15380	STT	102			27-89	-37.70
88	NZ_AORV01000042.1	70724	73855	CTER_RS15620-CTER_RS15630	3	-	CTER_RS15630	STT	80			13-58	-28.40
89	NZ_AORV01000042.1	79109	82312	CTER_RS15670-CTER_RS15680	3	-	CTER_RS15680	STT	73			20-54	-18.50
90	NZ_AORV01000043.1	28925	32150	CTER_RS15835-CTER_RS15845	3	-	CTER_RS15845	STT	98			9-56	-27.20
91	NZ_AORV01000043.1	54504	58588	CTER_RS15955-CTER_RS15970	4	-	CTER_RS15970	STTN	17				
92	NZ_AORV01000043.1	62967	65372	CTER_RS15995-CTER_RS16005	3	-	CTER_RS15995	NTS	60				
93	NZ_AORV01000043.1	76316	80985	CTER_RS16060-CTER_RS16080	5	-	CTER_RS16080	STTNN	160			80-112	-14.80
94	NZ_AORV01000044.1	39559	42893	CTER_RS16340-CTER_RS16350	3	-	CTER_RS16340	TTS	178	41-86	0.97		
95	NZ_AORV01000044.1	88136	91760	CTER_RS16575-CTER_RS16585	3	-	CTER_RS16575	TTS	86			27-53	-11.80
96	NZ_AORV01000045.1	6157	9768	CTER_RS16620-CTER_RS16630	3	-	CTER_RS16630	STT	123			21-62	-27.20
97	NZ_AORV01000045.1	28822	32310	CTER_RS16705-CTER_RS16715	3	-	CTER_RS16705	TTS	80				
98	NZ_AORV01000045.1	39398	43180	CTER_RS16740-CTER_RS16755	4	-	CTER_RS16740	NTTS	32				
99	NZ_AORV01000045.1	44061	46922	CTER_RS16765-CTER_RS16775	3	-	CTER_RS16775	STN	161	71-116	1	36-63	-20.50
100	NZ_AORV01000045.1	50073	52704	CTER_RS16795-CTER_RS16805	3	+	CTER_RS16805	NTS	54			21-39	-12.00
101	NZ_AORV01000045.1	55669	58215	CTER_RS16830-CTER_RS16840	3	-	CTER_RS16830	NTS	68	19-64	0.98		
102	NZ_AORV01000046.1	82606	85814	CTER_RS17275-CTER_RS17285	3	-	CTER_RS17285	STT	124			30-61	-19.40
103	NZ_AORV01000046.1	92702	95008	ehuA-CTER_RS17320	3	-	CTER_RS17320	STN	88			26-62	-19.90
104	NZ_AORV01000046.1	133750	137035	CTER_RS17495-CTER_RS17505	3	-	CTER_RS17505	STT	166			17-63	-27.10
105	NZ_AORV01000048.1	5500	8710	CTER_RS17775-CTER_RS17785	3	-	CTER_RS17785	STT	90			18-61	-26.10

106	NZ_AORV01000048.1	19078	22891	CTER_RS17825-CTER_RS17835	3	-	CTER_RS17835	SNT	65			11-46	-20.30
107	NZ_AORV01000049.1	23464	27132	CTER_RS17945-CTER_RS17955	3	+	CTER_RS17955	TTS	89	21-66	0.99		
108	NZ_AORV01000049.1	37372	40536	CTER_RS18000-CTER_RS18010	3	+	CTER_RS18000	STT	70			16-58	-22.80
109	NZ_AORV01000049.1	44929	49709	CTER_RS18030-CTER_RS18040	3	+	CTER_RS18030	STT	152			22-64	-21.00
110	NZ_AORV01000049.1	74506	78051	CTER_RS18135-CTER_RS18145	3	+	CTER_RS18135	STT	100			40-85	-11.60
111	NZ_AORV01000052.1	8372	12001	CTER_RS18485-CTER_RS18495	3	-	CTER_RS18495	STT	105			27-71	-25.00
112	NZ_AORV01000054.1	64663	68120	CTER_RS19070-CTER_RS19080	3	-	CTER_RS19070	TTS	57	9-54	0.97		
113	NZ_AORV01000057.1	14722	18109	CTER_RS19900-CTER_RS19910	3	-	CTER_RS19910	STT	115			11-75	-31.90
114	NZ_AORV01000058.1	53155	56512	CTER_RS20240-CTER_RS20250	3	-	CTER_RS20250	STT	154			68-106	-31.8
115	NZ_AORV01000058.1	62231	65508	CTER_RS20280-CTER_RS20290	3	-	CTER_RS20290	STT	93			30-72	-20.00
116	NZ_AORV01000058.1	99608	102757	CTER_RS20400-CTER_RS20410	3	-	CTER_RS20410	STT	88			35-73	-24.40
117	NZ_AORV01000058.1	118291	121445	CTER_RS20450-CTER_RS20460	3	-	CTER_RS20460	STT	91			22-55	-17.20
118	NZ_AORV01000058.1	121974	127199	CTER_RS20465-CTER_RS20475	3	-	CTER_RS20475	SNN	73	21-66	0.93		
119	NZ_AORV01000059.1	23180	26354	CTER_RS20610-CTER_RS20620	3	-	CTER_RS20620	STT	99			22-66	-20.80
120	NZ_AORV01000059.1	30402	33657	CTER_RS20640-CTER_RS20650	3	-	CTER_RS20650	STT	165			80-116	-18.80
121	NZ_AORV01000059.1	39560	43455	CTER_RS20680-CTER_RS20690	3	-	CTER_RS20690	SNT	244			138-201	-23.50
122	NZ_AORV01000060.1	7004	10701	CTER_RS20785-CTER_RS20795	3	-	CTER_RS20795	STT	182	102-147	1	35-76	-26.40
123	NZ_AORV01000060.1	73187	76337	CTER_RS21035-CTER_RS21045	3	-	CTER_RS21045	STT	92			25-67	-23.30
124	NZ_AORV01000060.1	92947	96439	CTER_RS21105-CTER_RS21115	3	-	CTER_RS21105	TTS	190	116-161	0.97		
125	NZ_AORV01000060.1	137835	141863	CTER_RS21300-CTER_RS21310	3	-	CTER_RS21310	SNT	196			61-97	-24.60
126	NZ_AORV01000060.1	148485	152388	CTER_RS21335-CTER_RS21345	3	-	CTER_RS21345	STT	253			206-240	-16.00
127	NZ_AORV01000061.1	81061	84181	CTER_RS21790-CTER_RS21800	3	-	CTER_RS21800	STT	103			22-47	-13.40
128	NZ_AORV01000065.1	4009	7522	CTER_RS22230-CTER_RS22240	3	-	CTER_RS22230	TTS	92	47-92	0.98		
129	NZ_AORV01000065.1	16435	19877	CTER_RS22270-CTER_RS22280	3	-	CTER_RS22270	TTS	65				
130	NZ_AORV01000065.1	63476	67100	CTER_RS22435-CTER_RS22445	3	-	CTER_RS22435	TTS	87	26-71	0.99		
131	NZ_AORV01000065.1	204451	208109	CTER_RS23130-CTER_RS23140	3	-	CTER_RS23130	NTS	133	37-82	0.9		
132	NZ_AORV01000065.1	282808	286270	CTER_RS23450-CTER_RS23460	3	-	CTER_RS23450	TTS	75	24-69	0.97		
133	NZ_AORV01000065.1	316286	319439	CTER_RS23560-CTER_RS23570	3	-	CTER_RS23570	STT	137			31-84	-25.30
134	NZ_AORV01000065.1	404168	407282	CTER_RS23955-CTER_RS23965	3	-	CTER_RS23965	STT	87			19-59	-23.10
135	NZ_AORV01000065.1	440189	443417	CTER_RS24130-CTER_RS24140	3	-	CTER_RS24140	STT	161			63-130	-32.80
136	NZ_AORV01000065.1	474257	477493	CTER_RS24275-CTER_RS24285	3	-	CTER_RS24285	STT	143			4-72	-36.30
137	NZ_AORV01000066.1	9726	13340	CTER_RS24325-CTER_RS24335	3	-	CTER_RS24325	TTS	73	10-55	1		
138	NZ_AORV01000066.1	53228	55960	CTER_RS24525-CTER_RS24535	3	-	CTER_RS24535	STN	28				
139	NZ_AORV01000066.1	60351	63921	CTER_RS24565-CTER_RS24575	3	-	CTER_RS24565	TTS	135	84-129	0.94		
140	NZ_AORV01000066.1	89829	93345	CTER_RS24690-CTER_RS24700	3	-	CTER_RS24690	TTS	54				
141	NZ_AORV01000066.1	121637	124510	CTER_RS24815-CTER_RS24825	3	-	CTER_RS24825	STT	84			27-57	-19.00
142	NZ_AORV01000068.1	18025	23088	CTER_RS24910-CTER_RS24925	4	-	CTER_RS24925	SNTT	206	112-157	0.99	38-68	-19.80
143	NZ_AORV01000068.1	50139	53382	CTER_RS25050-CTER_RS25065	4	-	CTER_RS25055	NTTS					
144	NZ_AORV01000070.1	42581	46160	CTER_RS25360-CTER_RS25370	3	-	CTER_RS25360	TTS	62				
145	NZ_AORV01000071.1	7255	10417	CTER_RS25455-CTER_RS25465	3	-	CTER_RS25465	STT	95			57-73	-16.90
146	NZ_AORV01000072.1	12742	16370	CTER_RS25510-CTER_RS25520	3	-	CTER_RS25520	STT	210	106-151	0.97		
147	NZ_AORV01000072.1	31609	35070	CTER_RS25585-CTER_RS25600	4	-	CTER_RS25585	STNT	22				

148	NZ_AORV01000072.1	64302	69405	urtE-urtA	5	-	urtA	TTS	58	10-55	0.93		
149	NZ_AORV01000072.1	95832	99460	CTER_RS25845-CTER_RS25855	3	-	CTER_RS25845	TTS	54				
150	NZ_AORV01000072.1	138717	142121	CTER_RS25980-CTER_RS25990	3	+	CTER_RS25990	STTNN	267	217-262	0.94		
151	NZ_AORV01000078.1	28515	31999	CTER_RS26190-CTER_RS26200	3	-	CTER_RS26190	TTS	64	12-57	0.97		
152	NZ_AORV01000078.1	57687	61194	CTER_RS26295-CTER_RS27340	3	-	CTER_RS27340	STT	320			22-79	-33.60
153	NZ_AORV01000078.1	65457	68827	CTER_RS26320-CTER_RS26330	3	-	CTER_RS26330	TTS	28				
154	NZ_AORV01000078.1	77941	81062	CTER_RS26375-CTER_RS26385	3	-	CTER_RS26375	SNT	150			40-90	-19.80
155	NZ_AORV01000078.1	106557	110526	CTER_RS26505-CTER_RS26515	3	-	CTER_RS26515	STN	55				
156	NZ_AORV01000078.1	117518	120825	CTER_RS26565-CTER_RS26575	3	-	CTER_RS26575	STT	69			11-55	-27.20
Ruminiclostridium sp.MA18													
1	NZ_RQSR01000001.1	378796	382862	EHE19_RS01495-EHE19_RS01510	4	+	EHE19_RS01495	STTN	61	5-50	0.96		
2	NZ_RQSR01000001.1	416747	420083	EHE19_RS01610-EHE19_RS01620	3	+	EHE19_RS01610	STT	265	134-179	1	47-89	-23.00
3	NZ_RQSR01000001.1	453354	456269	EHE19_RS01740-EHE19_RS01750	3	-	EHE19_RS01750	SNT	189	108-153	0.99		
4	NZ_RQSR01000001.1	513426	516284	EHE19_RS01930-EHE19_RS01940	3	-	EHE19_RS01940	SNT	28				
5	NZ_RQSR01000001.1	525378	530994	EHE19_RS01985-EHE19_RS01995	3	-	EHE19_RS01985	TNS	94			38-87	-15.00
6	NZ_RQSR01000001.1	534568	538183	EHE19_RS02030-EHE19_RS02040	3	-	EHE19_RS02030	NTS	87				
7	NZ_RQSR01000001.1	739991	743787	EHE19_RS02810-EHE19_RS02820	3	-	EHE19_RS02810	TTS	79	31-76	0.99		
8	NZ_RQSR01000001.1	881787	885306	EHE19_RS03425-EHE19_RS03435	3	-	EHE19_RS03425	TTS	57				
9	NZ_RQSR01000001.1	1093558	1097283	EHE19_RS04215-EHE19_RS04225	3	-	EHE19_RS04225	STT	648	517-562	0.99	211-281	-17.90
10	NZ_RQSR01000002.1	100192	103708	EHE19_RS04840-EHE19_RS04850	3	-	EHE19_RS04850	STT	441	341-386	0.98	191-239	-23.70
11	NZ_RQSR01000002.1	338668	343277	EHE19_RS05680-EHE19_RS05695	4	-	EHE19_RS05680	NTTS					
12	NZ_RQSR01000003.1	63236	69524	EHE19_RS06700-EHE19_RS06720	5	-	EHE19_RS06700	TTNNS	596	510-555	0.99	448-488	-11.70
13	NZ_RQSR01000003.1	73267	78127	EHE19_RS06740-EHE19_RS06755	4	-	EHE19_RS06755	SNTT	209	156-201	0.92	65-121	-30.80
14	NZ_RQSR01000003.1	135936	138828	EHE19_RS06995-EHE19_RS07005	3	+	EHE19_RS06995	STN	8				
15	NZ_RQSR01000003.1	240672	245089	EHE19_RS07440-EHE19_RS07450	3	+	EHE19_RS07440	STT	827	742-787	1		
16	NZ_RQSR01000003.1	312186	314982	EHE19_RS07745-EHE19_RS07755	3	+	EHE19_RS07745	STN	213	151-196	0.96		
17	NZ_RQSR01000006.1	34287	38038	EHE19_RS09980-EHE19_RS09990	3	+	EHE19_RS09980	STT	265	171-216	0.92		
18	NZ_RQSR01000006.1	72349	75855	EHE19_RS10105-EHE19_RS10115	3	+	EHE19_RS10115	TTS	70	1-46	0.97		
19	NZ_RQSR01000007.1	63005	66178	EHE19_RS10895-EHE19_RS10905	3	+	EHE19_RS10895	STT	114			37-87	-21.30
20	NZ_RQSR01000009.1	81706	85618	EHE19_RS12495-EHE19_RS12505	3	-	EHE19_RS12505	STT	38				
21	NZ_RQSR01000010.1	7613	11713	EHE19_RS12740-EHE19_RS12750	3	+	EHE19_RS12740	SNT	256	145-190	0.92		
22	NZ_RQSR01000010.1	76993	79359	EHE19_RS13020-EHE19_RS13030	3	+	EHE19_RS13020	STN	185	99-144	0.96	15-44	-15.9
23	NZ_RQSR01000013.1	8525	11136	EHE19_RS14065-EHE19_RS14075	3	-	EHE19_RS14075	STN	85	17-62	0.95		
24	NZ_RQSR01000013.1	97499	100847	EHE19_RS14405-EHE19_RS14420	4	-	EHE19_RS14420	STTN	67				
25	NZ_RQSR01000022.1	19987	22797	EHE19_RS16685-EHE19_RS16695	3	-	EHE19_RS16695	STN	37				
26	NZ_RQSR01000026.1	11750	17189	EHE19_RS17395-EHE19_RS17410	4	-	EHE19_RS17410	SNTT	418	340-385	0.95	171-204	-22.00
27	NZ_RQSR01000026.1	21486	24014	EHE19_RS17430-EHE19_RS17440	3	-	EHE19_RS17440	STN	248			177-222	-10.30
28	NZ_RQSR01000026.1	28050	30606	EHE19_RS17465-EHE19_RS17475	3	-	EHE19_RS17465	NTS	57	7-52	1		
Ruminiclostridium sufflavum DSM 19573													
1	NZ_QKMR01000001.1	119014	121725	LY28_RS00490-LY28_RS00500	3	-	LY28_RS00500	STN	17				
2	NZ_QKMR01000001.1	166856	171734	LY28_RS00650-LY28_RS00670	5	-	LY28_RS00650	TTNNS	32				
3	NZ_QKMR01000001.1	174777	180408	LY28_RS00690-LY28_RS00710	5	-	LY28_RS00690	TTNNS	51				

4	NZ_QKMR01000001.1	186402	189913	LY28_RS00740-LY28_RS00750	3	-	LY28_RS00750	SNT	92				
5	NZ_QKMR01000001.1	217658	219575	LY28_RS00895-LY28_RS00900	2	-	LY28_RS00900	ST	73			17-56	-17.50
6	NZ_QKMR01000001.1	331191	334981	LY28_RS01325-LY28_RS01335	3	-	LY28_RS01325	NTS	61	3-48	0.98		
7	NZ_QKMR01000001.1	348035	351179	LY28_RS01400-LY28_RS01410	3	-	LY28_RS01410	STT	74			23-75	-18.20
8	NZ_QKMR01000002.1	84802	88220	LY28_RS01715-LY28_RS01725	3	+	LY28_RS01725	TTS	57	10-55	0.9		
9	NZ_QKMR01000002.1	213110	216371	LY28_RS02350-LY28_RS02365	3	+	LY28_RS02350	STT	200	118-163	0.95		
10	NZ_QKMR01000002.1	282261	285807	LY28_RS02625-LY28_RS02635	3	+	LY28_RS02635	NTS	60	3-48	0.98		
11	NZ_QKMR01000003.1	122872	125575	LY28_RS03295-LY28_RS03305	3	-	LY28_RS03305	SNT	201			26-80	-29.70
12	NZ_QKMR01000003.1	185346	189707	LY28_RS03545-LY28_RS03560	4	-	LY28_RS03560	STTN	123			32-99	-28.20
13	NZ_QKMR01000004.1	99262	102105	LY28_RS04400-LY28_RS04410	3	+	LY28_RS04400	STN	7				
14	NZ_QKMR01000004.1	103530	108611	LY28_RS04420-LY28_RS04435	4	+	LY28_RS04420	SNTT	311	215-260	0.94	16-55	-24.50
15	NZ_QKMR01000004.1	233222	236986	LY28_RS04920-LY28_RS04930	3	+	LY28_RS04920	STT	162			88-115	-19.60
16	NZ_QKMR01000005.1	31792	35052	LY28_RS05275-LY28_RS05285	3	+	LY28_RS05275	STT	142	83-128	0.93	17-51	-17.20
17	NZ_QKMR01000005.1	49537	53590	LY28_RS05360-LY28_RS05375	4	+	LY28_RS05365	NSTT	161				
18	NZ_QKMR01000006.1	154583	157560	LY28_RS06885-LY28_RS06895	3	+	LY28_RS06885	STN	27				
19	NZ_QKMR01000006.1	157795	160221	LY28_RS06900-LY28_RS06910	3	+	LY28_RS06910	NTS	34				
20	NZ_QKMR01000007.1	50691	54310	LY28_RS07425-LY28_RS07435	3	-	LY28_RS07425	NTS	102			42-67	-10.50
21	NZ_QKMR01000007.1	145574	148882	LY28_RS07855-LY28_RS07865	3	-	LY28_RS07865	STT	250			195-235	-23.20
22	NZ_QKMR01000008.1	31298	34846	LY28_RS08165-LY28_RS08175	3	-	LY28_RS08165	NTS	59			3-32	-11.70
23	NZ_QKMR01000008.1	54690	57873	LY28_RS08275-LY28_RS08285	3	-	LY28_RS08275	TTS	104				
24	NZ_QKMR01000008.1	109837	113680	LY28_RS08495-LY28_RS08505	3	-	LY28_RS08505	SNT	188	77-122	0.9		
25	NZ_QKMR01000009.1	24165	27623	LY28_RS08815-LY28_RS08825	3	+	LY28_RS08815	STT	386	284-329	0.98	98-146	-27.10
26	NZ_QKMR01000009.1	143981	146604	LY28_RS09220-LY28_RS09230	3	-	LY28_RS09220	NTS	91				
27	NZ_QKMR01000009.1	149510	151915	LY28_RS09250-LY28_RS09260	3	+	LY28_RS09250	STN	144			47-87	-15.00
28	NZ_QKMR01000011.1	104020	108140	LY28_RS10460-LY28_RS10470	3	+	LY28_RS10460	SNT	302			203-241	-25.00
29	NZ_QKMR01000012.1	23052	25443	LY28_RS10760-LY28_RS10770	3	+	LY28_RS10760	STN	210			19-42	-15.40
30	NZ_QKMR01000013.1	1661	4896	LY28_RS11225-LY28_RS11235	3	+	LY28_RS11225	STT	103			12-45	-23.70
31	NZ_QKMR01000014.1	122521	126116	LY28_RS12365-LY28_RS12375	3	+	LY28_RS12375	NTS	73				
32	NZ_QKMR01000022.1	11266	14428	LY28_RS15370-LY28_RS15380	3	-	LY28_RS15370	TTS	29				
33	NZ_QKMR01000022.1	59596	63109	LY28_RS15575-LY28_RS15585	3	-	LY28_RS15575	TTS	70	12-57	0.93		
34	NZ_QKMR01000023.1	35409	38589	LY28_RS15800-LY28_RS15810	3	-	LY28_RS15810	STT	83			41-82	-19.70
35	NZ_QKMR01000029.1	3204	5857	LY28_RS17305-LY28_RS17315	3	-	LY28_RS17315	STN	-12				
36	NZ_QKMR01000030.1	12890	15487	LY28_RS17610-LY28_RS17620	3	+	LY28_RS17610	STN	31				
37	NZ_QKMR01000033.1	23924	26927	LY28_RS18195-LY28_RS18205	3	+	LY28_RS18195	STN	2				
Clostridium cellulovorans 743B													
1	NC_014393.1	72635	76390	CLOCEL_RS00235-CLOCEL_RS00245	3	+	CLOCEL_RS00235	STT	219			43-70	-18.20
2	NC_014393.1	464904	467532	CLOCEL_RS01930-CLOCEL_RS01940	3	+	CLOCEL_RS01930	STN	1				
3	NC_014393.1	546998	549277	CLOCEL_RS02290-glnQ	3	+	CLOCEL_RS02290	STN	67			7-33	-13.40
4	NC_014393.1	634039	637177	CLOCEL_RS02680-CLOCEL_RS02690	3	+	CLOCEL_RS02680	STT	116			56-116	-18.10
5	NC_014393.1	645487	649278	CLOCEL_RS02720-CLOCEL_RS02735	4	+	CLOCEL_RS02720	STTN	28				
6	NC_014393.1	885042	889773	CLOCEL_RS03620-CLOCEL_RS03635	4	+	CLOCEL_RS03620	SNTT	95			44-76	-19.70
7	NC_014393.1	1061880	1065737	CLOCEL_RS04370-CLOCEL_RS04385	4	+	CLOCEL_RS04370	STTN	-2				

8	NC_014393.1	1453190	1457664	CLOCEL_RS05920-CLOCEL_RS05940	5	-	CLOCEL_RS05940	STTNN	21				
10	NC_014393.1	1520919	1524228	CLOCEL_RS06200-CLOCEL_RS06210	3	+	CLOCEL_RS06200	STT	98			27-58	-20.00
11	NC_014393.1	1662149	1668469	CLOCEL_RS06750-CLOCEL_RS06770	5	+	CLOCEL_RS06770	TTNNS	767	625-670	1	245-279	-19.10
12	NC_014393.1	1704650	1709528	CLOCEL_RS07020-CLOCEL_RS07035	4	+	CLOCEL_RS07020	SNTT	241			143-206	-20.60
13	NC_014393.1	1884155	1887051	modA-CLOCEL_RS07710	3	+	modA	STN	125				
14	NC_014393.1	1954037	1956392	CLOCEL_RS07985-CLOCEL_RS07995	3	+	CLOCEL_RS07985	STN	98			3-34	-18.30
15	NC_014393.1	2203963	2207086	CLOCEL_RS09135-CLOCEL_RS09145	3	+	CLOCEL_RS09135	STT	83			30-65	-16.50
16	NC_014393.1	2236843	2240388	CLOCEL_RS09295-CLOCEL_RS09310	4	+	CLOCEL_RS09295	STTN	67			8-35	-17.60
17	NC_014393.1	3147216	3150824	CLOCEL_RS12945-CLOCEL_RS12955	3	-	CLOCEL_RS12945	NTS	55				
18	NC_014393.1	3259237	3261697	CLOCEL_RS13360-CLOCEL_RS13370	3	-	CLOCEL_RS13360	NTS	26				
19	NC_014393.1	3326242	3329500	CLOCEL_RS13590-CLOCEL_RS13600	3	-	CLOCEL_RS13600	STT	68			11-44	-18.60
20	NC_014393.1	3533071	3536873	CLOCEL_RS14375-CLOCEL_RS14385	3	-	CLOCEL_RS14375	NTS	3				
21	NC_014393.1	3931516	3934679	CLOCEL_RS16020-CLOCEL_RS16030	3	-	CLOCEL_RS16030	STT	89			37-62	-19.20
22	NC_014393.1	4188180	4191429	CLOCEL_RS17090-CLOCEL_RS17100	3	+	CLOCEL_RS17100	TTS	465	388-433	1		
23	NC_014393.1	4243995	4247059	CLOCEL_RS17310-CLOCEL_RS17325	4	-	CLOCEL_RS17310	TTNS	-2				
24	NC_014393.1	4620406	4625360	CLOCEL_RS19210-CLOCEL_RS19225	4	-	CLOCEL_RS19220	SSNT	105			23-66	-20.30
25	NC_014393.1	4708327	4711269	CLOCEL_RS19570-CLOCEL_RS19580	3	-	CLOCEL_RS19580	STN	145			49-105	-20.60
26	NC_014393.1	4859322	4862099	CLOCEL_RS20190-CLOCEL_RS20200	3	-	CLOCEL_RS20200	STN	110			15-46	-20.60
27	NC_014393.1	4903628	4907003	CLOCEL_RS20340-CLOCEL_RS20350	3	+	CLOCEL_RS20340	STT	320			198-235	-17.20
28	NC_014393.1	4920044	4923164	CLOCEL_RS20395-CLOCEL_RS20405	3	+	CLOCEL_RS20395	STT	87			25-70	-18.90
29	NC_014393.1	4946707	4951385	CLOCEL_RS20495-CLOCEL_RS20515	5	-	CLOCEL_RS20515	STTNN	60			14-39	-17.20
30	NC_014393.1	5006760	5009161	CLOCEL_RS20725-CLOCEL_RS20735	3	-	CLOCEL_RS20735	STN	-21				

*The gene arrangements were represented by linear order of genes encoding SBP (S), TMD (T), and NBD (N) from 5' to 3'.

**The putative promoters in the intergenic regions (IRs, more than 50 bp) between SBP genes and their cognate translocator genes were predicted by using BDGP prokaryotic promoter prediction program (1). The probability of occurrence of promoter sequences was determined by the use of Promoter prediction (P) score, which can have values from 0 to 1 (a value close to 1 means very high probability) (2). Here, the cutoff value of promoter prediction score was set to more than 0.9.

***The RNA secondary structures (ΔG<-10 kcal mol-1) of the intergenic regions between SBPs and their downstream genes by using On-line Mfold program (3).

Table S2 The RNA secondary structure predicted in intergenic regions between SBPs and their downstream cognate translocator genes

5’SBP gene Cluster	SBP gene	Sequence of stem-loops	Length	Structure	ΔG	Type	3' U-rich tract	poly(U)	Total U
Ruminiclostridium cellulolyticum H10									
CceL_0150-Ccel_0152	CceL_0150	GTCCATTGGAGCATAAGCATATGCTCCAA TGGAT	34	((((((((((((((((((****)))))))))))))*))))))	-25.0	III	TATTTAAAAA	3	4
CceL_0200-Ccel_0202	CceL_0200	GGCGTCCATTTGTTTGCTTTAAGCAAATA ATATTTGGCAAAGAAATGGACGCC	53	((((((((((*((((((****************))))))*)))))))))))	-27.4	IV	AAACCATATG	1	2
CceL_0885-Ccel_0887	CceL_0885	GGGCTGATATCTTATGTATCGGCCC	25	((((((((((*****))))))))))	-16	I	ATTTTTTTGG	7	7
CceL_0998-Ccel_1000	CceL_0998	AACCATCCCTGTATGGGTTGATCTCATACA GGGATTGGTT	40	(((((((((((((((((*****))))))))))))*))))	-25.7	III	TTGGTTAACA	2	4
CceL_1025-Ccel_1027	CceL_1025	TGATAATACAGGTGTTCGGAAGGACATCT GTATTATC	37	(((((((((((((((((****)))))))))))))))))	-26.8	II	AAAATATTTT	4	5
CceL_1133-Ccel_1135	CceL_1133	GTATAATATGATTGGGTACTTATCTTTACA GGTACCCAATCAATTTATAC	50	((((((**(((((((((((((********))))))))))))**))))))	-24.3	III	TTTATACGGT	3	5
CceL_1223-Ccel_1226	CceL_1223	AAAAAACAGGTGTGAAACAGCATAGCCA TTCACACCTGTTTTTT	44	((((((((((((((((***********)))))))))))))))	-20.6	I	TGTTTTTTTA	7	8
CceL_1252-Ccel_1254	CceL_1252	AGAAGGAAAGGATACAGCCGTTTCATATC CTTTCCTTCT	39	((((((((((((((**********)))))))))))))	-18.5	II	TTCTCGTTTA	3	6
CceL_1406-Ccel_1409	CceL_1406	TTTAGCAGTAGTTGATAGAGCCGATTTAG TCGGTAATCGACTACTGCTAAA	51	((((((((((((((((***((((((***))))))*))))))))))))))))	-29.1	IV	TAAAAATATT	2	4
CceL_1764-Ccel_1768	CceL_1768	GAGAATGTCCGTCATTAAAATGAACGGGC ATTCTC	35	(((((((((((((((((****))))*)))))))))))	-20.6	II	TTCTCTTTAA	3	6
CceL_1985-Ccel_1987	CceL_1987	GTGTTCGGTTTGCATATGTCAAGGCTTGAT AAGCTTTCATATATGCAAGCCGAATAC	57	((((((((((((((((((*(((((((***)))))))*))))))))))))))))))))	-34.9	IV	TACAAAGTA A	1	2
CceL_2110-Ccel_2112	CceL_2112	ATGATTTAGGAGAGGGTGAAATTCCCTCT CCTTGATTA	38	((((((*(((((((((*******)))))))))*))))))	-20.5	III	TTGATTATTT	3	7
CceL_2587-Ccel_2589	CceL_2589	CAAAGCAAAGGGGATGTCCAAGGGCACC CCTGTGTTTTG	39	(((((((*(((((*((((****)))))))))*)))))))	-22.1	III	TTTTGTTTCT	4	8
CceL_2686-Ccel_2687	CceL_2687	TATATGAAGTACGCCATGCAATAGCTTGG CATACTTCATATA	42	(((((((((((*((((*((****))*))))*)))))))))))	-22.3	IV	TTCATATAGC	2	4
CceL_2995-Ccel_2997	CceL_2997	TTCAACAAAGGGGAAATATTTTCCCCTTT GTTGAA	35	((((((((((((((((***))))))))))))))))	-22.7	II	TTTGTTGAAT	3	6
CceL_3244-Ccel_3246	CceL_3246	AATACAATTCTCCTCGAATACGAGGAGAA TTGTATT	36	((((((((((((((((****))))))))))))))))	-22.9	II	TTGTATTTTA	4	7
Ruminiclostridium sp. BNL1100									
CLO1100_RS00715-CLO1100_RS00725	CLO1100_RS00715	ATAAGAAGGAGTTGGGATTTGTATTCCAG CTCCTTTTTAT	40	((((((((((((((((((****))))))))))))))))))	-24.1	I	TTTTTATAAG	5	6

CLO1100_RS00750-CLO1100_RS00760	CLO1100_RS00750	TTAAAGTCCATTGGAGCATATTAATTATGCTCCAATGGATATTTAA	46	(((((((((((((((((((*****))))))))))))))))*)))))	-25.8	III	TATTTAAAAA	3	4
CLO1100_RS01030-CLO1100_RS01040	CLO1100_RS01030	GGCGTCCATTTGTTTGCTTAAGTCAACAACACGTGGCAGAAAAATGGACGCC	52	((((((((((*((((((****************))))))*)))))))))))	-26.9	IV	AAACTATATG	1	3
CLO1100_RS01700-CLO1100_RS01710	CLO1100_RS01700	ATATATCCGGGAGATAACACTCCCGGATATAT	32	(((((((((((((******)))))))))))))	-21.3	II	TATATAATAA	1	4
CLO1100_RS02995-CLO1100_RS03005	CLO1100_RS02995	GCAAAGGGGATGTCCACGGGCACCCCTGTGT	31	(((((((*((((((*(((((*((((****))))))))*))))))))))))	-26	III	TTTTGTTTTC	4	8
CLO1100_RS04395-CLO_RS04410	CLO1100_RS04395	TAAAAAAACAGGTGTGAAATAGAATACTGTTTCACACCTGTTTTTTTA	48	((((((((((((((((((((((****))))))))))))))))))))))	-29.8	I	TGTTTTTTTA	7	8
CLO1100_RS04555-CLO1100_RS04565	CLO1100_RS04555	GAGAAGGAAAGGATACAGCCGTTTCATATCCTTTCCTTCTC	41	((((*(((*((((((((((((((***********))))))))))))))))))*))))	-27	III	TTCTCGTTTA	3	6
CLO1100_RS05425-CLO1100_RS05440	CLO1100_RS05425	TTTAGCAGTAGTTGATAGAACCGATTCAGTCGGTAATCGGCTACTGCTAAA	51	(((((((((((((((***((((((***))))))*)))))))))))))))	-28.3	II	TAAAATTTTA	4	5
CLO1100_RS10060-CLO1100_RS10080	CLO1100_RS10080	GAGAATGTCCGTCATTAAAATGAACGGACATTCTC	35	(((((((((((((((****))))*)))))))))))	-21.2	II	TTCTCTTTAA	3	6
CLO1100_RS11300-CLO1100_RS11310	CLO1100_RS11310	TGTGTTCGGTTTGCATATGTCAAGGCTTGATAAGCTTTCATATATGCAAGCCGAATACA	59	(((((((((((((((((((*(((((((***)))))))*))))))))))))))))))))	-35.9	IV	TACAAAGTAA	1	2
CLO1100_RS12515-CLO1100_RS12525	CLO1100_RS12525	ATATAACAAGCTGTAAAAACAGCCTGCTTATAT	33	((((((((*(((((****)))))*))*))))))	-10.3	III	TTATATAGAG	2	4
CLO1100_RS13605-CLO1100_RS13615	CLO1100_RS13615	TACTTTGGCAGGCTGTATAGCCTGCCAAAGTA	32	((((((((((((((****))))))))))))))	-24.4	II	TAAAAATAAC	1	2
CLO1100_RS12140-CLO1100_RS14150	CLO1100_RS14150	GATGATTTAGGAGAGGGTGAAATTCCCTCTCCTTGATTATT	41	(((((((*(((((((((*******)))))))))*)))))))	-21.6	III	TTGATTATTT	3	7
CLO1100_RS14205-CLO1100_RS14215	CLO1100_RS14215	GTATAAGATGATTGGGTACTTTATATTACAGGTACCCAATCAATTTATAC	50	(((((((*((((((((((((********))))))))))))*)))))))	-25.7	III	TCAATTTATA	3	5
CLO1100_RS14590-CLO1100_RS14605	CLO1100_RS14605	GGGGGGATGCACTTCCCCC	19	((((((*******))))))	-13.2	II	AACCTGTTGC	2	3
CLO1100_RS14830-CLO1100_RS14840	CLO1100_RS14840	ATCCATATGGAGCATTTATCTGCTCCATATGGAT	34	((((((((((((((******))))))))))))))	-23.2	III	TAAAATAAAA	1	2
CLO1100_RS14890-CLO1100_RS14900	CLO1100_RS14900	ATATGAGGACGCTTTAAGCGTCCTCATAT	29	(((((*(((((((((((*****)))))))))))))))))	-26.7	III	TATACTCTAA	1	4
CLO1100_RS15125-CLO1100_RS15140	CLO1100_RS15140	TGACCGGTGCAGGCTAGAAACTGTATCGGTCG	32	(((((**(((((((((((********)))))))))))*)))))	-25.5	III	ACTTCACACT	2	3
CLO1100_RS15430-CLO1100_RS15440	CLO1100_RS15440	AGGGCTGATATAATATATATCGGCCCT	27	((((((((((((***))))))))))))	-21.4	I	TGTTTTTAGG	5	6
CLO1100_RS18125-CLO1100_RS18135	CLO1100_RS18135	TTTAAACAATAAGGGGGAAAATATTTTCCCCTTATTGTTTGGA	43	((((((((((((((((((((***))))))))))))))))))))	-24	II	TTATTGTTTG	3	7
Clostridium cellulovorans 743B									
CLOCEL_RS00235-CLOCEL_RS00245	CLOCEL_RS00235	AAGGCATAGAGTTACTACTCTATGCCTT	28	(((((((((((((****)))))))))))))	-20	I	TTTTTTAAAC	6	6

CLOCEL_RS02290-glnQ	CLOCEL_RS02290	AAAAAAGGCAGATCACCTGCCTTTTTT	27	(((((((((((*****)))))))))))	-15.1	I	TTTTTTAGGT	6	7
CLOCEL_RS02680-CLOCEL_RS02690	CLOCEL_RS02680	AATGGTAGGTTAGAAAACAATTCTAGCTTACCATT	35	(((((((((((((((*****)))))))))))))))	-17.9	II	TTTAAAAATT	3	5
CLOCEL_RS03620-CLOCEL_RS03635	CLOCEL_RS03620	GGGGAGCTAGATGTGAAGTGCATCTAGCTCTTT	33	((((((((((((((*****))))))))))))))	-19.5	II	TCTTTTAGAG	4	5
CLOCEL_RS06200-CLOCEL_RS06210	CLOCEL_RS06200	GAGGATGTTCTCGTTACCTAGAGAGCATCCTC	32	((((((((((((********))))))))))))	-21.8	II	TCTAGCTTAA	2	4
CLOCEL_RS07020-CLOCEL_RS07035	CLOCEL_RS07020	GGGCATCTTTAAATGAATAAATGGTCAGTATTAAATGTGACTTCTTATTCAATTTTAGG	64	((((((((**((*(((((((**(((((**********)))))**))))))))*))**))))))))	-23	II	CTTTATTGAT	3	6
modA-CLOCEL_RS07710	modA	TGAACTTTATAAAACTAAAGTTTA	24	(((((((((******)))))))))	-19.7	II	TTTATAGATA	3	5
CLOCEL_RS07985-CLOCEL_RS07995	CLOCEL_RS07985	TAAATAGAATTAGGGGCAACCTTGATTCTATTT	33	((((((((((((((****))))))))))))))	-18.1	I	TATTTTTTTG	7	8
CLOCEL_RS09135-CLOCEL_RS09145	CLOCEL_RS09135	ATAAATAAGGTTTCTCTTGGAAGAAACCTTATTTAT	36	((((((((((((((((******))))))))))))))))	-17.1	II	TTATTTATCA	3	6
CLOCEL_RS09295-CLOCEL_RS09310	CLOCEL_RS09295	TATGGTTGGTTTGAAAAAACCAACCATA	28	((((((((((((****))))))))))))	-18.1	II	TATTTTACAA	4	5
CLOCEL_RS13590-CLOCEL_RS13600	CLOCEL_RS13600	AAAAATTATACAGGGTCAACCCTGTATAATTTTT	34	(((((((((((((((****)))))))))))))))	-19.2	I	TTTTTATAGA	5	6
CLOCEL_RS16020-CLOCEL_RS16030	CLOCEL_RS16030	GGAGCTTGGATGAAAATCCAAGCTCT	26	(((((((((((****)))))))))))	-19.7	II	TCTATAAAAA	1	3
CLOCEL_RS19210-CLOCEL_RS19225	CLOCEL_RS19220	GATTCAATCAAGGATAACAAGAGAAGTTAGTCCTTGATTGAATT	44	(((*(((*((((((((((((((((((*******)))*))))))))))))))))))))))	-21.7	III	TTAAATCAAG	2	3
CLOCEL_RS19570-CLOCEL_RS19580	CLOCEL_RS19580	GTGGTTGAGTATATCACAGCTATTTTATATAAGTAAAGCTGTGGTATATTTATTTAT	57	(((((((((((((((((((((*****))))**)))))))))))))))))	-22.1	III	TATATTTATT	3	7
CLOCEL_RS20190-CLOCEL_RS20200	CLOCEL_RS20200	TCAGGCTAATAATGAATTCATTATTAGCCTGA	32	((((((((((((((****))))))))))))))	-22.7	II	TGATTCTGCG	2	4
CLOCEL_RS20340-CLOCEL_RS20350	CLOCEL_RS20340	CAATAAAAATGTCTTAGTGAGACTAAGACATTTTTATT	38	((((((((((((((((*****))))))))))))))))	-17.3	I	TTTTTATTTA	5	8
CLOCEL_RS20395-CLOCEL_RS20405	CLOCEL_RS20395	AAAACCATTTGCTGAATTACTACATAGATATTTGGCAAATGGTTTT	46	((((((((((((**(((**((****))**)))**))))))))))))	-19.4	II	TTTTATAAGG	4	5
CLOCEL_RS20495-CLOCEL_RS20515	CLOCEL_RS20515	TGGGGAGAACTAGAAAGTTCTCCCTA	26	(((((**(((((((((((****)))))))))))*)))))	-17.4	III	TAAATTTTAA	4	5
Ruminiclostridium papyrosolvens DSM2782									
CPAP_RS01065-CPAP_RS01075	CPAP_RS01065	GTATAAGACGATTGGGTACTTTCTCTTACAGGTACCCAATCAACTTATAC	50	(((((((**((((((((((((********))))))))))))**)))))))	-24.3	III	TCAACTTATA	2	4
CPAP_RS01905-CPAP_RS01915	CPAP_RS01905	TGCTTTGGCAGGCTTTACAGCCTGCCAAAGTA	32	((((((((((((((****))))))))))))))	-24.2	II	TAAAAATAAC	1	2
CPAP_RS02915-CPAP_RS02930	CPAP_RS02915	AAAAAACAGGTGTGAATGTAATACTGTTTCACACCTGTTTTTT	43	((((((((((((((((***********))))))))))))))))	-20.4	I	TGTTTTTTTA	7	8

CPAP_RS03065-CPAP_RS03075	CPAP_RS03065	AACAAAGAAGGAAAGGATACAGCCGTTTCATATCCTTTCCTTCTTGTT	48	(((((*(((((((((((((***********)))))))))))))))))))	-20.8	III	TTCTTGTTTC	3	7
CPAP_RS04210-CPAP_RS04220	CPAP_RS04210	AATACATATCCCTATATGAAATTCAACTTCATATAGGGATATTATT	46	((((*(((((((((((((((*******)))))))))))))))))))	-20.3	III	TATTATTAAA	2	5
CPAP_RS04410-CPAP_RS04425	CPAP_RS04410	TTTAGCAGTAGTTGATAGAACCGATTCATTCGGTAATCGGCTACTGCTAAA	51	(((((((((((((((***(((((*****)))))*)))))))))))))))	-28.6	IV	TAAAAAATTT	3	4
CPAP_RS04990-CPAP_RS05000	CPAP_RS04990	TGTGTTCGGTTTGCATATGTCAAGGCTTGATAAGCTTTCATATATGCAAGCCGAATACA	59	(((((((((((((((((((*(((((((***)))))))*)))))))))))))))))))	-35.9	IV	AAGTAAGGGA	1	1
CPAP_RS06205-CPAP_RS06225	CPAP_RS06205	GAGAATGTCCGTCATTAAAATGCACGGGCATTCTC	35	((((((((((((((****)))*)))))))))))	-20.6	II	TTCTCTTTAA	3	6
CPAP_RS08885-CPAP_RS08895	CPAP_RS08895	AGGGGATGTCCATGGGCACCCCT	53	(((((*((((****)))))))))	-13.8	II	TGTGTTTTG	4	6
CPAP_RS10395-CPAP_RS10405	CPAP_RS10395	GTCCATTGGAGCATATTTTATATGCTCCAATGGAT	35	(((((((((((((((***)))))))))))))))	-24.4	II	TAATTAAAAA	2	3
CPAP_RS10725-CPAP_RS10735	CPAP_RS10725	GGCGTCCATTTGTTTGCTTGAGTCAATAACACATGGCAGGGAAATGGACGCC	52	((((((((((*((((((((*((*****))*))*))))))*))))))))))	-28.3	IV	AAATTATATG	2	4
CPAP_RS11190-pstA	CPAP_RS11190	AAAGGAATCTGGTTGAGTTTACTCTCCAGCTTCCTTT	37	(((((((*((((**(((****)))*))))*)))))))	-16.3	III	TTCCTTTTAC	4	6
CPAP_RS14495-CPAP_RS14505	CPAP_RS14495	CTTTGAGACAAATACTGCAATTTTAATGGCGGTATTTGTCTCAGGG	46	((((((((((((((((((**********))))))))))))))))))	-26.1	II	ATATATAACA	1	3
CPAP_RS16530-CPAP_RS21395	CPAP_RS16530	GACAGGGTTTATCTTTTTTTGGGTATACCCTGTT	34	((((((((*((((((******)))))*))))))))	-16.5	II	TGTTTTGTCA	4	6
CTER_RS18030-CTER_RS18040	CPAP_RS18030	ATATAACAGGCTGTAAAATCAGCCTGCTTATAT	33	(((((((((((((******)))))))*))))))	-15.1	III	TTATATTAAA	2	5
CPAP_RS18615-CPAP_RS18625	CPAP_RS18615	AAAGATTCAACTGGTGCTCTAGGTTGAATCTTT	33	((((((((((((((***))))*))))))))))	-13.2	IV	CTTTTCTTCC	4	7
CPAP_RS19285-CPAP_RS19295	CPAP_RS19295	GATGATTTAGGAGAGGGTGAAATTCCCTCTCCTTGATTATT	41	(((((((*(((((((((*******)))))))))*)))))))	-21.6	III	TTATTTTACT	4	7
CPAP_RS19385-CPAP_RS19395	CPAP_RS19385	ACTTCAAAAAAGGGCTGATATAATATATATCGGCCCTGTTTTTAGGT	47	((((*((((*(((((((((((((***)))))))))))))*))))*))))	-21.4	I	TGTTTTTAGG	5	6
CPAP_RS19580-CPAP_RS19595	CPAP_RS19580	GCGTCCGGTATGTAGTGCTTAAATTTATAGCTTAAATTCCTTCTCCATACCGGAAGT	57	((((**((*((((((((((*((******((((((*****))))))****))*))))))))))*))**))))	-26.8	IV	TTGGTTGCAT	2	5
Ruminiclostridium cellobioparum subsp. termitidis CT1112									
CTER_RS00125-CTER_RS00135	CTER_RS00135	TGGGGTAGTAACTGCAAGAATATTATCAGCTACTACCCCA	40	((((((((((*((((************)))*))))))))))	-18.6	II	AAATTTCAAT	3	4
CTER_RS00215-CTER_RS00225	CTER_RS00225	TGATGGGCTGTTTTATAAAGTCGTTATTCTTCTGACTTTAAGACAGCCCTCA	52	(((((((((((**((((((*********))))))))))))))))))	-22.7	I	TTTTTGTTTG	5	8
CTER_RS00840-CTER_RS00850	CTER_RS00850	GGAATCGAAAAACAGGTTTAAACTGTTTTTCGATTCC	37	(((((((((((((((*******)))))))))))))))	-22.2	I	TTTTTCGATT	5	7
CTER_RS01275-CTER_RS01290	CTER_RS01290	TATTAGCAGTAGTTGAAGCCCCTTCGCCAGCATTGGACTCGACTACTGCTAATA	54	((((((((((((((((**((**************))**))))))))))))))))))	-24.4	II	TAAGGTTTTA	4	5

CTER_RS01405-CTER_RS01415	CTER_RS01415	AAAAGGCAGGTGTTAAACAAACAGCTTACCGCCTGCCTTTT	41	(((((((((((*(((*********)))*)))))))))))	-17.9	I	TTTTTCACAC	5	5
CTER_RS02285-CTER_RS02295	CTER_RS02285	GAATATAAATAAATGATTTCTCCTGGTATTTGAGGGTCATTTATTTATATTC	52	(((((((((((((((((*(((*********))))))))))))))))))))	-20.1	II	TTTATTTATA	3	7
CTER_RS02395-CTER_RS02405	CTER_RS02405	TCCTTTATCCGCTTAGCGGTAGAGGA	26	((((((((*((((***))))))))))))	-16	III	GAGACCAAAT	1	1
CTER_RS02730-CTER_RS02740	CTER_RS02740	TCATCAGGGTACAGGCCTGGAATTTCGGGTAAAAACTTATTATGAGTCCTGAAAATTT	76	(((*((((((((*(((((((((((((((*****((((*****)))))))) *)))))))))))*)))))))))))	-33.1	III	TGTACTTTGT	3	6
pstA-CTER_RS03150	CTER_RS03150	AATATAAAAGGAGACTGGTTGCTTTAACCGGCTTCCTTTTATATT	45	(((((((((((**(((((((***)))))))**)))))))))))	-21	III	TTTTATATTA	4	7
CTER_RS03440-CTER-RS03450	CTER_RS03450	AAAGGGGCGGGACAAAAGTCCTGCCCCTTTT	30	((((((((((((****))))))))))))	-22.3	II	TTTTATATAA	4	6
CTER_RS03745-CTER_RS03755	CTER_RS03755	AGGCTGTCTCAAAATAAAATATTGAGATGGCCT	33	((((((((((((*********))))))))))))	-18.8	II	TTCTTTTCAG	4	6
CTER_RS04260-CTER_RS04270	CTER_RS04270	TTTTATGCCGCCCCGGTTCAGTTGGGGCCGGGGCGGTATAAA	42	((((((((((((((((((*****))))))))))))))))))	-33.9	II	TATAAAACAG	1	2
CTER_RS05035-CTER_RS05055	CTER_RS05055	TTAAAAGATGTCCGGATTTTTTGTCCGGGCATTCTTTTGA	40	((((((((((((((((*****)))))))))*)))))))	-21.5	III	TTCTTTTGAA	4	6
CTER_RS05770-CTER_RS05780	CTER_RS05770	TTTTTATGGGGGAATGGCAGCTACGTTATTCCCCCATAAAAA	42	((((((((((((((((((******))))))))))))))))))	-29.1	II	CTTTAAAGTG	3	4
CTER_RS06430-CTER_RS06440	CTER_RS06440	GCAGCCAGGTTGAAAAGCCTGGCTG	25	(((((((((((****)))))))))))	-21.8	II	TGTATTTTCC	4	6
CTER_RS06625-CTER_RS06635	CTER_RS06635	AGGAGTTGGGATAACTTATTCCAACTCCT	29	((((((((((((((***))))))))))))))	-21.5	I	TTTTTCAGAA	5	5
CTER_RS06730-CTER_RS06740	CTER_RS06740	AGGGGCTAGGTCAAATTTGGGCTTGACTTAGCCCCT	36	(((((*((((((((((((((********))))))))))))))**)))))	-29	III	TTATGGTTAC	2	5
CTER_RS06975-CTER_RS06985	CTER_RS06985	TTTCATGCAGGCAGATTTGCTCTGCCTGCATGAAA	35	(((((((((((((((*****)))))))))))))))	-25.7	II	ATTTTAAAAG	4	4
CTER_RS07165-CTER_RS07175	CTER_RS07175	AAAACAATTGCTGCAGCAGCTGATGTCTGCAGCAATTGTTTT	42	((((((((((((((((((*****))*))))))))))))))))	-26.7	II	TTGTTTTGGA	4	6
CTER_RS07200-CTER_RS07210	CTER_RS07210	TATAAAAATTCAGGTGCTGTTAAGCACCTGAATTTTTATA	40	((((((((((((((((((****))))))))))))))))))	-25.1	I	TTTTTATACG	5	6
CTER_RS07480-CTER_RS07490	CTER_RS07490	GCGGCCTTTAGAAGTCCTTTTCTAGAGGCCGT	32	((((((((((((((****))))))))))))))	-22.2	II	TATGAATATC	1	4
CTER_RS07550-CTER_RS07560	CTER_RS07560	TGGGGCTTTTGTTAATTAGTCTTTTTGCAAAAGCCCTA	38	((((((((((((*((********))*))))))))))))	-18.7	II	TAAATATTTC	3	5
CTER_RS07900-CTER_RS07910	CTER_RS07910	AACTGACCTGAGATGGAGGGGAAATTCTGCAATCTCAGGTCAGTT	45	(((((((((((((((*((((****))))*))*)))))))))))))	-28.9	II	TTAAAAATTT	2	5
CTER_RS08390-CTER_RS08400	CTER_RS08400	ATAGGGGCCTATTTTTTCTAGTGCCCCTAT	30	(((((((((((*******)))*))))))))	-16.8	II	TATCATTAAG	2	4
CTER_RS08655-CTER_RS08665	CTER_RS08665	GGAGGGGGCCCTTCACCGGGGCCCTCTT	28	(((((((((((((((*****))))))))))))***))))	-23.4	III	TCTTTTTCAC	5	6

CTER_RS08870-CTER_RS08890	CTER_RS08890	CTGCCAGAAGTGTGACCTTGCACTTCTGGCAG	32	(((((((((((((******)))))))))))))	-24.3	II	ATATTATTTT	4	7
CTER_RS09185-CTER_RS09195	CTER_RS09195	AAGTACAACTTCAATAAATGGAAAGCTCCATTTATTGAAGTTGGTACTT	49	((((((((((((((((((((((****)))))))))))))))))*)))))	-27.9	III	TACTTCCGTC	2	4
CTER_RS09270-CTER_RS09280	CTER_RS09280	GTAAATGGGAAACGGTTATTGAGCCGTTTCCCATTTAT	38	(((((((((((((((((****)))))))))))))))))	-29.6	II	TTTATGGAGG	3	4
CTER_RS09365-CTER_RS09375	CTER_RS09375	CCCGGTATGTTTTAACGGACATACCGGG	28	(((((((((((******)))))))))))	-18.6	II	CACTTATGGA	2	3
CTER_RS09475-CTER_RS09485	CTER_RS09485	GGTTATACAGGCATATCCGGTGGGACGGATATGCCGTAAAACC	43	(((((*(((*(((((((((******))))))))))))*)))))	-26.1	III	GGAGAATCAA	1	1
CTER_RS09535-CTER_RS09555	CTER_RS09555	AGCTTGCTGTGGCAGGTGAAAAATCCTGCCGCAGCGGCT	39	(((((*((((((((((********)))))))))))))))	-29.1	III	TTTTCTTATG	4	7
CTER_RS09610-CTER_RS09620	CTER_RS09610	CTTACGGTGCCATGCTTTCAGCGGGTACCGTGG	33	((((((((((*(((*****)))))))))))))	-20.4	II	TGGTCTGGAG	1	3
CTER_RS10035-CTER_RS10045	CTER_RS10045	GGAAGGAAAGGACTCGTGCAGGTATTGACAGTATCCTTTCCTTCT	45	((((((((((((****(((**((****))*)))))))))))))))	-23.5	II	TTCTTGTCTA	3	6
CTER_RS10660-CTER_RS10670	CTER_RS10670	GGGTGTCCGCTCGGAAACTACATGCGAGTCAGCATTTTCTCAGCGGACACCT	52	(((((((((((*(((((****((((******)))))))))*)))))))))))	-29.4	II	TTTGCCGTTA	4	5
CTER_RS10895-CTER_RS10905	CTER_RS10905	TGCAATGGAATAGGAGAATTTAATTCTCTTATTCCATTGCA	41	(((((((((((((((((((***)))))))))))))))))))	-27.2	II	TTGCACAGTA	2	3
CTER_RS11240-CTER_RS11250	CTER_RS11250	GCCGGAGGATAAAACCTCCGGC	22	((((((((******))))))))	-17	II	CATAATTTAT	3	5
CTER_RS11275-CTER_RS11285	CTER_RS11285	TGGCGTCCATTTGATACTTATTTGGTAATTCACCGGTAGCAAATGGACGCCA	52	(((((((((((((*((((*****(((*****)))))))*)))))))))))))	-26.8	II	AATACTTATG	2	4
CTER_RS12425-CTER_RS12435	CTER_RS12435	GCAGGGTGAATGTGTATTCATCCTGC	26	(((((((((((****)))))))))))	-18.4	II	CTGCCTGCGC	2	2
CTER_RS12610-CTER_RS12620	CTER_RS12620	CTGTATCAAAAAATAAGACAATTGCAATACAAGATTTTCACATGTATTTCAATTGTCTT	71	((((*(((((((*(((((((((((*((((((***********))))))*))))))))))))))))))))))	-27.3	I	AATCATTTTT	5	6
CTER_RS13035-CTER_RS13045	CTER_RS13045	ATCTGGAAAAAGGGGATGCAAGTCCCCTTTTTTTAGAT	38	(((((((((((((((((****)))))))))))))))))	-23.5	I	TTTTTTTAGA	7	7
CTER_RS13300-CTER_RS13310	CTER_RS13310	ACCCGCTAATTCGATATGGCACAGTGCCATATCGAATTAGCAGGT	45	(((***(((*((((((((((((*****))))))))))))*))))))	-31	III	TTACTCTGGA	2	4
CTER_RS13665-CTER_RS13675	CTER_RS13675	TGAGTCAGGTCGGAAGGATATTTTCAAACAACTTCGGATCTGACTCA	47	(((((((((((*((((***************))))*)))))))))))	-25.1	II	AACTTAAAAT	2	3
CTER_RS14530-CTER_RS14540	CTER_RS14540	GAATTTTATGGGCGAAGAAACTGTTTGTTTCAACCGCCCATAAAATTT	48	((((((((((((((**(((((*****)))))***))))))))))))))	-30.9	II	TTTAAAGGTT	3	5
CTER_RS14610-CTER_RS14620	CTER_RS14620	TATGAAACCATGAAAATTTAATTTTCATGGTTTCATA	37	(((((((((((((((((***)))))))))))))))))	-20.1	II	TTTCATAAAA	3	4
yjfF-CTER_RS14950	CTER_RS14950	TAATTATTTCGGGGATACAGTCATGTATCCCCGAAAAATTA	41	(((((*(((((((((((((****))))))))))))))))))	-22.9	III	TTAATCAAAA	2	3
CTER_RS15260-CTER_RS15280	CTER_RS15280	GCTGAACGCTCCCCACACGCCGAGGTGAGCGTTCAGC	37	(((((((((((*((*********))*)))))))))))	-21.3	III	GTTCAGCGCG	2	3

CTER_RS15370-CTER_RS15380	CTER_RS15380	TTTGTTGTTTATTTGTACATGGGAGGGCCAATCCGGCCGCCCATGTACAAATAAGACA	63	((((((((((((((((((((**((((*****))))*)))))))))))))*))))))))	-38.3	IV	AGACGGAGCG	1	0
CTER_RS15620-CTER_RS15630	CTER_RS15630	TAAACCGGGAGGCTGTATCGGTAAGCCGGTACAGTTCCCAGGTTTA	46	((((((((((*((((((((****))))))))))))*))))))	-29	III	TTTAAATGAT	3	5
CTER_RS15670-CTER_RS15680	CTER_RS15680	ACAATATAAGAAGGAGGTTTTCCTTCTTATATTGT	35	((((((((((((((((***))))))))))))))))	-22.8	II	TTATATTGTC	2	6
CTER_RS15835-CTER_RS15845	CTER_RS15845	TATATAAATTACGGACGGGCAGACAATGTACCGTCCGTAATTTATATA	48	((((((((((((((((((((*****)))*))))))))))))))))))	-30.6	II	TAATTTATAT	3	6
CTER_RS16060-CTER_RS16080	CTER_RS16080	GTAGGGGGGATATAATATTCATATCGCCCTTAC	33	((((((((((((*******))))))))))))	-18.4	II	TTACTTCTAT	2	6
CTER_RS16620-CTER_RS16630	CTER_RS16630	TTTTGCGAAACAGGAGGTTTCATGGCCTTCTGTTTCGCAAAA	42	((((((((((((((((((****))))))))))))))))))	-27.9	II	TATTATCAGG	2	4
CTER_RS16765-CTER_RS16775	CTER_RS16775	AGGGCTGATACCGACAGGTATCAGCCTT	28	(((((**(((((((((((****)))))))))))*)))))	-23.2	III	TTAATTGGCA	2	4
CTER_RS17275-CTER_RS17285	CTER_RS17285	CGGGAGATAACCTGTATCAGGTTACTCTCCCG	32	((((((((((((((***))))))))*)))))))	-21.2	II	GTATTCTATC	2	5
ehuA-CTER_RS17320	CTER_RS17320	AACAAACTATAGGGCTTTCCAGCCCTATAGATTTGTT	37	(((((((((((((((****)))))))))))*))))))	-20.4	III	TTATTTTAAT	4	7
CTER_RS17495-CTER_RS17505	CTER_RS17505	TTATTATACGGAACATGGTGGGAAACCACCATGTTTCCTTATAATGG	47	((((((((*(((((((((((****)))))))))))*))*))))))))	-27.3	III	TGTTTCCTTA	2	6
CTER_RS17775-CTER_RS17785	CTER_RS17785	ATATTTATAGGGTGGTGGCATATGTGTTATCACCCTATAGATGT	44	((((((((((((((((((((****))))))))))))))))))))	-26.7	II	TATAGATGTG	3	4
CTER_RS17825-CTER_RS17835	CTER_RS17835	AGACCAATGCGGCCGGGTACGCTGCTGCATTGGTTT	36	((((**((((((((((((((****))**))))))))))))**))))	-23.3	III	TTGGTTTTAT	4	7
CTER_RS18000-CTER_RS18010	CTER_RS18000	TGAAAATTTTCCGGAAATCGCATTGGTTTCCGGAAAATTTTTA	43	((((((((((((((((((*****))))))))))))))))))	-23.4	I	TTTTTAAGGA	5	5
CTER_RS18030-CTER_RS18040	CTER_RS18030	TATAATTGATTGGGTATCACTCATCTGATACCCATCAATTATA	43	((((((((((*((((((((******))))))))))))))))))	-23.9	III	TTATATTACT	2	6
CTER_RS18135-CTER_RS18145	CTER_RS18135	TAAGCGTGCAAATAAATTAACATAATTACAGTTTAATTCATGCTTA	46	((((((((*((*((((((***********))))))*))))))))))	-11.2	III	TTAAAAGGAG	2	2
CTER_RS18485-CTER_RS18495	CTER_RS18495	ACAGACAGCTGTTCTGCTTGAAGAAGTGCATGAACAGCTGTTTGT	45	((((((((((((((((**********)))*)))))))))))))	-25.5	II	TGTTTGTATA	3	6
CTER_RS19900-CTER_RS19910	CTER_RS19910	AGTTTCTGTCCAGTATATTAGTAAGGTCGGCTTGAAAGACTTCATTAATATGCTGGAT	65	((((((((((((((((((((*(((((*********)))))*))))))))))))))))))))	-32.4	II	TTAAAACCGG	2	2
CTER_RS20240-CTER_RS20250	CTER_RS20250	GGCTAATGCCCTTCGTTTAGGAAATCCGGGGCATTAGCC	39	((((((((((((*******((****))))))))))))))	-31.8	II	TGAATTGGTC	2	4
CTER_RS20280-CTER_RS20290	CTER_RS20290	TTGAGCAAATGGCCGGTTAGCATGTCTATCGGTCATTGCTCAA	43	((((((((*((((((((*((*****))))))))))))))))))	-20.7	III	CTTATTTAGG	3	5
CTER_RS20400-CTER_RS20410	CTER_RS20410	AATGCATTCTCCTCGGAAGGTTCCGGGGAGAATTGCATT	39	(((*((((((((((((((((****)))))))))))*)))))))))	-24.3	III	TTGCATTTAA	3	5
CTER_RS20450-CTER_RS20460	CTER_RS20460	AGAAGTTGCACGGCAAACAAGTCATGCAACTTCT	34	((((((((((*(((******)))*))))))))))	-18.9	II	TTCTTTACCT	3	6

CTER_RS20465-CTER_RS20475	CTER_RS20475	GGCTGCCTTTGCGGCT	16	(((((*((((((****))))))))))	-10.1	III	TCAAAGCCAT	1	2
CTER_RS20610-CTER_RS20620	CTER_RS20620	CTTAAATATGAATCACTATAGCTGTTATAGTGATTTATATTTAAG	45	((((((((((((((((((((***))))))))))))))))))))	-22.1	II	TTTATATTTA	3	7
CTER_RS20640-CTER_RS20650	CTER_RS20650	CATAATCAGTGGGAGATTTATACTCGCACTGATTATG	37	(((((((((((*(((*******)))*)))))))))))	-29.6	II	TTATGTAATT	2	6
CTER_RS20785-CTER_RS20795	CTER_RS20795	TTATGCAGGAAGGATATCTATATGGGTATCCTTTCTGCATAG	42	(((((((((((((((((((((****)))))))))))))))))))))	-29.8	II	TAGTTACTAT	3	5
CTER_RS21035-CTER_RS21045	CTER_RS21045	ATGATCAAGGGGATCTGTAGTAAAATATGGATCCCCTGATTAT	43	(((((((*(((((((((((******))))))))))))))))))	-26.3	III	TGATTATTAT	2	6
CTER_RS21300-CTER_RS21310	CTER_RS21310	GTGCAGGCACCTTTCAGAAGCTGGAAGGCGCCTGTAC	37	((((((((*((((((((***))))))))*))))))))	-26.6	II	TGTACAATAT	1	4
CTER_RS21335-CTER_RS21345	CTER_RS21345	TCTGTTTTGGGCAAATTCATTTTGCACAAAGCAGA	35	(((((((((*(((((*****)))))*)))))))))	-16	IV	GCAGAAATGG	1	1
CTER_RS21790-CTER_RS21800	CTER_RS21800	GCTTGGACCGTTAAACACGGTCCGGC	26	(((((*(((((((*****))))))))))))	-17.1	III	AATCTTCCTT	2	5
CTER_RS23560-CTER_RS23570	CTER_RS23570	GGGGATAAGGTCAATGGATTTTACGGTACCTGTAAAATTGTCGGCTTTATCCCC	54	(((((((((((((((*(**(*((((((((***))))))))**)*)*))))))))))))*)))	-30.3	IV	ATGTTCAATA	3	4
CTER_RS23955-CTER_RS23965	CTER_RS23965	GATGGGACTGTCTCAGAATGTACTTTTGAGGCAATCCCATC	41	(((((((*((((((((((*****))))))))))*)))))))	-24.3	III	TAACTTAATA	2	4
CTER_RS24130-CTER_RS24140	CTER_RS24140	AAAGTATGCGCCTGAAATTGCCGGTGAAATACAAGTATTGAGCTGTACAGATTTCAGG	68	((((((((((((((((((**((((**((((****))))**))))****))))))))))))))))))))	-33.3	II	TGTACTTTAC	3	5
CTER_RS24275-CTER_RS24285	CTER_RS24285	AAATCGAAGGTTCGGGTTTCAGGTTCCTGGGCTTGAAAGGCTTCGGCCTGAAACCTG	61	(((((((*(((((((((((((((**((**((((*****))))**))))))))))))))))*)))))))	-36.1	III	TATTGGTTTT	4	7
CTER_RS24815-CTER_RS24825	CTER_RS24825	CGGACCTGCAAATATATGATTTGCAGGTCTG	31	(((((((((((((*****)))))))))))))	-20.6	II	TCTGACTTTA	3	5
CTER_RS24910-CTER_RS24925	CTER_RS24925	TTAAAGGGGAAGGAAACACCTTCCCCTTTAA	31	(((((((((((((*****)))))))))))))	-20.4	II	TTTAATTGTA	3	6
CTER_RS25455-CTER_RS25465	CTER_RS25465	TCAAGGGGATAATTATTATCCCCTTGA	27	((((((((((((***))))))))))))	-17.3	II	TTGAATATTA	2	5
CTER_RS25585-CTER_RS25600	CTER_RS25600	AATTTTCCGGCAGTAGCTTTGATGCTGCCGGAAAATT	37	(((((((((((((((*******)))))))))))))))	-24.5	II	TTTAAAAGTT	3	5
CTER_RS26320-CTER_RS26330	CTER_RS26330	TTTTGGGAGGATGACCTGATAGACCTCTCGATGGGTCTTACAGGTTATCCTCTTAAAA	58	(((((((((((((((((**((((((******))))))**))))))))))))))))))	-36.7	IV	TTATCCTCTT	2	6
CTER_RS26505-CTER_RS26515	CTER_RS26515	TGCAGAGTCGCCATTTTGCGGTAACTGATCCATAAAGAGCTTCAGTTGGTGAGATGGC	68	(((((((((*((((((((**(((((((*************))))))))**))))))))**)))))))))	-33.3	IV	TCTCTGTAAT	1	5
CTER_RS26295-CTER_RS27340	CTER_RS27340	AAATGCGGAGGTGAAGCGGAGTAAATCCGCTTCACTCTGCCAGCATTT	48	(((((((((((((((((((((*****))))))))))*))*))*))))))	-30.2	III	TTTATGGGAA	3	4
Ruminiclostridium sp.MA18									
EHE19_RS01610-EHE19_RS01620	EHE19_RS01610	TATTGCTCGATGTGTTATCTAAATATAGACACATCGAGCAATA	43	((((((((((((((((***********))))))))))))))))	-24.9	II	TATATTAAAA	2	4

EHE19_RS01740-EHE19_RS01750	EHE19_RS01750	GCAAAAAGCCGCAAGCAGCTTTTTGT	26	(((((((((((********)))))))))))*)))	-10.7	III	TTAGTTTGAT	3	6
EHE19_RS06740-EHE19_RS06755	EHE19_RS06755	GAAATGCGCGGCACCAAGTATTTGGATATTATTTTCAGTGCTTGGGTCGTGCATTTC	57	(((((((((((*((((((((*(((*******))))))))))))))))))))))))))	-32	III	TTTCTCAAAT	3	5
EHE19_RS10895-EHE19_RS10905	EHE19_RS10895	GGCTGTTGCAAAGCAAGAAATAATATAATCAATAGTTGTTTTGCAACAGCT	51	((((((((((((((*****************))))))))))))))	-24.6	II	TACTTGCTTTT	4	6
EHE19_RS13020-EHE19_RS13030	EHE19_RS13020	TTGGGGCTGTTTCGTTTGAGGCAGTCTCAA	30	(((((((((((((****)))))))))))))	-16.7	II	TTTATGTCAA	3	5
EHE19_RS17395-EHE19_RS17410	EHE19_RS17410	ATAGAAGGGAAAGGAAACAGCCTTTCCCTTCTAT	34	((((((((((((((******))))))))))))))	-22.5	II	TTCTATAATA	2	5
Ruminiclostridium josui JCM17888									
K412_RS0100595-K412_RS0100605	K412_RS0100605	TGTGTTCGGTTTGCATATGTCAAGGCTTGATTAGCTTTTATATATGCAAGCCGAATACA	59	((((((((((((((((((*((((((*****))))))*))))))))))))))))))))))	-36.4	III	TACAAGTAAA	1	2
K412_RS0101360-K412_RS0101370	K412_RS0101370	GATGATTTAGGAGAGGGTGAAATTCCCTCTCCTTGATTATT	41	((((((((**(((((((*(((((((((*******)))))))))*)))))))*))))))))	-26.4	III	TTATTTTAAT	4	7
K412_RS0101760-K412_RS0101775	K412_RS0101775	AAAACCTACATCGTGTAGGTTTT	23	(((((((((*****)))))))))	-12.1	II	TTTTCCGGTA	4	5
K412_RS0102900-K412_RS0102910	K412_RS0102910	AGAAGGGAAGGATACAGCCGTTTCATATCCTTTCCTTCT	39	((((**((((((((((((((**********))))))))))))))))))	-19.9	III	TTCTTGTTTC	3	7
K412_RS0103050-K412_RS0103065	K412_RS0103065	AAAAAACAGGTGTGAATAAAATACTGTTTCACACCTGTTTTTT	43	((((((((((((((((**********))))))))))))))))	-22.4	I	TGTTTTTTTA	7	8
K412_RS0104305-K412_RS0104315	K412_RS0104315	AAGCAAAGGGGATGTCTACGGGCACCCCCTGTGTTT	36	(((((*((((((*(((((*((((****))))*)))))*)))))))))))	-24	III	TTTTGTTTTC	4	8
K412_RS0106255-K412_RS0106265	K412_RS0106265	TAATCGAATGGGGAACTATAGTGTTTCCTGTTCGATTA	38	((((((((((((((((******))))))))))))))))	-20.7	II	TTATTGGTTA	2	6
K412_RS0109175-K412_RS0109185	K412_RS0109175	GTCCATTGGAGCATATAAATTATGCTCCAATGGAT	35	((((((((((((((((((((*****))))))))))))))))*)))))	-26.5	III	TATTTAAAAA	3	4
K412_RS0109450-K412_RS0109460	K412_RS0109450	GGCGTCCATTTGTTTGCTTATGTCAACAACTCGGGGCAAAAAATGGACGCT	51	((((((((((*(((((((**((*****))***))))))))))))))))))	-24	II	TATATTATAT	2	6
K412_RS0111250-K412_RS0111260	K412_RS0111250	AAGGGCTGATACCTCATATATCGGCCCTT	29	(((((((((((*******)))))))))))	-16.7	I	TTTTTTCAGG	6	6
K412_RS0111945-K412_RS0111960	K412_RS0111945	AGGGGGATATTTCCCCCT	18	(((((((****)))))))	-13.7	II	TACCTTTGTA	3	5
K412_RS0112290-K412_RS0112300	K412_RS0112290	GTATAAAATGATTGGGTACTTTAACTTACAGGTACCCAATCAATTTATAC	50	(((((((*(((((((((((((********))))))))))))))*)))))))	-26	III	TCAATTTATA	3	5
K412_RS0113650-K412_RS0113665	K412_RS0113650	TTTAGCAGTAGTTGATAGAACCGATTTAGTCGGTAATCGGCTACTGCTAAA	51	((((((((((((((((***((((((***))))))*))))))))))))))))	-28.3	IV	ATTTTACCAG	4	4
K412_RS0117615-K412_RS0117635	K412_RS0117615	GAGAATGTCCGTCATCATTAAAATGCACGGACATTCTC	38	(((((((((((((((*******)))*))))))))))))	-25.5	II	TTCTCTTTAA	3	6
Ruminiclostridium sufflavum DSM 19573									

LY28_RS01400-LY28_RS01410	LY28_RS01410	TATTGTTAGGGGAGCATTCCCCTAACAATG	30	((((((((((((******))))))))))))	-19.2	II	TGGTTCTAAA	2	4
LY28_RS03295-LY28_RS03305	LY28_RS03305	AAGGATACCGTAGCAGGAATGGCAAGGATACATTGCTTCCTGTTAAGGTATCCTT	55	(((((((((*((((((((**(((((*******)))))))))))))*)))))))))	-30	III	TTGAATGTATT	2	6
LY28_RS03545-LY28_RS03560	LY28_RS03560	TCAGCCGGCATCCGATATGCAGCACTTAAGCTTATTGCTCATATTCCGCCATACCGGAA	68	(((((((((*((((*(((((********(((*****)))********))*)))*))))*)))))))))	-32.5	II	TTGAATGTAT	2	5
LY28_RS04420-LY28_RS04435	LY28_RS04420	TGTTTCTAATGTATTCAGCTTGCTGAATGCATTAGAAACA	40	((((((((((((((((((****))))))))))))))))))	-25.5	II	GTAATCCGAG	1	2
LY28_RS04920-LY28_RS04930	LY28_RS04920	ACCGGGGCATATGGGAATATGCTCCGGT	28	((((((((((((****))))))))))))	-19.6	IV	TGAAAAATA	1	2
LY28_RS05275-LY28_RS05285	LY28_RS05275	GGTGCGGTTGTAAGAGCTTTATATATAACCGCACC	35	((((((((((((***********))))))))))))	-21.8	II	TGCATTTCAG	3	4
LY28_RS07855-LY28_RS07865	LY28_RS07865	AATAGTTCTCCTCATATCAGAAATGATTGAGGAGAATTATT	41	((((((((((((((*((((****))))))))))))))))))	-23.2	III	ATTATTATAA	2	5
LY28_RS08815-LY28_RS08825	LY28_RS08815	ATAATTGGTTGGGTGCTTATGCGCGCATCATAAGCATCCAATCAATTAT	49	(((((((((((((((((((((*******)))))))))))))))))))))	-26.9	II	TTATTTTATC	4	7
LY28_RS09250-LY28_RS09260	LY28_RS09250	CACCTCGTGTTTCAGAACAGAAATTCAGGAATACGGCGGTG	41	((((((****((((((((*(((******)))*))))))))))))))	-16.3	III	ATAATATTAT	2	5
LY28_RS10460-LY28_RS10470	LY28_RS10460	TAAAATAACAGGCAGGAATAATTCCTGCCTGTTATTTTA	39	((((((((((((((((((***))))))))))))))))))	-25.6	II	TGTTATTTTA	3	7
LY28_RS10760-LY28_RS10770	LY28_RS10760	GGGCTGTTTCTTTTGAGGCAGCCT	24	((((((((((****))))))))))	-18.9	I	TTAGTTTTTG	5	7
LY28_RS11225-LY28_RS11235	LY28_RS11225	TGCACAGGAAGTCTTTTTACAGACTTCCTGTGCA	34	((((((((((((((******))))))))))))))	-24.5	II	AATAAATCTA	2	3

Table S3 The RNA secondary structure of intergenic regions between SBPs and their cognate translocator genes in *Escherichia coli*.

ABC importer Cluster	SBP gene	sequence of intergenetic region	folding energy(ΔG)
gltL-gltK-gltJ-gltI	gltL	CTGGCGCGCATACCCAATTGCGCGCCAT	-18.2
glnQ-glnP-glnH	glnH	CCCTCTCCCCTATGGGGAGAGGA	-17.9
potF-potG-potH-potI	potF	ATGCCGGAGGGGCGCACCACACCCGCCGGCAA	-17.7
oppA-oppB-oppC-oppD-oppF	oppA	ACGTGGGGCAGGAGTGTCCTGCTCCACGG	-22.8
araH-araG-araF	araF	TTCCCCTCTGCATGATGCAGAGGGGGT	-20.0
mglC-mglA-mglB	mglB	TGGGCGCAGTCTATTACTGCGCCCT	-17.8
hisP-hisM-hisQ-hisJ-argT	hisJ	CTCCCTTCGGGTAACCGGAGGGAGA	-19.8
ugpC-ugpE-ugpA-ugpB	ugpB	ATGTCGGATGCGTTTCGCTTATCTGACCTGGCATCGCGTGTAGGCCGGATAAG<br>CGAAGCGCATCCGGCAC	-49.0
livF-livG-livM-livH-livK	livK	ACCGCCCGTAAAATGCGGGCGGGT	-17.3
dppF-dppD-dppC-dppB-dppA	dppA	CCTACGAAAATTCTGCAATGTATTGAATTTGCACGATTTTGTAGG	-25.2
xylF-xylG-xylH	xylF	CGTTACGCCCCAGCGCGGAGCGGGGGCGTGATT	-21.1
pstB-pstA-pstC-pstS	pstS	GGCCGGGTACGGTGTTTTACGCCGCATCCGGCA	-21.8
malG-malF-malE	malE	ATGCCGGATGCGGCGTGAACGCCTTGTCCGGCC	-52.1
alsC-alsA-alsB	alsB	TGCCGGATGATGATGCTAATGAAGTGTCTTATCCGGCC	-20.3
ytfQ-ytfR-ytfT-yjfF	ytfQ	CGCGCCGCATCCGGCGATGGTGCACTGAAGCCTGATGCGACGCT	-21.7

Table S4 Bacterial strains and plasmids used in this study

Strains and plasmids	Relevant characteristic(s)	Source or references
**Strains**		
E.coli DH5α	*f80dlacZΔM*15, *Δ(lacZYA-argF)U*169, *endA*1, *recA*1 *hsdR*17*(rk- , mk+), supE*44, *lthi-1 gyrA*96, *relA*1, *phoA*	Transgene
R. cellulolytiucm H10Δ*mspI*	Derived from *Ruminiclostridium cellulolytiucm* H10 with the *mspI* deleted	Granted from Cui *et al* (4)
R. cellulolytiucm H10Δ*mspI* Δ2112	Derived from *Ruminiclostridium cellulolytiucm* H10Δ*mspI* with the Ccel_2112 deleted	This study
**Plasmids**		
pMTC6	Derived from pIMP1, containing *PpFbFpm*, *thl* promoter	Granted from Cui *et al* (4)
pMTC9	Derived from pMTC6, containing a dual fluorescence reporter system	This study
pSY6	MlS^R, Amp^R, *E.coli*-*C.cellulolyticum* shuttle vector, *ptb* promoter, contaning *L*.lactis L1.Ltr intron and *ltrA*	Shao *et al* (5)
pSY6-2112	Derived from pSY6, targeting the Ccel_2112 in *C.cellulolyticum*	This study
pSL-0885	pMTC9 derivative expression vector harboring SL-0885	This study
pSL-1223	pMTC9 derivative expression vector harboring SL-1223	This study
pSL-1025	pMTC9 derivative expression vector harboring SL-1025	This study
pSL-1252	pMTC9 derivative expression vector harboring SL-1252	This study
pSL-1768	pMTC9 derivative expression vector harboring SL-1768	This study
pSL-2997	pMTC9 derivative expression vector harboring SL-2997	This study
pSL-3246	pMTC9 derivative expression vector harboring SL-3246	This study

pSL-0150	pMTC9 derivative expression vector harboring SL-0150	This study
pSL-0998	pMTC9 derivative expression vector harboring SL-0998	This study
pSL-1133	pMTC9 derivative expression vector harboring SL-1133	This study
pSL-2112	pMTC9 derivative expression vector harboring SL-2112	This study
pSL-2589	pMTC9 derivative expression vector harboring SL-2589	This study
pSL-0200	pMTC9 derivative expression vector harboring SL-0200	This study
pSL-1406	pMTC9 derivative expression vector harboring SL-1406	This study
pSL-1987	pMTC9 derivative expression vector harboring SL-1987	This study
pSL-2687	pMTC9 derivative expression vector harboring SL-2687	This study
pSL-0885Δ3’tail	pMTC9 derivative expression vector harboring SL-0885 without 3’tail	This study
pSL-0885T1025	pMTC9 derivative expression vector harboring SL-0885 with U-rich tract of SL-1025	This study
pSL-0885T1252	pMTC9 derivative expression vector harboring SL-0885 with U-rich tract of SL-1252	This study
pSL-0885T2997	pMTC9 derivative expression vector harboring SL-0885 with U-rich tract of SL-2997	This study
pSL-0885T3246	pMTC9 derivative expression vector harboring SL-0885 with U-rich tract of SL-3246	This study
pSL-0885T0998	pMTC9 derivative expression vector harboring SL-0885 with U-rich tract of SL-0998	This study
pSL-0885T1133	pMTC9 derivative expression vector harboring SL-0885 with U-rich tract of SL-1133	This study
pSL-0885T2112	pMTC9 derivative expression vector harboring SL-0885 with U-rich tract of SL-2112	This study
pSL-0885T2589	pMTC9 derivative expression vector harboring SL-0885 with U-rich tract of SL-2589	This study
pSL-0885T1406	pMTC9 derivative expression vector harboring SL-0885 with U-rich tract of SL-1406	This study
pSL-0885T1987	pMTC9 derivative expression vector harboring SL-0885 with U-rich tract of SL-1987	This study
pSL-0885T2687	pMTC9 derivative expression vector harboring SL-0885 with U-rich tract of SL-2687	This study
pSL-2997Δ5-5'	pMTC9 derivative expression vector harboring SL-2997 deleted 5 nt at 5' end	This study
pSL-2997Δ9-5'	pMTC9 derivative expression vector harboring SL-2997 deleted 9 nt at 5' end	This study
pSL-1025Δ8-5'	pMTC9 derivative expression vector harboring SL-1025 deleted 8 nt at 5' end	This study
pSL-1252Δ4-5'	pMTC9 derivative expression vector harboring SL-1252 deleted 4 nt at 5' end	This study
pSL-2112Δ6-5'	pMTC9 derivative expression vector harboring SL-2112 deleted 6 nt at 5' end	This study

pSL-2589Δ7-5'	pMTC9 derivative expression vector harboring SL-2589 deleted 7 nt at 5' end	This study
pSL-1406Δ7-5'	pMTC9 derivative expression vector harboring SL-1406 deleted 7 nt at 5' end	This study
pSL-1406Δ16-5'	pMTC9 derivative expression vector harboring SL-1406 deleted 16 nt at 5' end	This study
pSL-1406Δ11-5'	pMTC9 derivative expression vector harboring SL-1406 deleted 11 nt at 5' end	This study
pSL-2112-2	pMTC9 derivative expression vector harboring SL-2112 deleted 2 base pairs on top of stem	This study
pSL-2112-4	pMTC9 derivative expression vector harboring SL-2112 deleted 4 base pairs on top of stem	This study
pSL-2112+2	pMTC9 derivative expression vector harboring SL-2112 added 2 base pairs on top of stem	This study
pSL-2112+4	pMTC9 derivative expression vector harboring SL-2112 added 4 base pairs on top of stem	This study
pSL-2112U8A	pMTC9 derivative expression vector harboring SL-2112 mutated U8 to A	This study
pSL-2589A9C	pMTC9 derivative expression vector harboring SL-2589 mutated A9 to C	This study
pSL-2687C13U	pMTC9 derivative expression vector harboring SL-2687 mutated C13 to U	This study
pMTC6-P0044	pMTC6 derivative expression vector harboring promoter sequence of Ccel_0044	This study
pMTC6-P0048	pMTC6 derivative expression vector harboring promoter sequence of Ccel_0048	This study

Table S5 Primers used in this study

Primers	Sequences (5'_3')	Description
For generating and validating the mutants		
Ccel_2112-IBS	CCGCTCGAGATAATTATCCTTACAGCACCTAGCAGTGCGCCCAGATAGGGTG	To construct a targeting region for Ccel_2112 by SOEing PCR
Ccel_2112-EBS2	TGAACGCAAGTTTCTAATTTCGGTTTGCTGTCGATAGAGGAAAGTGTCT	
Ccel_2112-EBS1d	AGATTGTACAAATGTGGTGATAACAGATAAGTCCTAGCAGTTAACTTACCTTTCTTTGT	
EBS universal primer	CGAAATTAGAAACTTGCGTTCAGTAAAC	
Ccel_2112-F	ATGTTTAAAAAGGTAATAGC	To validate the mutant of Ccel_2112
Ccel_2112-R	TTACTTCAATTCAGGAACAT	
For Real-time quantitative RT_PCR (qPCR)		
Ccel_2110_F	ATCAGGAACAATCAACGCAAT	Intragenic region of Ccel_2110, qRT_PCR
Ccel_2110_R	CTCCATCAACACGTGCAG	
Ccel_2111_F	ATCAGGAACAATCAACGCAAT	Intragenic region of Ccel_2111 qRT_PCR
Ccel_2111_R	CTCCATCAACACGTGCAG	
Ccel_2112_F	ATGCATCATTCGCAAAGAGC	Intragenic region of Ccel_2112, qRT_PCR
Ccel_2112_R	AATGTATCCCCACGCTGCATC	
Ccel_0312_F	AGATACTAAGCTCGGTCCTGA	Intragenic region of Ccel_0312, qRT_PCR
Ccel_0312_R	CACCAAAGATCGCTCTAAGCA	
fbfp_F	CATGATCAGCCTGGTATAGC	Intragenic region of *fbfp*, qRT_PCR
fbfp_R	TCTTCTGCAAATACCTGTGCT	
mcherry_F	ATTTTCCTTCAGATGGACCTGT	Intragenic region of *mcherry*, qRT_PCR

mcherry_R	ATTATATGCTCCAGGAAGCTGT	
For Northern blotting		
fbfp_F	ATGATAAATGCAAAACTTCTTCAGC	To prepare probe for *fbfp*
fbfp_R	TTAATGTTTTGCCTGACCCTG	
mcherry_F	ATGGTATCAAAAGGAGAAGAAGATA	To prepare probe for *mcherry*
mcherry_R	TTATTTATAAAGTTCATCCATT	
Ccel_2111_F	TTAGATTGCTCCATCTTTAT	To prepare probe for Ccel_2111
Ccel_2111_R	ATGAAAAATCTCTCAAAGAAA	
Ccel_2112_F	TTACTTCAATTCAGGAACAT	To prepare probe for Ccel_2112
Ccel_2112_R	ATGTTTAAAAAGGTAATAGC	
For Northern blotting DNA Maker		
M2112-2110_F	ATGTTTAAAAAGGTAATAGCTTC	To show the length of transcripts of Ccel_2112-2110
M2112-2110_500bp_R	GCTCCTAATACCTGTCCC	
M2112-2110_1000bp_R	CCTTCCAGATTGACAATG	
M2112-2110_1500bp_R	GCCATGAAAAGATAAGGTG	
M2112-2110_2000bp_R	CAAAACCGGCTATAAAGAAC	
M2112-2110_3000bp_R	GCGGTAACAGATATGAATTC	
M2112-2110_4000bp_R	AATCCAAGGATAAGGTGTTTTAG	
M_fbfp-mcherry_F	ATGATAAATGCAAAACTTCTTCAGC	To show the length of transcripts of *fbfp-mcherry* operons with various stem-loop structures
M_fbfp-mcherry_200bp_R	GCTATACCAGGCTGATCATGAT	
M_fbfp-mcherry_400bp_R	TTCTAAGTTCTGCTACTTCTG	
M_fbfp-mcherry_600bp_R	ACTGATCCTTCCATATGTACTTT	
M_fbfp-mcherry_800bp_R	CAGGAAATGAAAGTTTAAGATAA	

M_fbfp-mcherry_1000bp_R	TCCATCTTCAGGATACATTCTTT	
M_fbfp-mcherry_1200bp_R	GAATGTCTTCCTTCTGCTCTTTC	
M_fbfp-mcherry_1500bp_R	CCTCTGACACATGCAGCTCCCGG	
For cloning of stem-loops		
SL-0885_F	GGAAGATCTAGGGCTGATATCTTATGTAT	To amplify SL-0885
SL-0885_R	CTTCTCCTTTTGATACCATCAAAAAAATGGGCCGATACA	
SL-1223_F	GGAAGATCTTAAAAAACAGGTGTGAAACA	To amplify SL-1223
SL-1223_R	CTTCTCCTTTTGATACCATTAAAAAAACAGGTGTGAATG	
SL-1025_F	GGAAGATCTATGATAATACAGGTGTTCGG	To amplify SL-1025
SL-1025_R	CTTCTCCTTTTGATACCATTTGATAATACAGATGTCCTT	
SL-1252_F	GGAAGATCTTAGAAGGAAAGGATACAGCC	To amplify SL-1252
SL-1252_R	CTTCTCCTTTTGATACCATTAAACGAGAAGGAAAGGATA	
SL-1768_F	GGAAGATCTTGAGAATGTCCGTCATTAAA	To amplify SL-1768
SL-1768_R	CTTCTCCTTTTGATACCATTAAAGAGAATGCCCGTTCAT	
SL-2997_F	GGAAGATCTTTTCAACAAAGGGGAAATAT	To amplify SL-2997
SL-2997_R	CTTCTCCTTTTGATACCATTAAATTCAACAAAGGGGAAA	
SL-3246_F	GGAAGATCTGTAATACAATTCTCCTCGAA	To amplify SL-3246
SL-3246_R	CTTCTCCTTTTGATACCATTAAAATACAATTCTCCTCGT	
SL-0150_F	GGAAGATCTGTTAAAGTCCATTGGAGCAT	To amplify SL-0150
SL-0150_R	CTTCTCCTTTTGATACCATTTTAAATATCCATTGGAGCA	
SL-0998_F	GGAAGATCTAAACCATCCCTGTATGGGTT	To amplify SL-0998
SL-0998_R	CTTCTCCTTTTGATACCATTAACCAATCCCTGTATGAGA	
SL-1133_F	GGAAGATCTAGTATAATATGATTGGGTAC	To amplify SL-1133
SL-1133_R	CTTCTCCTTTTGATACCATCGTATAAATTGATTGGGTAC	

SL-2112_F	GGAAGATCTTATGATTTAGGAGAGGGTGA	To amplify SL-2112
SL-2112_R	CTTCTCCTTTTGATACCATTAAAATAATCAAGGAGAGGG	
SL-2589_F	GGAAGATCTGCCAAAGCAAAGGGGATGTC	To amplify SL-2589
SL-2589_R	CTTCTCCTTTTGATACCATCAGAAACAAAACACAGGGGTG	
SL-0200_F	GGAAGATCTAGGCGTCCATTTGTTTGCTTT	To amplify SL-0200
SL-0200_R	CTTCTCCTTTTGATACCATTGGCGTCCATTTCTTTGCCA	
SL-1406_F	GGAAGATCTATTTAGCAGTAGTTGATAGA	To amplify SL-1406
SL-1406_R	CTTCTCCTTTTGATACCATTTTTAGCAGTAGTCGATTAC	
SL-1987_F	GGAAGATCTGGTGTTCGGTTTGCATATGT	To amplify SL-1987
SL-1987_R	CTTCTCCTTTTGATACCATTGTATTCGGCTTGCATATATG	
SL-2687_F	GGAAGATCTATATATGAAGTACGCCATGC	To amplify SL-2687
SL-2687_R	CTTCTCCTTTTGATACCATCTATATGAAGTATGCCAAGC	
mcherry_F	ATGGTATCAAAAGGAGAAG	To amplify the DNA fragment of *mcherry*
mcherry_R	CGGAATTCTTATTTATAAAGTTCATCC	
For replacement of 3’tail of SL-0885		
SL-0885Δ3’tail_F	GGAAGATCTAGGGCTGATATCTTATGTATCGGCCCATGGTATCAAAAGGAGAAGA	To amplify SL-0885 without 3' U-rich tail
mcherry_R	CGGAATTCTTATTTATAAAGTTCATCC	
SL-0885_F	GGAAGATCTAGGGCTGATATCTTATGTAT	Upstream primer
SL-0885T1025_R	CTTCTCCTTTTGATACCATTTGATAATAGGGCCGATACATA	To fuse SL-0885 with U-rich tract of SL-1025
SL-0885T1252_R	CTTCTCCTTTTGATACCATTAAACGAGAAGGGCCGATACATA	To fuse SL-0885 with U-rich tract of SL-1252
SL-0885T2997_R	CTTCTCCTTTTGATACCATTAAATTCAACAAAGGGCCGATACAT	To fuse SL-0885 with U-rich tract of

	A	SL-2997
SL-0885T3246_R	CTTCTCCTTTTGATACCATTAAAATACAAGGGCCGATACATA	To fuse SL-0885 with U-rich tract of SL-3246
SL-0885T0998_R	CTTCTCCTTTTGATACCATTAACCAATGGGCCGATACATA	To fuse SL-0885 with U-rich tract of SL-0998
SL-0885T1133_R	CTTCTCCTTTTGATACCATCGTATAAATTGGGCCGATACATA	To fuse SL-0885 with U-rich tract of SL-1133
SL-0885T2112_R	CTTCTCCTTTTGATACCATTAAAATAATCAAGGGCCGATACATA	To fuse SL-0885 with U-rich tract of SL-2112
SL-0885T2589_R	CTTCTCCTTTTGATACCATCAGAAACAAAACACAGGGCCGATACATA	To fuse SL-0885 with U-rich tract of SL-2589
SL-0885T1406_R	CTTCTCCTTTTGATACCATTTTTAGCAGTAGTCGATTAGGGCCGATACATA	To fuse SL-0885 with U-rich tract of SL-1406
SL-0885T1987_R	CTTCTCCTTTTGATACCATTGTATTCGGCTTGCATATATGGGCCGATACATA	To fuse SL-0885 with U-rich tract of SL-1987
SL-0885T2687_R	CTTCTCCTTTTGATACCATCTATATGAAGTATGGGCCGATACATA	To fuse SL-0885 with U-rich tract of SL-2687
For deleting the 5’sequence of the stem-loops		
SL-2997Δ5-5’_F1	GGGGAAATATTTTCCCCTTTGTTGAATTTAATGGTATCAAAAGGAGAAGA	To amplify the stem-loop of SL-2997 deleted 5 nr at 5' end
SL-2997Δ5-5’_F2	GGAAGATCTTAAAGGGGAAATATTTTCCC	
SL-2997Δ9-5’_F1	GGGGAAATATTTTCCCCTTTGTTGAATTTAATGGTATCAAAAGGAGAAGA	To amplify the stem-loop of SL-2997 deleted 9 nt at 5' end
SL-2997Δ9-5’_F2	GGAAGATCTTGGGGAAATATTTTCCCCTT	
SL-1025Δ8-5’_F1	TGTTCGGAAGGACATCTGTATTATCAAATGGTATCAAAAGGAGAAGA	To amplify the stem-loop of SL-1025 deleted 8 nt at 5' end

SL-1025Δ8-5'_F2	GGAAGATCTCAGGTGTTCGGAAGGACATCTGTA	
SL-1252Δ4-5'_F1	GGATACAGCCGTTTCATATCCTTTCCTTCTCGTTTAATGGTATCAAAAGGAGAAGA	To amplify the stem-loop of SL-1252 deleted 4 nt at 5' end
SL-1252Δ4-5'_F2	GGAAGATCTTGGAAAGGATACAGCCGTTTCATATC	
SL-2112Δ6-5'_F1	GAGGGTGAAATTCCCTCTCCTTGATTATTTAATGGTATCAAAAGGAGAAGA	To amplify the stem-loop of SL-2112 deleted 6 nt at 5' end
SL-2112Δ6-5'_F2	GGAAGATCTTAGGAGAGGGTGAAATTCCCTCTCC	
SL-2589Δ7-5'_F1	ATGTCCAAGGGCACCCCTGTGTTTTGTTTCTGATGGTATCAAAAGGAGAAGA	To amplify the stem-loop of SL-2589 deleted 7 nt at 5' end
SL-2589Δ7-5'_F2	GGAAGATCTGAGGGGATGTCCAAGGGCACCCCTGT	
SL-1406Δ7-5'_F1	AGAGCCGATTTAGTCGGTAATCGACTACTGCTAAAAATGGTATCAAAAGGAGAAGA	To amplify the stem-loop of SL-1406 deleted 7 nt at 5' end
SL-1406Δ7-5'_F2	GGAAGATCTATAGTTGATAGAGCCGATTTAGTCGGTAA	
SL-1406Δ16-5'_F1	CGATTTAGTCGGTAATCGACTACTGCTAAAAATGGTATCAAAAGGAGAAGA	To amplify the stem-loop of SL-1406 deleted 16 nt at 5' end
SL-1406Δ16-5'_F2	GGAAGATCTAAGAGCCGATTTAGTCGGTAATCGACT	
SL-2687Δ11-5'_F1	GCAATAGCTTGGCATACTTCATATAGATGGTATCAAAAGGAGAAGA	To amplify the stem-loop of SL-2687 deleted 11 nt at 5' end
SL-2687Δ11-5'_F2	GGAAGATCTCGCCATGCAATAGCTTGGCATACTTC	
mcherry_R	CGGAATTCTTATTTATAAAGTTCATCC	Downstream primer
For shortening or lengthening stem of SL-2112		
SL-2112-2_F1	TTTAGGAGGGTGAAATTCCCTCCTTGATTATTTAATGGTATCAAAAGGAGAAGA	To delete 2 base pairs on top of stem of SL-2112
SL-2112-2_F2	GGAAGATCTTATGATTTAGGAGGGTGAAATT	
SL-2112-4_F	GGAAGATCTTATGATTTAGGAGTGAAATTCTCCTTGATTATTTT	To delete 4 base pairs on top of stem of

	AATGGTATCAAAAGG	SL-2112
SL-2112+2_F1	GGGTATGAAATTTACCCTCTCCTTGATTATTTTAATGGTATCAA AAGGAGAAGA	To add 2 base pairs on top of stem of SL-2112
SL-2112+2_F2	GGAAGATCTTATGATTTAGGAGAGGGTATGAAATTTACCCT	
SL-2112+4_F1	GGGTACCTGAAATTGGTACCCTCTCCTTGATTATTTTAATGGTA TCAAAGGAGAAGA	To add 4 base pairs on top of stem of SL-2112
SL-2112+4_F2	GGAAGATCTTATGATTTAGGAGAGGGTACCTGAAATTGGTACC	
mcherry_R	CGGAATTCTTATTTATAAAGTTCATCC	Downstream primer
For mutation of unpaired regions of SL-2112, SL-2589, and SL-2687		
SL-2112U8A_F1	TAAGGAGAGGGTGAAATTCCCTCTCCTTGATTATTTTAATGGTA TCAAAAGGAGAAGA	To mutate U8 of SL-2112 to A
SL-2112U8A_F2	GGAAGATCTTATGATTAAGGAGAGGGTGAAATTCC	
SL-2589A9C_F1	GGGATGTCCAAGGGCACCCCTGTGTTTTGTTTCTGATGGTATCA AAAGGAGAAGA	To mutate A9 of SL-2589 to C
SL-2589A9C_F2	GGAAGATCTGCCAAAGCACAGGGGATGTCCAAGGGCACCCC	
SL-2687C13U_F1	AAGTATGCCATGCAATAGCTTGGCATACTTCATATAGATGGTAT CAAAAGGAGAAGA	To mutate C13 of SL-2687 to U
SL-2687C13U_F2	GGAAGATCTATATATGAAGTATGCCATGCAATAGCT	
mcherry_R	CGGAATTCTTATTTATAAAGTTCATCC	Downstream primer
For measuring the promoter activity		
P0044_F	AACTGCAGTCGATTAAATTTTTCCATAT	To generate promoter P0044
P0044_R	CGACGCGTTTTTGAATACCCCCTATTAT	
P0048_F	AACTGCAGACATTTTTTGATATCTAAAG	To generate promoter P0048
P0048_R	CGACGCGTAAAAACCTCCTAACCGCGAA	

References

1. Reese MG. 2001. Application of a time-delay neural network to promoter annotation in the Drosophila melanogaster genome. Computers & chemistry 26:51-56.
2. Janczarek M, Rachwał K, Kopcińska J. 2015. Genetic characterization of the Pss region and the role of PssS in exopolysaccharide production and symbiosis of Rhizobium leguminosarum bv. trifolii with clover. Plant and Soil 396:257-275.
3. Zuker M. 2003. Mfold Web Server for Nucleic Acid Folding and Hybridization Prediction. Nucleic Acids Research 31:3406-3415.
4. Cui GZ, Hong W, Zhang J, Li WL, Cui Q. 2012. Targeted gene engineering in Clostridium cellulolyticum H10 without methylation. Journal of Microbiological Methods 89:201-208.
5. Shao L, Hu S, Yang Y, Gu Y, Chen J, Yang Y, Jiang W, Yang S. 2007. Targeted gene disruption by use of a group II intron (targetron) vector in Clostridium acetobutylicum. Cell research 17:963-965.
